# Supplementary material for: Whole genome sequence analysis of pulmonary function and COPD in 19,996 multi-ethnic participants
Source: Nat Commun. 2020 Oct 14;11:5182. doi: 10.1038/s41467-020-18334-7 (PMC7598941; doi:10.1038/s41467-020-18334-7)
Supplement: Supplementary file 1 — Supplementary Information [file 41467_2020_18334_MOESM1_ESM.pdf]

Supplementary Information

Whole Genome Sequence Analysis of Pulmonary Function and COPD in 19,996 Multi-ethnic Participants

Zhao *et al.*

File includes Supplementary Methods, Supplementary Notes, Supplementary Tables, Supplementary Figures, Supplementary References

Supplementary Methods

Phenotype Harmonization

The NHLBI Pooled Cohorts Study (PCS)[1] harmonized and pooled data from nine large US epidemiologic cohorts that conducted lung function assessments over the last four decades. Additionally, data on self-administered questionnaires, with detailed questions regarding tobacco consumption, past medical history, medications, respiratory symptoms, lipids, renal biomarkers, etc., were also harmonized. Data on CLRD events was harmonized using either adjudicated CLRD hospitalizations or ICD data for all hospitalizations occurring over follow-up.

Among the cohorts included in the NHLBI PCS, the follow cohorts were also included in our TOPMed WGS analyses, for which we utilized harmonized data sets from NHLBI PCS: Atherosclerosis Risk in Communities (ARIC) study; Cardiovascular Health Study (CHS); Framingham Offspring Cohort (FHS-O); Jackson Heart Study (JHS), the Multi-Ethnic Study of Atherosclerosis (MESA). For the purpose of phenotype harmonization in TOPMed, harmonized data sets for each of the participating cohorts were provided by the NHLBI Pooled Cohorts Study for analyses in the current WGS analyses. We note that a subset of the ARIC participants were later recruited into JHS. For TOPMed purposes, participants in the ARIC-JHS overlap group were not included as part of Exam 4 in ARIC. For JHS, ARIC recruits were not excluded. For TOPMed studies not included in the NHLBI Pooled Cohorts Study (the Cleveland Family Study [CFS], Genetic Epidemiology of COPD [COPDGene], and Boston Early-Onset COPD Study [EOCOPD]), phenotype harmonization was conducted separately by each of the participating studies, following as closely as possible with the NHLBI Pooled Cohorts Study variable definitions and procedures.

For studies with multiple longitudinal spirometry measures, we worked with investigators from each study to determine the most practical way to construct a cross-sectional subset of data. All spirometry data utilized for this effort were obtained as pre-bronchodilator measures. Details are provided in the cohort descriptions below.

Cohort Descriptions: Population- and Family-based Cohorts

The Atherosclerosis Risk in Communities Study (ARIC)

ARIC study is a prospective cohort study designed to evaluate the etiology of atherosclerosis and its clinical sequelae in a general population based sample of adults.[2] Men and women, aged 45–64 years, were recruited and enrolled from four U.S. communities: Forsyth County, North Carolina; Jackson, Mississippi; suburbs of Minneapolis, Minnesota; and Washington County, Maryland. Initial examination of the cohort took place in 1987–1989 (visit 1), when participants responded to health-related questionnaires and completed a clinical examination. Cohort members completed three additional triennial follow-up examinations, a fifth exam in 2011-2013, and a sixth exam in 2016-2017.

At each visit, spirometry testing protocols were standardized across the four ARIC field centers, calibration checks were performed daily, and the standardization of

data collection and management was coordinated across field centers by a single pulmonary function reading center. Each participant's best FEV_1 and FVC of three acceptable maneuvers, based on the centralized expert review, was used for analysis.[3] For the current cross-sectional WGS analysis, data from the most recent spirometry exam were utilized for participants having multiple longitudinal measures, as these measures were viewed to be the most consistent with those available for the other more contemporary cohorts.

The Cardiovascular Health Study (CHS)

CHS is a population-based cohort study of risk factors for coronary heart disease and stroke in adults ≥65 years conducted across four field centers.[4] The original predominantly European ancestry cohort of 5,201 persons was recruited in 1989-1990 from random samples of the Medicare eligibility lists; subsequently, an additional predominantly African-American cohort of 687 persons was enrolled for a total sample of 5,888. Forty one European ancestry CHS participants that had been selected for inclusion in the second phase of the TOPMed sequencing program were used included in our discovery analyses.

Blood samples were drawn from all participants at their baseline examination and DNA was subsequently extracted from available samples. CHS was approved by institutional review committees at each field center and individuals in the present analysis had available DNA and gave informed consent including consent to use of genetic information for the study of cardiovascular disease. Pulmonary function testing was conducted at the baseline visit and follow-up visits in years four and seven.[5,6] Spirometry technicians were centrally trained and certified prior to recruitment of participants. A standard spirometry system, including a Collins Survey I water-seal spirometer (Collins Medical, Inc., Braintree, Massachusetts) and software from S&M Instruments (Doylestown, Pennsylvania), was used by technicians at all four recruitment centers. Stringent quality assurance procedures for spirometry testing exceeded ATS recommendations.[5] For the current cross-sectional WGS analysis, data from the most recent spirometry exam were utilized for participants having multiple longitudinal measures, as these measures were viewed to be the most consistent with those available for the other more contemporary cohorts.

The Cleveland Family Study (CFS)

CFS is a family-based longitudinal study that includes participants with laboratory diagnosed sleep apnea, their family members and neighborhood control families followed between 1990 and 2006. Four examinations over 16 years provided measurements of sleep apnea with overnight polysomnography, anthropometry, and other related phenotypes.[7] At each exam, forced vital capacity (FVC) and forced expiratory volume at one second (FEV_1) were obtained using a calibrated spirometer (Multi-Spiro). While seated, participants were encouraged to perform between 5-8 maneuvers to obtain 3 curves that met ATS standards for acceptability and reproducibility. For the current cross-sectional WGS analysis, data from the most recent spirometry exam were utilized for participants having multiple longitudinal measures, since the most recent exam provided the largest number of participants with non-missing spirometry data.

The Framingham Heart Study (FHS)

The Original Cohort of the Framingham Study was established between 1948 and 1952 as a random sample of 5,209 adult residents of the town of Framingham, Massachusetts. Between 1971 and 1975, the Framingham Study was expanded to include a second generation, the Offspring Cohort, comprising 5,124 adults who were the offspring, or spouses of the offspring, of Original Cohort participants.[8] The Offspring Cohort has returned for examinations approximately every 4 years since enrollment, and spirometry data are available for the 3rd, 5th, 6th, 7th, and 8th examinations.

Spirometry for the Offspring Cohort 3rd examination (1983-87) was performed with a Collins Survey II spirometer interfaced with an Eagle II microprocessor (Warren E. Collins, Inc., Braintree, MA). Spirometry for the 5th (1991-95), 6th (1995-98), and 7th (1998-2001) examinations were performed with a Collins Survey II spirometer interfaced with a personal computer equipped with software developed by S & M Instruments (Doylestown, PA) and adapted for use in epidemiologic studies. Spirometry for the 8th (2005-08) and 9th (2011-14) examinations was performed with the Collins Comprehensive Pulmonary Laboratory (CPL) system with Collins 2000 Plus/SQL Software (Nspire Health, Inc., Longmont, CO). Spirometry was performed in accordance with contemporaneous guidelines of the American Thoracic Society. For the current cross-sectional WGS analysis, data from the earliest spirometry exam were utilized for participants having multiple longitudinal measures. We used this strategy to construct a cross-sectional subset because there were changes in the spirometers used in FHS over time, and selecting the earliest available spirometry measures for each participant was a practical way to create an internally consistent data set within this cohort.

The Jackson Heart Study (JHS)

JHS is a large, population-based observational study evaluating the etiology of cardiovascular diseases and related disorders among African Americans residing in the three counties (Hinds, Madison, and Rankin) that make up the Jackson, Mississippi metropolitan area.[9,10] Data and biologic materials have been collected from 5,301 participants, including a nested family cohort of 1,498 members of 264 families. The age at enrollment for the unrelated cohort was 35-84 years; the family cohort included related individuals >21 years old. During a baseline examination (2000-2004) and two follow-up examinations (2005-2008 and 2009-2012), participants provided extensive medical and social history, had an array of physical and biochemical measurements and diagnostic procedures, and provided blood for genomic DNA.[11] The study population is characterized by a high prevalence of diabetes, hypertension, obesity, and related disorders. Annual follow-up interviews and cohort surveillance are ongoing. For the current cross-sectional WGS effort, only baseline spirometry data were available and utilized for analyses.

The Multi-Ethnic Study of Atherosclerosis (MESA)

MESA is a longitudinal study of subclinical cardiovascular disease and risk factors that predict progression to clinically overt cardiovascular disease or progression of the subclinical disease.[12] Between 2000 and 2002, MESA recruited 6,814 men and women 45 to 84 years of age from Forsyth County, North Carolina; New York City; Baltimore;

St. Paul, Minnesota; Chicago; and Los Angeles. Exclusion criteria were clinical cardiovascular disease, weight exceeding 136 kg (300 lb.), pregnancy, and impediment to long-term participation. The MESA Lung Study performed spirometry at Exams 3, 4 and 5 following the 2005 ATS/ERS guidelines in a subset of the MESA Study.[13] All participants provided informed consent and the protocols of MESA were approved by the IRBs of collaborating institutions and the National Heart, Lung and Blood Institute. For the current cross-sectional WGS analysis, data from the earliest spirometry exam were utilized for participants having multiple longitudinal measures, as the measures from Exams 3 and 4 were viewed to be more internally consistent with each other, and only a smaller subset of participants had measures available at Exam 5.

Cohort Descriptions: COPD-enriched studies

Boston Early-Onset COPD (EOCOPD) Study
The Boston Early-Onset COPD (EOCOPD) study was designed to study genetic factors for early-onset and severe COPD.[14] Probands were selected to be physician-diagnosed COPD cases with $FEV_1 \leq 40\%$ predicted and age ≤ 53. Subjects with severe alpha-1 antitrypsin deficiency and other chronic lung diseases (except asthma) were excluded. All subjects completed a questionnaire and spirometry testing before and after bronchodilator administration. Blood samples and written informed consent were obtained for each study subject. A subset of the most severe unrelated probands from this study were sent for whole-genome sequencing through the TOPMed project. For the current cross-sectional WGS effort, only baseline spirometry data were available and utilized for analyses.

Genetic Epidemiology of COPD (COPDGene)
COPDGene[15] is a multi-center observational cohort for epidemiologic and genetic study of over 10,000 subjects (2/3 non-Hispanic White and 1/3 African Americans) with at least 10 pack-years of cigarettes smoking with and without COPD. All subjects underwent extensive phenotyping, including lung function, CT phenotypes (including emphysema and expiratory gas trapping). Pre- and post-bronchodilator spirometry measures were obtained using a standardized protocol and spirometer (ndd EasyOne Spirometer, Zurich, Switzerland). All study sites obtained local IRB approval to enroll participants and all subjects provided informed consent. For the current cross-sectional WGS effort, only baseline spirometry data were utilized for analyses, as the baseline data were complete and only a subset of participants had follow-up data.

Quality control of samples and variants included in our TOPMed pooled cohorts WGS analysis
For the pooled cohort, we first removed subjects who failed sample-level quality control. The filters included checking for pedigree errors, discrepancies between self-reported and genetic sex, and concordance between prior SNP array genotypes and WGS-derived genotypes (see https://www.nhlbiwgs.org/topmed-whole-genome-sequencing-

project-freeze-5b-phases-1-and-2). In addition, duplicated subjects were identified using ~250k independent markers. Only one subject from each pair of duplicates was kept. There were 19,996 subjects of the pooled cohort that passed the filtering.

For site-level quality control, variants were removed based on Mendelian discordance, a support vector machine (SVM) quality filter and excess heterozygosity filter (see https://www.nhlbiwgs.org/topmed-whole-genome-sequencing-project-freeze-5b-phases-1-and-2). After filtering on variant level quality control and expected heterozygosity count > 30, there were 28,740,775 variants remaining for analysis.

Comparison of TOPMed WGS calls with genotypes imputed by GWAS in MESA
We examined the R-squared of genotypes for the novel associated variants using variant calls from TOPMed Freeze 5b compared to genotypes obtained using imputation of genome-wide genotypes in MESA to various reference panels including the 1000 Genomes Phase 1[16], 1000 Genomes Phase 3[17], the Haplotype Reference Consortium[18] , and the TOPMed reference panel. R-squared between TOPMed called genotypes and imputed genotype dosage values was computed as the square of the Pearson correlation.

Genome-wide genotyping in MESA: Participants in the original MESA cohort, the MESA Family Study and the MESA Air Pollution Study who consented to genetic analyses were genotyped in 2009 using the Affymetrix Human SNP array 6.0. Genotype quality control for these data included filter on SNP level call rate < 95%, individual level call rate < 95%, heterozygosity > 53%. The cleaned genotypic data was deposited with MESA phenotypic data into dbGaP as the MESA SHARe project (study accession phs000209, http://www.ncbi.nlm.nih.gov/projects/gap/cgi-bin/study.cgi?study_id=phs000209.v7.p2); 8,224 consenting individuals (2,685 White, 2,588 non-Hispanic African-American, 2,174 Hispanic, 777 Chinese) were included, with 897,981 SNPs passing study specific quality control (QC).

Genome-wide imputation in MESA: IMPUTE version 2.2.2 was used to perform imputation for the MESA SHARe genotypes to the cosmopolitan 1,000 Genomes Phase 1 v3 March 2012 reference set.[16] The University of Michigan imputation server was used for imputation of the MESA SHARe genotypes to the 1,000 Genomes Phase 3 integrated variant set[17] and the Haplotype Reference Consortium Release 1 data[18].

Replication studies
For those novel WGS variants identified in TOPMed for association with quantitative lung function traits (FEV_1, FVC and FEV_1/FVC ratio), we pursued replication for the same traits in multiple independent cohorts (described below). Variants identified for associations with moderate-to-severe or severe COPD in TOPMed were also examined for association with FEV_1/FVC ratio in the replication cohorts. Variants were reported as demonstrating statistically significant replication if they achieve Bonferroni-corrected statistical significance under a family-wise error rate of 0.05, after accounting for the number of variants under consideration for each trait. For variants demonstrating evidence of replication in relation to lung function, we further examined their association with measures of smoking behavior in order to determine whether the replicated

associations reflected primary association with lung function, or indirect association due to smoking.

UK Biobank

The UK Biobank project[19] is a prospective cohort study of approximately 500,000 individuals from across the United Kingdom, aged between 40 and 69 at recruitment. Deep phenotypic information, health data, and biological samples have been collected for all participants. These include questionnaire on socio-demographic, lifestyle and health-related factors, physical measurements, blood, urine, and saliva samples at recruitment. Eye measures, electrocardiograph test, arterial stiffness and hearing test were performed during assessment visit. All subjects provided electronic signed consent for follow-up through linkage to their health-related records.

Spirometry phenotypes were cleaned and the resulting data were used to conduct a genome-wide association study in participants from the UK Biobank.[20] Replication of lung function signals was examined using imputation to the Haplotype Reference Consortium (HRC)[18] in the 321,047 UK Biobank European samples and in 4,350 African UK Biobank samples. African ancestry samples were identified in UK Biobank by k-means clustering of the first two principal components of ancestry.[20] Residuals from linear regression of each trait (FEV_1, FVC, FEV_1/FVC) against age, age2, sex, height, smoking status (ever or never) and genotyping array were ranked and inverse-normal transformed, giving normally distributed Z-scores, and genetic association testing was run using an additive genetic model implemented in BOLT-LMM v2.3[21], using a linear mixed model to account for relatedness and fine-scale population structure. Lung function signals were also tested for association with smoking behaviour traits, including smoking initiation (SI), smoking cessation (SC) and heaviness of smoking index (HSI) in up to 447,062 European and 7,702 African UK Biobank samples. Genetic association testing of smoking behaviour traits was run under an additive genetic model using BOLT-LMM v2.320[21]. For binary smoking behaviour phenotypes, age, age squared, sex and genotyping array were used as covariates. For the quantitative HSI phenotype, residuals from linear regression against age, age squared, sex and genotyping array were ranked and inverse-normal transformed to obtain adjusted, normally distributed Z-scores. For replication analysis, we filter variants based on imputation R-squared > 0.3 and effective heterozygosity count > 15.

Hispanic Community Health Study / Study of Latinos (HCHS/SOL)

HCHS/SOL is a community-based cohort study of 16,415 self-identified Hispanic/Latino persons aged 18 to 74 years recruited from four U.S. communities.[22–24] Institutional Review Boards at each field center approved study protocols, and written informed consent was obtained from all participants. For the current replication effort, we examined results from a published GWAS of pulmonary function in 11,822 participants from the HCHS/SOL[25], performed using imputation to the 1000 Genomes Phase 1 v3[16]. Analyses of FEV_1, FVC and FEV_1/FVC ratio used linear mixed models, stratified by Hispanic/Latino ancestry group, and adjusted for age, age squared, sex, height, height squared, study center, smoking status, pack-years, sampling weights, and the first five PCs, as fixed effects. We used random effects for genetic relatedness (kinship) and

household and community (block unit) to account for environmental correlation. Results from each Hispanic/Latino ancestry group were then meta-analyzed using the MetaCor method.[26] Replication results are reported in the current manuscript after filter on imputation R-squared > 0.3 and effective minor heterozygosity count > 15.

Colocalization analysis

eQTL in GTEx v7: eQTL data used for the analyses described in this manuscript were obtained from the GTEx Portal on March 22, 2019 and represented sample sizes ranging from 70 to 491 per tissue, with 383 samples for lung. As the summary statistics for GTEx v7 were in human genome assembly hg19, the liftOver tool was used to map the coordinates to the same assembly.

eQTL and mQTL in MESA: RNA-seq was performed for PBMCs from MESA and profiling of genome-wide methylation was performed for whole blood using the Illumina EPIC array. eQTL and mQTL mapping was performed using tensorQTL[27] in ~900 individuals from MESA Exam 1 and Exam 5 data for variants with MAF ≥ 1%. The mapping window was set to +/- 1Mb of the TSS for eQTLs and +/- 500kb of the CpG site for mQTLs. We used 11 genotype PCs as covariates to control for population effects, and we used PEER factors[28] to control for both technical and biological variation. The optimal number of PEER factors to use was determined to maximize the *cis*-eGene and *cis*-mProbe discovery.

Colocalization analysis: Colocalization analysis of novel GWAS variants and eQTL/mQTL was performed using the R/coloc v3.1 package[29] with default priors (p1 = 1e-04, p2 = 1e-04, and p12 = 1e-05). Using the molecular QTL resources described above, we conducted colocalization analysis on all variants within +/-500kb of the novel WGS variants (reported in Supplementary Data 2), as well as those identified in conditional analysis (Supplementary Data 11). We kept only results where the lead WGS variant was a significant e/mQTL and posterior probability of colocalization (PP4) > 0.5. We further focused on results where the model of a single shared causal variant driving both association signals (PP4) was strongly preferred over a model of two distinct causal variants (PP3) - PP4/(PP3 + PP4) ≥ 0.9. In addition, we required adequate power for these results to detect colocalization, which we quantified using a cutoff of PP3 + PP4 ≥ 0.8.

Follow-up of selected methylation sites: Those methylation sites demonstrating colocalization with TOPMed WGS were examined further to determine whether measured methylation from MESA Exam 1 was associated with the corresponding lung function traits in MESA which were based on follow-up measures from MESA Exam 4. The association of measured methylation with lung function was tested in linear regression of the following form:

> PFT ~ methylation + age + age^2 + sex + height + height^2 + weight (for FVC only) + current smoking + former smoking + pack-years of smoking + genetic PCs of ancestry + methylation PEER factors.

We applied inverse normal transformation on both PFT trait and methylation levels of the colocalized methylation sites. We report methylation-lung function associations for

those results demonstrating Bonferroni-corrected significance accounting for the number of methylation markers tested.

Overlap with pathways previously implicated by GWAS: We examined genes in the following categories for overlap with pathways previously implicated by GWAS: (1) novel genes supported by eQTL colocalization, as indicated in Tables 2 and 3, (2) genes supported by findings in our TOPMed analysis that overlap previously reported GWAS signals, and (3) novel variants identified in our study that are located within gene introns. We selected for examination those gene ontology (GO) terms represented as enriched among genes implicated by GWAS in Supplementary Table 15 of the recent lung function GWAS paper by Shrine *et al*.[20] We then used the database of GO term inclusion provided at http://amigo.geneontology.org/ to report the overlap of our identified genes with the selected GO terms.

Supplementary Note 1

Study Specific Acknowledgments: Population- and Family-based Cohorts

The Atherosclerosis Risk in Communities Study
SJL is supported by the Intramural Research Program of the NIH, National Institute of Environmental Health Sciences (ZO1 ES043012). The Genome Sequencing Program (GSP) was funded by the National Human Genome Research Institute (NHGRI), the National Heart, Lung, and Blood Institute (NHLBI), and the National Eye Institute (NEI). The GSP Coordinating Center (U24 HG008956) contributed to cross-program scientific initiatives and provided logistical and general study coordination. The Centers for Common Disease Genomics (CCDG) program was supported by NHGRI and NHLBI, and whole genome sequencing was performed at the Baylor College of Medicine Human Genome Sequencing Center (UM1 HG008898 and R01HL059367).

The Atherosclerosis Risk in Communities study has been funded in whole or in part with Federal funds from the National Heart, Lung, and Blood Institute, National Institutes of Health, Department of Health and Human Services (contract numbers HHSN268201700001I, HHSN268201700002I, HHSN268201700003I, HHSN268201700004I and HHSN268201700005I). The authors thank the staff and participants of the ARIC study for their important contributions.

The Cardiovascular Health Study
This Cardiovascular Health Study (CHS) research was supported by NHLBI contracts HHSN268201200036C, HHSN268200800007C, HHSN268200960009C, HHSN268201800001C N01HC55222, N01HC85079, N01HC85080, N01HC85081, N01HC85082, N01HC85083, N01HC85086; and NHLBI grants U01HL080295, U01HL130114, R01HL087652, R01HL105756, R01HL103612, R01HL085251, and R01HL120393 with additional contribution from the National Institute of Neurological Disorders and Stroke (NINDS). Additional support was provided through R01AG023629 from the National Institute on Aging (NIA). A full list of principal CHS investigators and institutions can be found at CHS-NHLBI.org.

The Cleveland Family Study
The Cleveland Family Study and SR were supported by NIH grants HL 046389, HL113338, and 1R35HL135818. BC is supported by the NIH grant K01 HL135405 and an American Thoracic Society Foundation Unrestricted Grant (Sleep) (http://foundation.thoracic.org).

The Framingham Heart Study
The Framingham Heart Study (FHS) acknowledges the support of contracts NO1-HC-25195, HHSN268201500001I and 75N92019D00031 from the National Heart, Lung and Blood Institute and grant supplement R01 HL092577-06S1 for this research. We also acknowledge the dedication of the FHS study participants without whom this research would not be possible. Dr. Vasan is supported in part by the Evans Medical Foundation and the Jay and Louis Coffman Endowment from the Department of Medicine, Boston University School of Medicine.

The Jackson Heart Study
The Jackson Heart Study (JHS) is supported and conducted in collaboration with Jackson State University (HHSN268201800013I), Tougaloo College (HHSN268201800014I), the Mississippi State Department of Health (HHSN268201800015I) and the University of Mississippi Medical Center (HHSN268201800010I, HHSN268201800011I and HHSN268201800012I) contracts from the National Heart, Lung, and Blood Institute (NHLBI) and the National Institute on Minority Health and Health Disparities (NIMHD).

The Multi-Ethnic Study of Atherosclerosis
MESA and the MESA SHARe project are conducted and supported by the National Heart, Lung, and Blood Institute (NHLBI) in collaboration with MESA investigators. The MESA Lung Study is supported by R01-HL077612 and R01-HL093081. Support for MESA is provided by contracts 75N92020D00001, HHSN268201500003I, N01-HC-95159, 75N92020D00005, N01-HC-95160, 75N92020D00002, N01-HC-95161, 75N92020D00003, N01-HC-95162, 75N92020D00006, N01-HC-95163, 75N92020D00004, N01-HC-95164, 75N92020D00007, N01-HC-95165, N01-HC-95166, N01-HC-95167, N01-HC-95168, N01-HC-95169, UL1-TR-000040, UL1-TR-001079, and UL1-TR-001420. Also supported by the National Center for Advancing Translational Sciences, CTSI grant UL1TR001881, and the National Institute of Diabetes and Digestive and Kidney Disease Diabetes Research Center (DRC) grant DK063491 to the Southern California Diabetes Endocrinology Research Center. MESA Family is conducted and supported by the National Heart, Lung, and Blood Institute (NHLBI) in collaboration with MESA investigators. Support is provided by grants and contracts R01HL071051, R01HL071205, R01HL071250, R01HL071251, R01HL071258, R01HL071259, and by the National Center for Research Resources, Grant UL1RR033176. The provision of genotyping data was supported in part by the National Center for Advancing Translational Sciences, CTSI grant UL1TR001881, and the National Institute of Diabetes and Digestive and Kidney Disease Diabetes Research Center (DRC) grant DK063491 to the Southern California Diabetes Endocrinology Research Center. Funding for SHARe genotyping was provided by NHLBI Contract N02-HL-64278. Genotyping was performed at Affymetrix (Santa Clara, California, USA) and the Broad Institute of Harvard and MIT (Boston, Massachusetts, USA) using the Affymetrix Genome-Wide Human SNP Array 6.0

Supplementary Note 2

Study Specific Acknowledgments: COPD-enriched studies

Boston Early-Onset COPD Study
The Boston Early-Onset COPD Study (dbGaP accession number phs000946) was supported by the following NIH grants: R01 HL075478, U01 HL089856, and R01 HL113264.

Genetic Epidemiology of COPD (COPDGene)
The project described was supported by Award Number U01 HL089897 and Award Number U01 HL089856 from the National Heart, Lung, and Blood Institute. The content is solely the responsibility of the authors and does not necessarily represent the official views of the National Heart, Lung, and Blood Institute or the National Institutes of Health.

COPD Foundation Funding
The COPDGene® project is also supported by the COPD Foundation through contributions made to an Industry Advisory Board comprised of AstraZeneca, Boehringer Ingelheim, GlaxoSmithKline, Novartis, Pfizer, Siemens and Sunovion.

COPDGene® Investigators – Core Units
Administrative Center: James D. Crapo, MD (PI); Edwin K. Silverman, MD, PhD (PI); Barry J. Make, MD; Elizabeth A. Regan, MD, PhD
Genetic Analysis Center: Terri Beaty, PhD; Ferdouse Begum, PhD; Peter J. Castaldi, MD, MSc; Michael Cho, MD; Dawn L. DeMeo, MD, MPH; Adel R. Boueiz, MD; Marilyn G. Foreman, MD, MS; Eitan Halper-Stromberg; Lystra P. Hayden, MD, MMSc; Craig P. Hersh, MD, MPH; Jacqueline Hetmanski, MS, MPH; Brian D. Hobbs, MD; John E. Hokanson, MPH, PhD; Nan Laird, PhD; Christoph Lange, PhD; Sharon M. Lutz, PhD; Merry-Lynn McDonald, PhD; Margaret M. Parker, PhD; Dandi Qiao, PhD; Elizabeth A. Regan, MD, PhD; Edwin K. Silverman, MD, PhD; Emily S. Wan, MD; Sungho Won, Ph.D.; Phuwanat Sakornsakolpat, M.D.; Dmitry Prokopenko, Ph.D.
Imaging Center: Mustafa Al Qaisi, MD; Harvey O. Coxson, PhD; Teresa Gray; MeiLan K. Han, MD, MS; Eric A. Hoffman, PhD; Stephen Humphries, PhD; Francine L. Jacobson, MD, MPH; Philip F. Judy, PhD; Ella A. Kazerooni, MD; Alex Kluiber; David A. Lynch, MB; John D. Newell, Jr., MD; Elizabeth A. Regan, MD, PhD; James C. Ross, PhD; Raul San Jose Estepar, PhD; Joyce Schroeder, MD; Jered Sieren; Douglas Stinson; Berend C. Stoel, PhD; Juerg Tschirren, PhD; Edwin Van Beek, MD, PhD; Bram van Ginneken, PhD; Eva van Rikxoort, PhD; George Washko, MD; Carla G. Wilson, MS;
PFT QA Center, Salt Lake City, UT: Robert Jensen, PhD
Data Coordinating Center and Biostatistics, *National Jewish Health, Denver, CO*: Douglas Everett, PhD; Jim Crooks, PhD; Camille Moore, PhD; Matt Strand, PhD; Carla G. Wilson, MS

Epidemiology Core, *University of Colorado Anschutz Medical Campus, Aurora, CO*: John E. Hokanson, MPH, PhD; John Hughes, PhD; Gregory Kinney, MPH, PhD; Sharon M. Lutz, PhD; Katherine Pratte, MSPH; Kendra A. Young, PhD
Mortality Adjudication Core: Surya Bhatt, MD; Jessica Bon, MD; MeiLan K. Han, MD, MS; Barry Make, MD; Carlos Martinez, MD, MS; Susan Murray, ScD; Elizabeth Regan, MD; Xavier Soler, MD; Carla G. Wilson, MS
Biomarker Core: Russell P. Bowler, MD, PhD; Katerina Kechris, PhD; Farnoush Banaei-Kashani, Ph.D

COPDGene® Investigators – Clinical Centers
Ann Arbor VA: Jeffrey L. Curtis, MD; Carlos H. Martinez, MD, MPH; Perry G. Pernicano, MD
Baylor College of Medicine, Houston, TX: Nicola Hanania, MD, MS; Philip Alapat, MD; Mustafa Atik, MD; Venkata Bandi, MD; Aladin Boriek, PhD; Kalpatha Guntupalli, MD; Elizabeth Guy, MD; Arun Nachiappan, MD; Amit Parulekar, MD;
Brigham and Women's Hospital, Boston, MA: Dawn L. DeMeo, MD, MPH; Craig Hersh, MD, MPH; Francine L. Jacobson, MD, MPH; George Washko, MD
Columbia University, New York, NY: R. Graham Barr, MD, DrPH; John Austin, MD; Belinda D'Souza, MD; Gregory D.N. Pearson, MD; Anna Rozenshtein, MD, MPH, FACR; Byron Thomashow, MD
Duke University Medical Center, Durham, NC: Neil MacIntyre, Jr., MD; H. Page McAdams, MD; Lacey Washington, MD
HealthPartners Research Institute, Minneapolis, MN: Charlene McEvoy, MD, MPH; Joseph Tashjian, MD
Johns Hopkins University, Baltimore, MD: Robert Wise, MD; Robert Brown, MD; Nadia N. Hansel, MD, MPH; Karen Horton, MD; Allison Lambert, MD, MHS; Nirupama Putcha, MD, MHS
Los Angeles Biomedical Research Institute at Harbor UCLA Medical Center, Torrance, CA: Richard Casaburi, PhD, MD; Alessandra Adami, PhD; Matthew Budoff, MD; Hans Fischer, MD; Janos Porszasz, MD, PhD; Harry Rossiter, PhD; William Stringer, MD
Michael E. DeBakey VAMC, Houston, *TX*: Amir Sharafkhaneh, MD, PhD; Charlie Lan, DO
Minneapolis VA: Christine Wendt, MD; Brian Bell, MD
Morehouse School of Medicine, Atlanta, GA: Marilyn G. Foreman, MD, MS; Eugene Berkowitz, MD, PhD; Gloria Westney, MD, MS
National Jewish Health, Denver, CO: Russell Bowler, MD, PhD; David A. Lynch, MB
Reliant Medical Group, Worcester, MA: Richard Rosiello, MD; David Pace, MD
Temple University, Philadelphia, PA: Gerard Criner, MD; David Ciccolella, MD; Francis Cordova, MD; Chandra Dass, MD; Gilbert D'Alonzo, DO; Parag Desai, MD; Michael Jacobs, PharmD; Steven Kelsen, MD, PhD; Victor Kim, MD; A. James Mamary, MD; Nathaniel Marchetti, DO; Aditi Satti, MD; Kartik Shenoy, MD; Robert M. Steiner, MD; Alex Swift, MD; Irene Swift, MD; Maria Elena Vega-Sanchez, MD
University of Alabama, Birmingham, AL: Mark Dransfield, MD; William Bailey, MD; Surya Bhatt, MD; Anand Iyer, MD; Hrudaya Nath, MD; J. Michael Wells, MD
University of California, San Diego, CA: Joe Ramsdell, MD; Paul Friedman, MD; Xavier Soler, MD, PhD; Andrew Yen, MD

University of Iowa, Iowa City, IA: Alejandro P. Comellas, MD; Karin F. Hoth, PhD; John Newell, Jr., MD; Brad Thompson, MD
University of Michigan, Ann Arbor, MI: MeiLan K. Han, MD, MS; Ella Kazerooni, MD; Carlos H. Martinez, MD, MPH
University of Minnesota, Minneapolis, MN: Joanne Billings, MD; Abbie Begnaud, MD; Tadashi Allen, MD
University of Pittsburgh, Pittsburgh, PA: Frank Sciurba, MD; Jessica Bon, MD; Divay Chandra, MD, MSc; Carl Fuhrman, MD; Joel Weissfeld, MD, MPH
University of Texas Health Science Center at San Antonio, San Antonio, TX: Antonio Anzueto, MD; Sandra Adams, MD; Diego Maselli-Caceres, MD; Mario E. Ruiz, MD

Supplementary Note 3

Study Specific Acknowledgments: Replication cohorts

The UK Biobank

This research has been conducted using the UK Biobank Resource under applications 648 and 4892. L.V. Wain holds a GSK/British Lung Foundation Chair in Respiratory Research. M. Tobin is supported by a Wellcome Trust Investigator Award (WT202849/Z/16/Z). M.D. Tobin and L.V. Wain have been supported by the Medical Research Council (MRC) (MR/N011317/1). CB holds UKRI Innovation Fellowship at Health Data Research UK (grant number MR/S003762/1). The research was partially supported by the National Institute for Health Research (NIHR) Leicester Biomedical Research Centre; the views expressed are those of the author(s) and not necessarily those of the National Health Service (NHS), the NIHR or the Department of Health.

The Hispanic Community Health Study / Study of Latinos

The authors thank the staff and participants of HCHS/SOL for their important contributions. The Hispanic Community Health Study/Study of Latinos is a collaborative study supported by contracts from the National Heart, Lung, and Blood Institute (NHLBI) to the University of North Carolina (HHSN268201300001I / N01-HC-65233), University of Miami (HHSN268201300004I / N01-HC-65234), Albert Einstein College of Medicine (HHSN268201300002I / N01-HC-65235), University of Illinois at Chicago – HHSN268201300003I / N01-HC-65236 Northwestern Univ), and San Diego State University (HHSN268201300005I / N01-HC-65237). The following Institutes/Centers/Offices have contributed to the HCHS/SOL through a transfer of funds to the NHLBI: National Institute on Minority Health and Health Disparities, National Institute on Deafness and Other Communication Disorders, National Institute of Dental and Craniofacial Research, National Institute of Diabetes and Digestive and Kidney Diseases, National Institute of Neurological Disorders and Stroke, NIH Institution-Office of Dietary Supplements. The Genetic Analysis Center at the University of Washington was supported by NHLBI and NIDCR contracts (HHSN268201300005C AM03 and MOD03).

Supplementary Note 4

Membership of the NHLBI TOPMed Consortium

Albert Einstein College of Medicine, New York, New York, 10461, USA
Robert Kaplan, Sylvia Smoller

Baylor College of Medicine, Houston, Texas, 77030, USA
Vivien Sheehan, Richard Gibbs

Beth Israel Deaconess Medical Center, Boston, Massachusetts, 02215, USA
Robert Gerszten, James G. Wilson

Blood Works Northwest, Seattle, Washington, 98102, USA
Jill Johnsen

Blood Works Northwest, Seattle, Washington, 98104, USA
Barbara Konkle

Blood Works Northwest, Seattle, Washington, 98105, USA
Haley Huston

Blood Works Northwest, Seattle, Washington, 98107, USA
Sarah Ruuska

Boston University, Boston, Massachusetts, 02118, USA
Emelia Benjamin, L. Adrienne Cupples

Boston University, Boston, Massachusetts, 02215, USA
Honghuang Lin, Kathryn Lunetta, Vasan S. Ramachandran

Brigham & Women's Hospital, Boston, Massachusetts, 02111, USA
Peter Castaldi

Brigham & Women's Hospital, Boston, Massachusetts, 02114, USA
Christine Albert

Brigham & Women's Hospital, Boston, Massachusetts, 02115, USA
Brian Cade, Vincent Carey, Michael Cho, Dawn DeMeo, Adel El Boueiz, Auyon Ghosh, Craig Hersh, Brian Hobbs, Wonji Kim, Jessica Lasky-Su, Meryl LeBoff, Jiwon Lee, JoAnn Manson, Matt Moll, Dandi Qiao, Susan Redline, Edwin Silverman, Tamar Sofer, Jody Sylvia, Heming Wang, Scott Weiss, Carla Wilson

Brigham & Women's Hospital, Boston, Massachusetts, 02215, USA
Daniel Chasman

Broad Institute, Cambridge, Massachusetts, 02142, USA
Francois Aguet, Kristin Ardlie, Mark Chaffin, Seung Hoan Choi, Clary Clish, Stacey Gabriel, Namrata Gupta, Sekar Kathiresan, Lauren Margolin, Carolina Roselli, Maryam Zekavat

Broad Institute, Harvard University, Boston, Massachusetts, 02115, USA
Vijay G. Sankaran

Broad Institute, Harvard University, Cambridge, Massachusetts, 02138, USA
Pradeep Natarajan

Brown University, Providence, Rhode Island, 02912, USA
Charles Eaton, Simin Liu, Stephen McGarvey

Case Western Reserve University, Cleveland, Ohio, 44106, USA
Xiaofeng Zhu

Children's Hospital of Philadelphia, University of Pennsylvania, Philadelphia, Pennsylvania, 19104, USA
Laura Almasy

Cleveland Clinic, Cleveland, Ohio, 44195, USA
John Barnard, Gerald Beck, Mina Chung, Suzy Comhair, Serpil Erzurum

Columbia University, New York, New York, 10032, USA
R. Graham Barr, Danish Saleheen

Duke University, Durham, North Carolina, 27701, USA
Yongmei Liu

Duke University, Durham, North Carolina, 27708, USA
Allison Ashley-Koch, Marilyn Telen

Emory University, Atlanta, Georgia, 30322, USA
Alvaro Alonso, Rich Johnston, Lawrence S. Phillips, Stephanie L. Sherman, Zhaohui Qin

Fred Hutchinson Cancer Research Center, Seattle, Washington, 98109, USA
Margery Gass, Jeff Haessler, Charles Kooperberg, Lesley Tinker, Ulrike Peters, Alex Reiner

Fundação de Hematologia e Hemoterapia de Pernambuco – Hemope, Recife, 52011-000, Brazil
Marcos Bezerra

George Washington University, Washington, District of Columbia, 20052, USA
Lisa Martin

Harvard Medical School, Boston, Massachusetts, 02115, USA
Christine Seidman, Jonathan Seidman

Harvard School of Public Health, Boston, Massachusetts, 02115, USA
Christoph Lange, Xihong Lin

Harvard University, Cambridge, Massachusetts, 02138, USA
Sean McFarland

Henry Ford Health System, Detroit, Michigan, 48202, USA
L. Keoki Williams

Howard University, Washington, District of Columbia, 20059, USA
Sergei Nekhai

Icahn School of Medicine at Mount Sinai, New York, New York, 10029, USA
Bruce Gelb, Eimear Kenny, Ruth J. F. Loos, Arden Moscati, Girish Nadkarni, Michael Preuss

Indiana University, Indianapolis, Indiana, 46202, USA
Mark Geraci, Jennifer Wessel

Johns Hopkins University, Baltimore, Maryland, 21218, USA
Dan Arking, Dimitrios Avramopoulos, Emily Barron-Casella, Terri Beaty, Diane Becker, Lewis Becker, James Casella, Kimberly Jones, Barry Make, Rasika Mathias, Rakhi Naik, Wendy Post, Ingo Ruczinski, Steven Salzberg, Margaret Taub, Dhananjay Vaidya, Lisa Yanek

Loyola University, Maywood, Illinois, 60153, USA
Holly Kramer

Lundquist Institute, Charlottesville, Virginia, 90502, USA
Yii-Der Ida Chen, Xiuqing Guo, Xiaohui Li, Henry Lin, Jerome Rotter, Kevin Sandow, Kent D. Taylor

Lutia I Puava Ae Mapu I Fagalele, Apia, Samoa
Muagututia Sefuiva Reupena

Massachusetts General Hospital, Boston, Massachusetts, 02114, USA
Patrick Ellinor, Steven Lubitz, Lu-Chen Weng

Mayo Clinic, Rochester, Minnesota, 55905, USA
Mariza de Andrade

McGill University, Montreal, Quebec, H3A 0G4, Canada
Ryan Hernandez

Medical College of Wisconsin, Milwaukee, Wisconsin, 53226, USA
Ulrich Broeckel

Ministry of Health, Government of Samoa, Apia, Samoa
Take Naseri

National Health Research Institute Taiwan, Miaoli County, 350, Taiwan
Ren-Hua Chung, Chao (Agnes) Hsiung

National Heart, Lung, and Blood Institute, National Institutes of Health, Bethesda, Maryland, 20892, USA
Deborah Applebaum-Bowden, Rebecca Beer, Weiniu Gan, Cashell Jaquish, Andrew Johnson, Dan Levy, James Luo, Julie Mikulla, Mollie Minear, George Papanicolaou, Pankaj Qasba

National Jewish Health, Denver, Colorado, 80206, USA
Russell Bowler, James Crapo, Tasha Fingerlin, Elizabeth Regan, Snow Xueyan Zhao

National Taiwan University, Taipei, 10617, Taiwan
Yi-Cheng Chang, Lee-Ming Chuang

New York Genome Center, New York, New York, 10013, USA
Namiko Abe, Karen Bunting, Bo-Juen Chen, Soren Germer, Tanja Smith, Michael Zody

Northwestern University, Chicago, Illinois, 60208, USA
Laura Rasmussen-Torvik

Ohio State University Wexner Medical Center, Columbus, Ohio, 43210, USA
Rebecca Jackson

Oklahoma Medical Research Foundation, Oklahoma City, Oklahoma, 73104, USA
Courtney Montgomery

Stanford University, Stanford, California, 94304, USA
David Paik

Stanford University, Stanford, California, 94305, USA
Tim Assimes, Carlos Bustamante, Chris Gignoux, Yu Liu, Marco Perez, Michael Snyder, Hua Tang, Joseph Wu

Taichung Veterans General Hospital Taiwan, Taichung City, 407, Taiwan
Chii Min Hwu, Wen-Jane Lee, Wayne Hui-Heng Sheu

The Emmes Corporation, Rockville, Maryland, 20850, USA
Lucas Barwick

Tri-Service General Hospital National Defense Medical Center, Taipei, 114, Taiwan
Yi-Jen Hung

Tulane University, New Orleans, Louisiana, 70118, USA
Jiang He

UMass Memorial Medical Center, Worcester, Massachusetts, 1655, USA
Brian Silver

Universidade de Sao Paulo, Sao Paulo, , 01310000, Brazil
Ester Cerdeira Sabino

University at Buffalo, Buffalo, New York, 14260, USA
Heather Ochs-Balcom

University of Alabama, Birmingham, Alabama, 35294, USA
Hemant Tiwari

University of Alabama, Birmingham, Alabama, 35487, USA
Stella Aslibekyan, Bertha Hidalgo, Marguerite Ryan Irvin, Merry-Lynn McDonald

University of Arizona, Tucson, Arizona, 85721, USA
Deborah A. Meyers

University of California, Los Angeles, Los Angeles, California, 90095, USA
Richard Casaburi, Carolyn Crandall, Karol Watson

University of California, San Francisco, San Francisco, California, 94143, USA
Esteban Burchard

University of Chicago, Chicago, Illinois, 60637, USA
Sean David

University of Cincinnati, Cincinnati, Ohio, 45220, USA
Ranjan Deka

University of Colorado Anschutz Medical Campus, Aurora, Colorado, 80045, USA
Kathleen Barnes, Sharon Graw, Luisa Mestroni, Matthew Taylor

University of Colorado at Boulder, University of Minnesota, Boulder, Colorado, 80309, USA
Scott Vrieze

University of Colorado at Denver, Denver, Colorado, 80045, USA
Nicholas Rafaels

University of Colorado at Denver, Denver, Colorado, 80204, USA
Jonathan Cardwell, Sameer Chavan, Michelle Daya, Shanshan Gao, Daniel Grine, John Hokanson, Greg Kinney, Ethan Lange, Leslie Lange, Susan Mathai, Bonnie Neltner, Julia Powers Becker, Meher Preethi Boorgula, Pamela Russell, David Schwartz, Aniket Shetty, Garrett Storm, Tarik Walker, Avram Walts, Ivana Yang

University of Illinois at Chicago, Chicago, Illinois, 60607, USA
Dawood Darbar

University of Iowa, Iowa City, Iowa, 52242, USA
Karin Hoth, Robert Wallace

University of Kentucky, Lexington, Kentucky, 40506, USA
Donna K Arnett

University of Maryland, Baltimore, Maryland, 21201, USA
Seth Ament, Amber Beitelshees, Christy Chang, Coleen Damcott, Scott Devine, Mao Fu, Da-Wei Gong, Yue Guan, Daniel Harris, Elliott Hong, Michael Kessler, Joshua Lewis, Patrick McArdle, Braxton D. Mitchell, May E. Montasser, Jeff O'Connell, Tim O'Connor, Afshin Parsa, James Perry, Toni Pollin, Robert Reed, Kathleen Ryan, Shabnam Salimi, Amol Shetty, Elizabeth Streeten, Carole Sztalryd, Simeon Taylor, Huichun Xu, Rongze Yang, Norann Zaghloul

University of Michigan, Ann Arbor, Michigan, 48109, USA
Goncalo Abecasis, Larry Bielak, Thomas Blackwell, Jeffrey Curtis, Sayantan Das, Matthew Flickinger, Xiaoqi (Priscilla) Geng, Min A Jhun, Hyun Min Kang, Sharon Kardia, Seunggeun Shawn Lee, Jonathon LeFaive, Keng Han Lin, Patricia Peyser, Christopher Scheller, Ellen Schmidt, Jennifer Smith, Daniel Taliun, Peter VandeHaar, Cristen Willer, Wei Zhao, Xiang Zhou, Sebastian Zoellner

University of Minnesota, Minneapolis, Minnesota, 55455, USA
James Pankow, Michael Tsai

University of Mississippi, Jackson, Mississippi, 38677, USA
Pramod Anugu, Lynette Ekunwe, Yan Gao, Michael Hall, Hao Mei, Nancy Min, Stanford Mwasongwe

University of Mississippi, Jackson, Mississippi, 39213, USA
Solomon Musani

University of Mississippi, Jackson, Mississippi, 39216, USA
Adolfo Correa

University of North Carolina, Chapel Hill, North Carolina, 27599, USA
Qing Duan, Nora Franceschini, Yun Li, Kari North, Laura Raffield,

University of Pennsylvania, Philadelphia, Pennsylvania, 19104, USA
Sarah Tishkoff

University of Pittsburgh, Pittsburgh, Pennsylvania, 15206, USA
Takis Benos

University of Pittsburgh, Pittsburgh, Pennsylvania, 15260, USA
Mark Gladwin, RyanL Minster, Frank Sciurba, Daniel E. Weeks, Yingze Zhang

University of Southern California, California, 90033, USA
David Van Den Berg

University of Texas Health at Houston, Houston, Texas, 77225, USA
Eric Boerwinkle, Myriam Fornage, James Hixson, Degui Zhi

University of Texas Rio Grande Valley School of Medicine, Brownsville, Texas, 78520, USA
John Blangero, Joanne Curran, Michael Mahaney

University of Texas Rio Grande Valley School of Medicine, Edinburg, Texas, 78539, USA
Ravi Duggirala, Juan Manuel Peralta

University of Texas Rio Grande Valley School of Medicine, San Antonio, Texas, 78229, USA
Harald Goring

University of Vermont, Burlington, Vermont, 05405, USA
Elaine Cornell, Jon Peter Durda, Russell Tracy

University of Virginia, Charlottesville, Virginia, 22908, USA
Wei-Min Chen, Charles Farber, Ani Manichaikul, Josyf C Mychaleckyj, Aakrosh Ratan, Stephen Rich

University of Washington, Seattle, Washington, 98101, USA
Joshua Bis

University of Washington, Seattle, Washington, 98105, USA
Catherine Tong

University of Washington, Seattle, Washington, 98115, USA
Matthew Conomos

University of Washington, Seattle, Washington, 98145, USA
Caitlin McHugh

University of Washington, Seattle, Washington, 98195, USA
Peter Anderson, Jennifer Brody, Jai Broome, Erin Buth, Colleen Davis, Leslie Emery, Chris Frazar, Stephanie M. Fullerton, Stephanie Gogarten, Ben Heavner, Susan Heckbert, Deepti Jain, Craig Johnson, Alyna Khan, Cathy Laurie, Cecelia Laurie, David Levine, Susanne May, Daniel McGoldrick, Sarah C. Nelson, Deborah Nickerson, Bruce Psaty, Ken Rice, Josh Smith, Nicholas Smith, Nona Sotoodehnia, Adrienne M. Stilp, Adam Szpiro, Timothy A. Thornton, Machiko Threlkeld, David Tirschwell, Fei Fei Wang, Bruce Weir, Kayleen Williams, Quenna Wong

University of Wisconsin Milwaukee, Milwaukee, Wisconsin, 53211, USA
Paul Auer

Vanderbilt University, Nashville, Tennessee, 37212, USA
M. Benjamin Shoemaker

Vanderbilt University, Nashville, Tennessee, 37235, USA
Michael DeBaun, Dan Roden

Vitalant Research Institute, San Francisco, California, 94118, USA
Brian Custer, Shannon Kelly

Wake Forest Baptist Health, Winston-Salem, North Carolina, 27157, USA
Donald W. Bowden, David Herrington, Nicholette Palmer, Beverly Snively

Washington State University, Seattle, Washington, 98109, USA
Cara Carty

Washington University in St Louis, St Louis, Missouri, 63130, USA
Susan Dutcher, Lucinda Fulton, C. Charles Gu, D.C. Rao, Karen Schwander, Yun Ju Sung, Lisa de las Fuentes

Yale University, New Haven, Connecticut, 06520, USA
David Glahn, NicolaL. Hawley

Supplementary Note 5

Membership of the TOPMed Lung Working Group

Albert Einstein College of Medicine, New York, New York, 10461, USA
Robert Kaplan, Simon Spivack

Beth Israel Deaconess Medical Center, Boston, Massachusetts, 02215, USA
Debby Ngo

Boston University School of Public Health, Boston, Massachusetts, 02118, USA
Josée Dupuis, Hanfei Xu

Boston University, Boston, Massachusetts, 02118, USA
George O'Connor

Brigham & Women's Hospital, Boston, Massachusetts, 02111, USA
Peter Castaldi,

Brigham & Women's Hospital, Boston, Massachusetts, 02115, USA
Brian Cade, Michael Cho, Damien Croteau-Chonka, Dawn DeMeo, Huawei Dong, Adel El Boueiz, Lystra Hayden, Craig Hersh, Brian Hobbs, Priyadarshini Kachroo, Wonji Kim, Jessica Lasky-Su, Jiwon Lee, Margaret Parker, John Platig, Dandi Qiao, Susan Redline, Phuwanat Sakornsakolpat, Minseok Seo, Edwin Silverman, Emily Wan, Scott Weiss,

Children's Hospital of Philadelphia, Philadephia, Pennsylvania, 19104, USA
Hakon Hakonarson, Patrick Sleiman

Cleveland Clinic, Cleveland, Ohio, 44195, USA
John Barnard, Gerald Beck, Suzy Comhair, Serpil Erzurum, Joe Zein

Columbia University, New York, New York, 10032, USA
Pallavi Balte, R. Graham Barr, Kristina Buschur, Christine Kim Garcia, David Lederer, Purnema Madahar, Benjamin Smith

Harvard Medical School, Boston, Massachusetts, 02115, USA
Paul Avillach

Harvard Medical School, Boston, Massachusetts, 02215, USA
Sharon Lutz

Harvard School of Public Health, Boston, Massachusetts, 02115, USA
David C. Christiani, Mulong Du, Sheila Gaynor, Divy Kangeyan, Christoph Lange, Xihong Lin

Harvard University, Cambridge, Massachusetts, 02138, USA
Dmitry Prokopenko

Henry Ford Health System, Detroit, Michigan, 48202, USA
Hongsheng Gui, Keoki Williams, Shujie Xiao

Icahn School of Medicine at Mount Sinai, New York, New York, 10029, USA
Eimear Kenny, Ruth J.F. Loos

Indiana University, Indianapolis, Indiana, 46202, USA
Micheala Aldred, Mark Geraci

Johns Hopkins Bloomberg School of Public Health, Baltimore, Maryland, 21205, USA
Priya Duggal, Woori Kim, Ingo Ruczinski

Johns Hopkins University, Baltimore, Maryland, 21218, USA
Barry Make

Lundquist Institute, Torrance, California, 90502, USA
Kent D. Taylor

Lurie Children's Hospital of Chicago, Chicago, Illinois, 60611, USA
Rajesh Kumar

Morehouse School of Medicine, Atlanta, Georgia, 30319, USA
Marilyn Foreman

National Health Research Institute Taiwan, Miaoli County, 350, Taiwan
Chao (Agnes) Hsiung

National Heart, Lung, and Blood Institute, National Institutes of Health, Bethesda, Maryland, 20892, USA
Weiniu Gan, Julie Mikulla

National Institute of Environmental Health Sciences, Durham, North Carolina, 27709, USA
Stephanie London

National Jewish Health, Denver, Colorado, 80206, USA
Russell Bowler, Tasha Fingerlin, Lucas Gillenwater

New York Genome Center, New York, New York, 10013, USA
Silva Kasela, Tuuli Lappalainen, Michael Zody

Northwestern University, Chicago, Illinois, 60611, USA
Lifang Hou, Ravi Kalhan, Wei Zhang, Yinan Zheng

Oklahoma Medical Research Foundation, Oklahoma City, Oklahoma, 73104, USA
Courtney Montgomery

Tougaloo College, Tougaloo, Mississippi, 39174, USA
Wendy White

University of Alabama, Birmingham, Alabama, 35294, USA
Surya Bhatt, Preeti Lakshman Kumar, Merry-Lynn McDonald

University of Arizona, Tucson, Arizona, 85721, USA
Eugene Bleecker, Xingnan Li, Deborah A Meyers

University of California, Los Angeles, Los Angeles, Califonia, 90095, USA
Richard Casaburi

University of California, San Francisco, San Francisco, Califonia, 94143, USA
Esteban Burchard, Angel Mak, Marquitta White

University of Chicago, Chicago, Illinois, 60637, USA
Hae Kyung Im

University of Colorado Anschutz Medical Campus, Aurora, Colorado, 80045, USA
Kathleen Barnes, Katerina Kechris

University of Colorado at Denver, Denver, Colorado, 80204, USA
Michelle Daya, Leslie Lange, Susan Mathai, David Schwartz, Marvin Schwarz, Tarik Walker, Ivana Yang, Kendra Young

University of Maryland School of Medicine, Baltimore, Maryland, 21201, USA
Robert Reed

University of Michigan, Ann Arbor, Michigan, 48109, USA
Xutong Zhao

University of Mississippi, Jackson, Mississippi, 39216, USA
Adolfo Correa

University of North Carolina, Chapel Hill, North Carolina, 27516, USA
Danyu Lin

University of North Carolina, Chapel Hill, North Carolina, 27599, USA
Kari North

University of Pennsylvania, Philadephia, Pennsylvania, 19104, USA
Blanca Himes

University of Pittsburgh, Pittsburgh, Pennsylvania, 15206, USA
Takis Benos

University of Pittsburgh, Pittsburgh, Pennsylvania, 15260, USA
Frank Sciurba

University of Southern California, Los Angeles, Califonia, 90032, USA
Frank Gilliland, Linda Polfus

University of Texas Health at Houston, Houston, Texas, 77030, USA
Bing Yu

University of Virginia, Charlottesville, Virginia, 22908, USA
Xiaowei Hu, Ani Manichaikul, Jennifer Nguyen, Stephen Rich, Chaojie Yang

University of Washington, Seattle, Washington, 98104, USA
Mark Wurfel

University of Washington, Seattle, Washington, 98109, USA
Sina Gharib

University of Washington, Seattle, Washington, 98195, USA
David Beame, Michael Bowers, Matthew Conomos, Addison Keely, Jennifer Anne Purnell, Margaret F. Ragland, Timothy A. Thornton, Kate Wehr

Wake Forest Baptist Health, Winston-Salem, North Carolina, 27157, USA
Victor Ortega

Washington University in St. Louis, St. Louis, Missouri, 63130, USA
Susan Dutcher

Supplementary Table 1: Genomic inflation factors for single variant WGS analyses.

Stratum	Race/ethnic group	FEV1	FVC	FEV1/FVC	Moderate-severe COPD	Severe COPD
Population- and family-based	White	1.028	1.028	1.028	1.011	1.023
	African American	1.041	1.033	1.029	1.058	0.892
	All	0.972	0.981	1.003	1.029	1.081
COPD-enriched	White	1.027	1.040	0.955	1.012	1.042
	African American	1.001	1.012	1.007	1.008	1.031
	All	0.851	0.945	0.758	1.008	0.992
Combined	White	1.029	1.029	1.019	1.019	1.044
	African American	1.037	1.031	1.026	0.995	1.029
	All	0.846	0.979	0.938	1.019	1.018

Supplementary Table 2: Association of measured methylation with FEV_1 in MESA.

Trait	Race/ethnic group	mProbe	Sample size	Beta	Std. Error	P-value	95% CI lower	95% CI upper
FEV1	Non-Hispanic White	cg06249499	235	0.0235	0.0663	0.7233	-0.1065	0.1535
FEV1	African American	cg06249499	123	0.2691	0.0961	0.0061	0.0808	0.4574
FEV1	Hispanic	cg06249499	210	0.0655	0.0664	0.3254	-0.0647	0.1958
FEV1	Chinese	cg06249499	58	-0.0824	0.1462	0.5766	-0.3689	0.2041
FEV1	Multi-ethnic	cg06249499	626	0.1012	0.0378	0.0076	0.0272	0.1752

Results are shown based on linear regression of lung function traits on methylation levels with covariate adjustment for age, age-squared, sex, height, height^2, weight, current smoking, former smoking, pack-years of smoking, PCs of ancestry, and methylation PEER factors. Regression output includes the regression coefficients (beta), standard error, *P*-values from using the two-sided t-test, and the corresponding 95% confidence limits derived by inverting the t-test

Supplementary Table 3: Mendelian candidate genes for pLOF analysis.

Gene	Chr	Start position (b38)	End position (b38)	Number of pLOF variants included in the gene burden in the dataset
ALDH18A1	10	95,605,929	95,656,901	9
ATP6V0A2	12	123,712,318	123,761,755	32
ATP6V1A	3	113,747,019	113,812,058	2
ATP6V1E1	22	17,592,136	17,628,822	1
CFTR	7	117,479,963	117,668,665	68
COL3A1	2	188,974,373	189,012,746	2
EFEMP2	11	65,866,441	65,872,934	10
ELN	7	74,027,772	74,069,907	18
FBLN1	22	45,502,839	45,601,134	19
FBLN5	14	91,869,411	91,947,702	4
FLCN	17	17,206,946	17,237,191	10
GORAB	1	170,532,122	170,553,834	15
LTBP4	19	40,592,888	40,629,820	33
NAF1	4	163,109,133	163,166,921	4
NPC2	14	74,479,940	74,493,381	15
PARN	16	14,435,700	14,630,286	13
PYCR1	17	81,932,384	81,937,328	13
RIN2	20	19,757,610	20,002,459	17
RTEL1	20	63,657,810	63,696,253	27
SERPINA1	14	94,376,747	94,390,692	10
SLC17A5	6	73,593,379	73,654,014	19
SLC2A10	20	46,708,358	46,736,347	11
SMPD1	11	6,390,414	6,394,998	22
TERC	3	169,764,610	169,765,060	0
TERT	5	1,253,167	1,295,047	6
TINF2	14	24,239,641	24,242,674	13

Supplementary Figure 1: Power of quantitative trait analysis in discovery samples from TOPMed. Power is shown across a range of values for the genetic additive effect in units of trait standard deviations, as indicated on the figure legends. The range of sample sizes reflect analysis in the full sample (n=20,000) as well as multiple smaller values reflecting stratified analyses in race/ethnic- or population vs. COPD-enriched subgroups. On the x-axis, "MAF" denotes minor allele frequency. Power calculations were carried out using QUANTO.[30] Supplementary Figure 1a presents power assuming a nominal significance threshold of *P*=0.05, while Supplementary Figure 1b shows power for genome-wide analysis, assuming a significance threshold of $P=5x10^{-8}$.

a) Power assuming a nominal significance threshold of *P*=0.05.

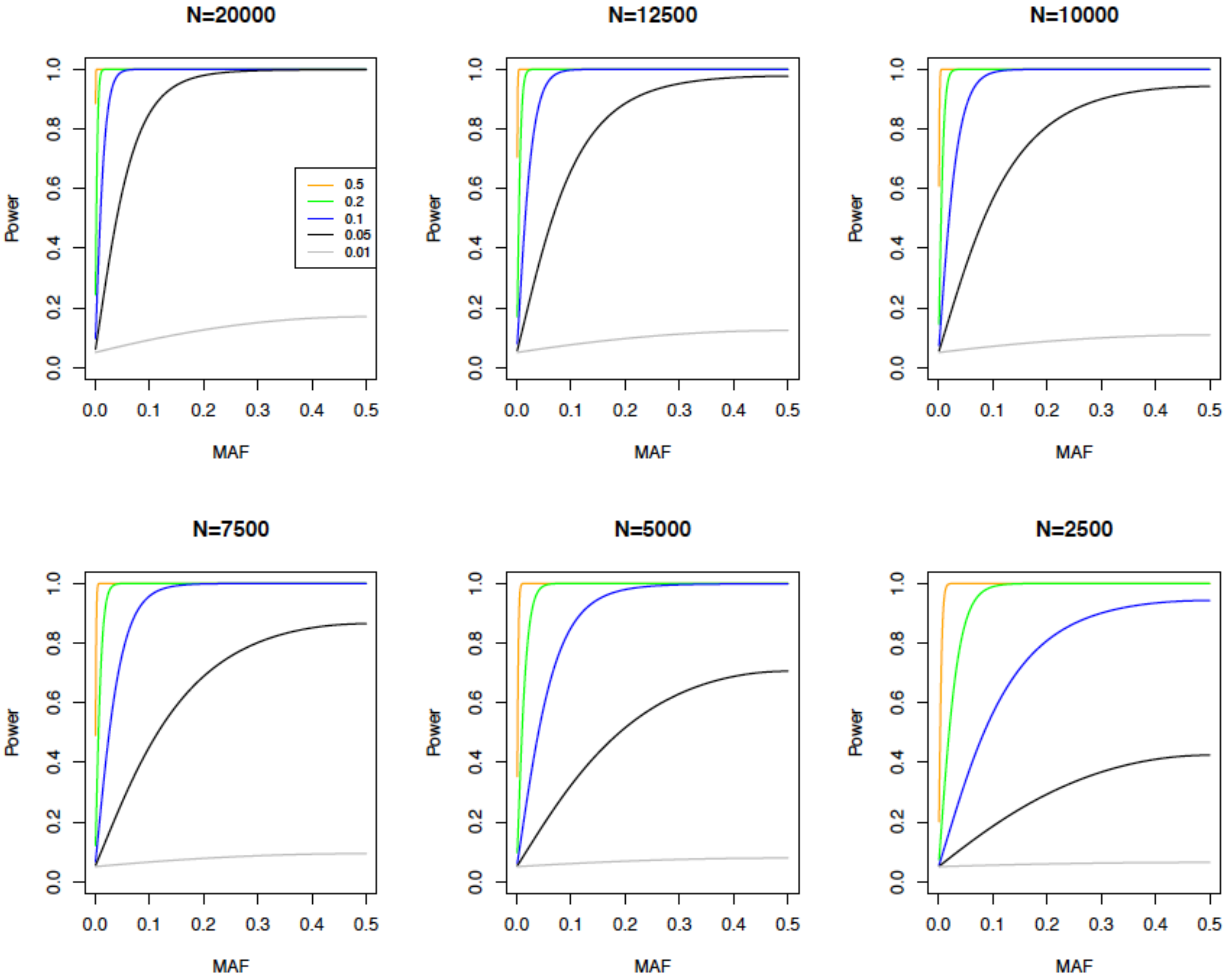

b) Power for genome-wide analysis, assuming a significance threshold of P=5x10^{-8}.

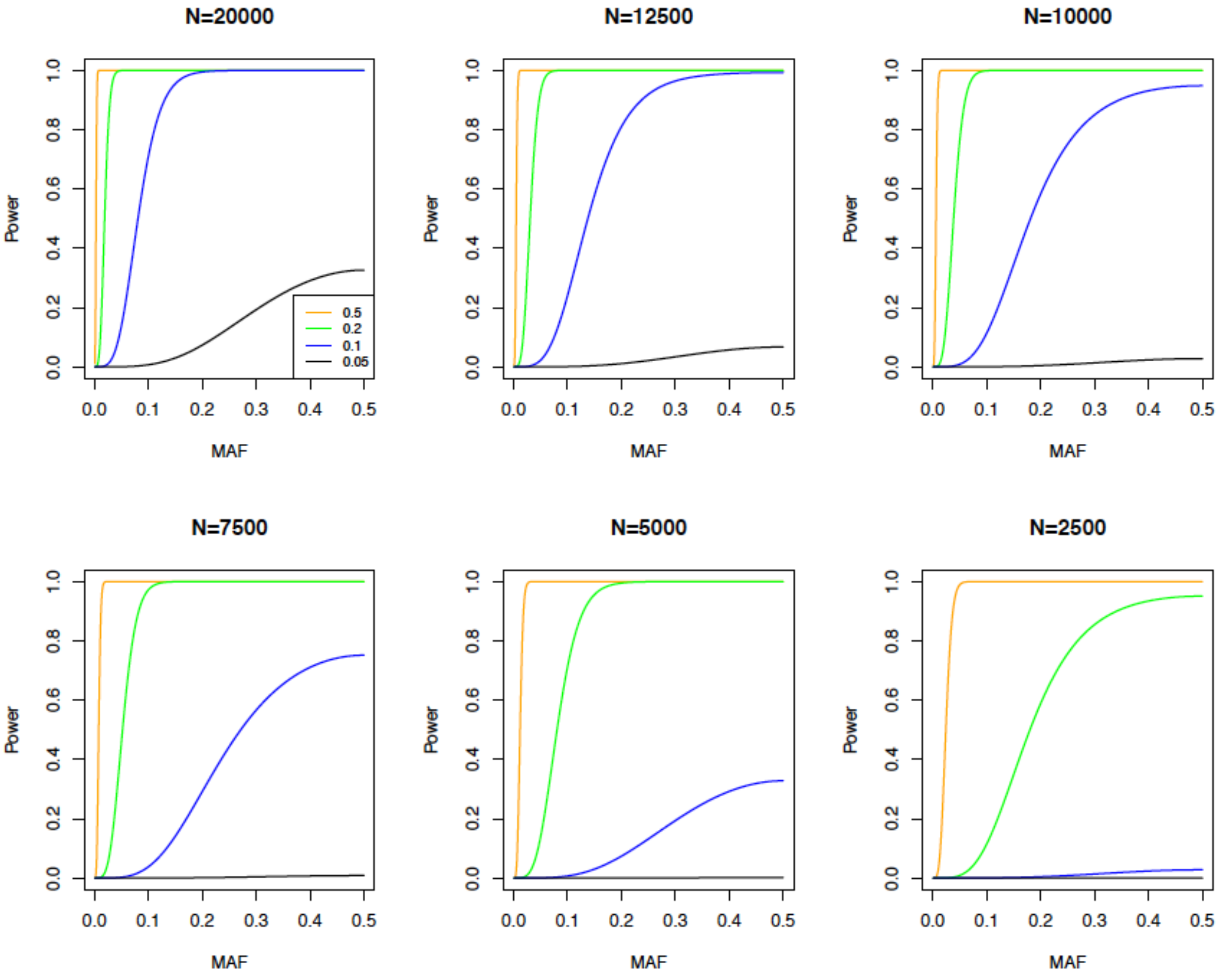

Supplementary Figure 2: Local association plots including the +/- 250 kb flank region for novel variants displayed in Supplementary Data 2. Supplementary Figures 2a-aa present the local association plots corresponding to each of the 27 genome-wide significant results at novel loci, in the same order as presented in Supplementary Data 2. We constructed three reference panels for linkage disequilibrium (LD) using the subsets of African American, White or combined race/ethnic TOPMed samples included in our PFT/COPD WGS analyses. For each plot, we applied the custom LD reference panel corresponding to the race/ethnic group in which the analysis was performed.

a) **FEV_1:** Population- and family-based, African American; rs75195732 (chr17: 69,937,351)

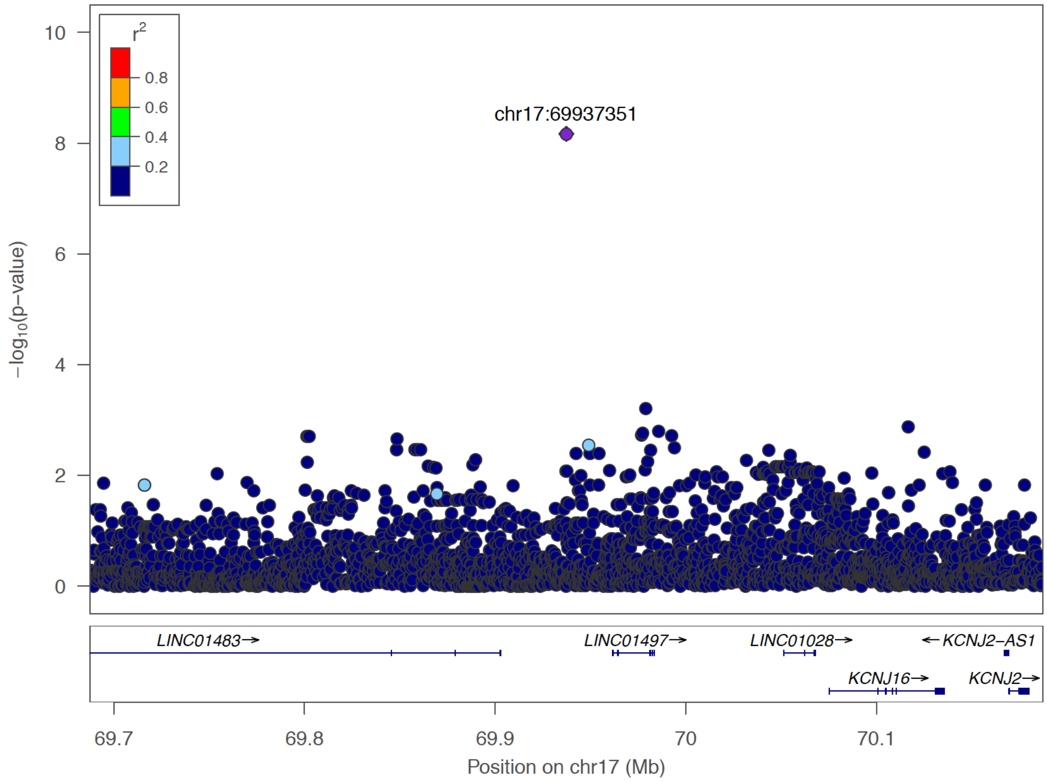

b) **FEV$_1$:** Population- and family-based, All; rs10984916 (chr9:120,443,427)

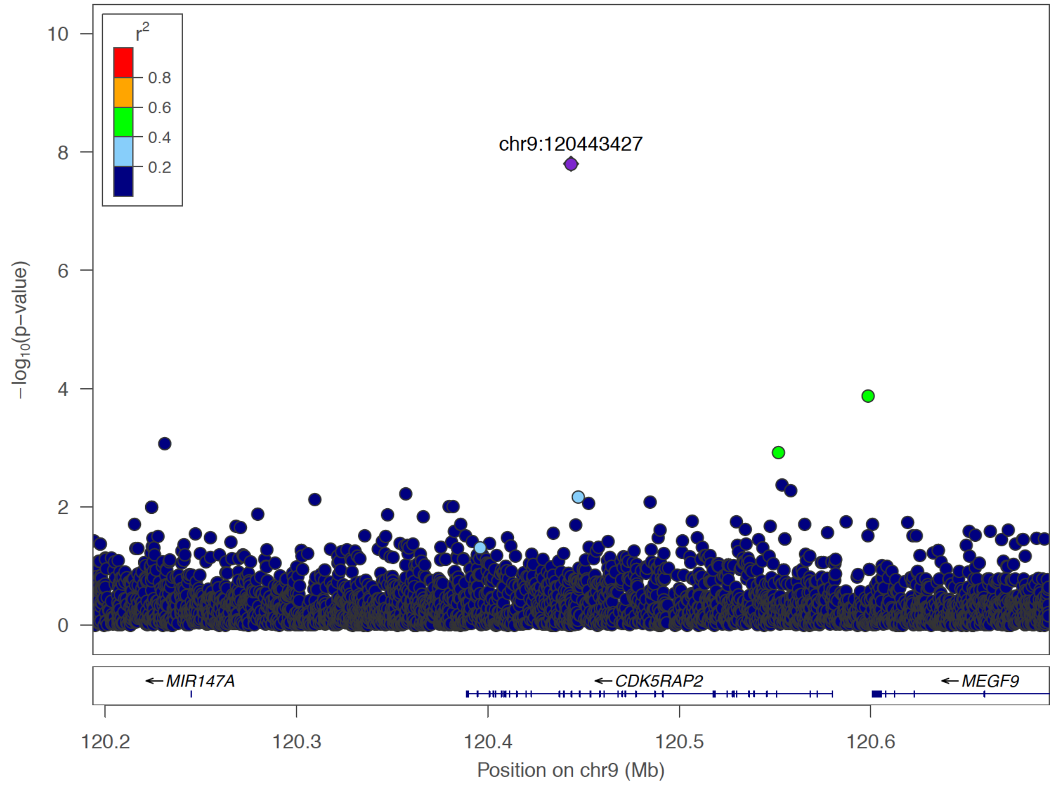

c) **FEV1:** COPD-enriched, African American; rs4076943 (chr11:11,239,853)

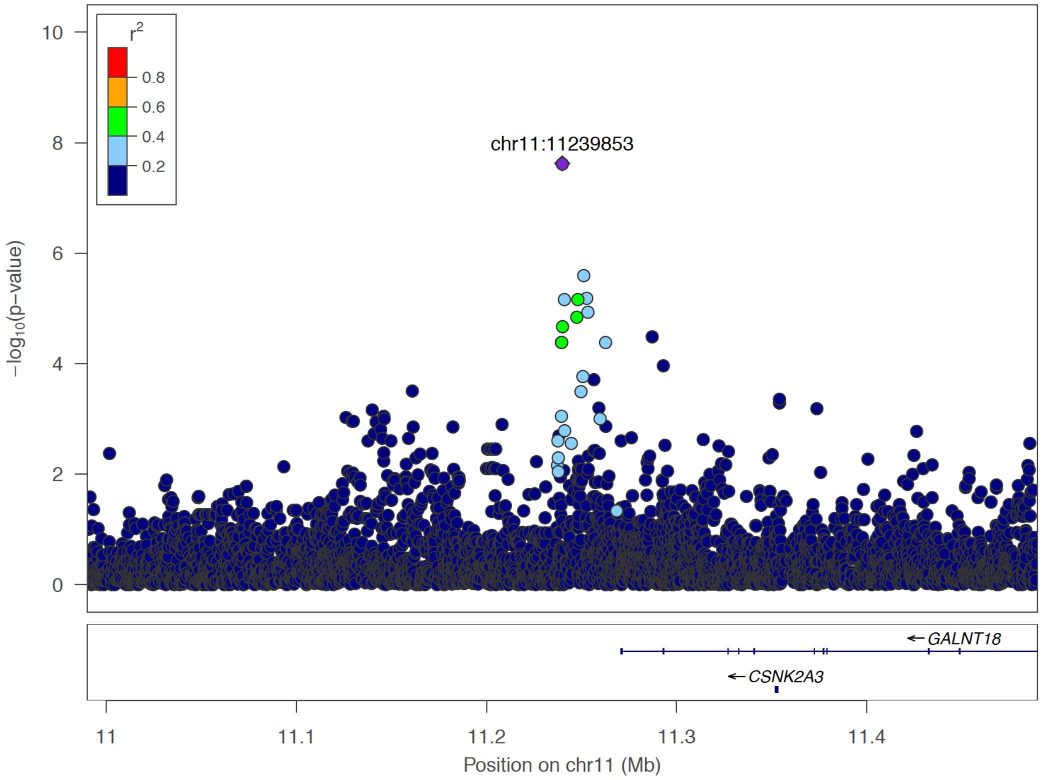

d) **FEV₁:** COPD-enriched, White; rs142755000 (chrX:80,958,253)

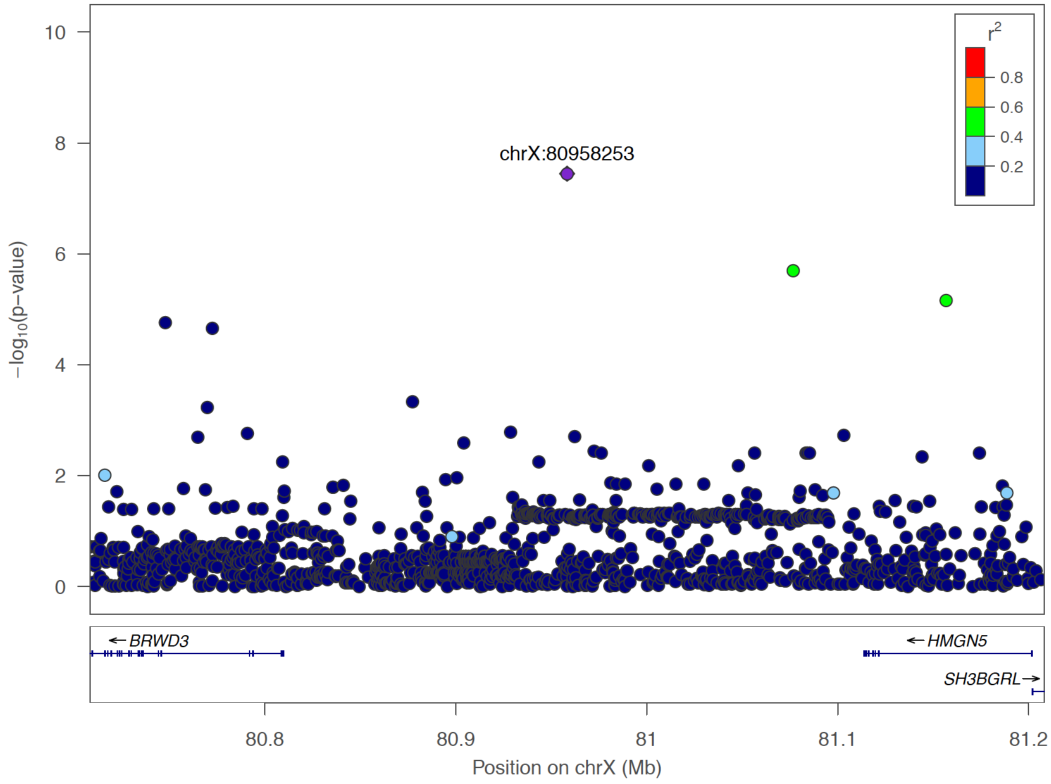

e) **FEV1:** Combined, White; rs9295345 (chr6:166,400,303)

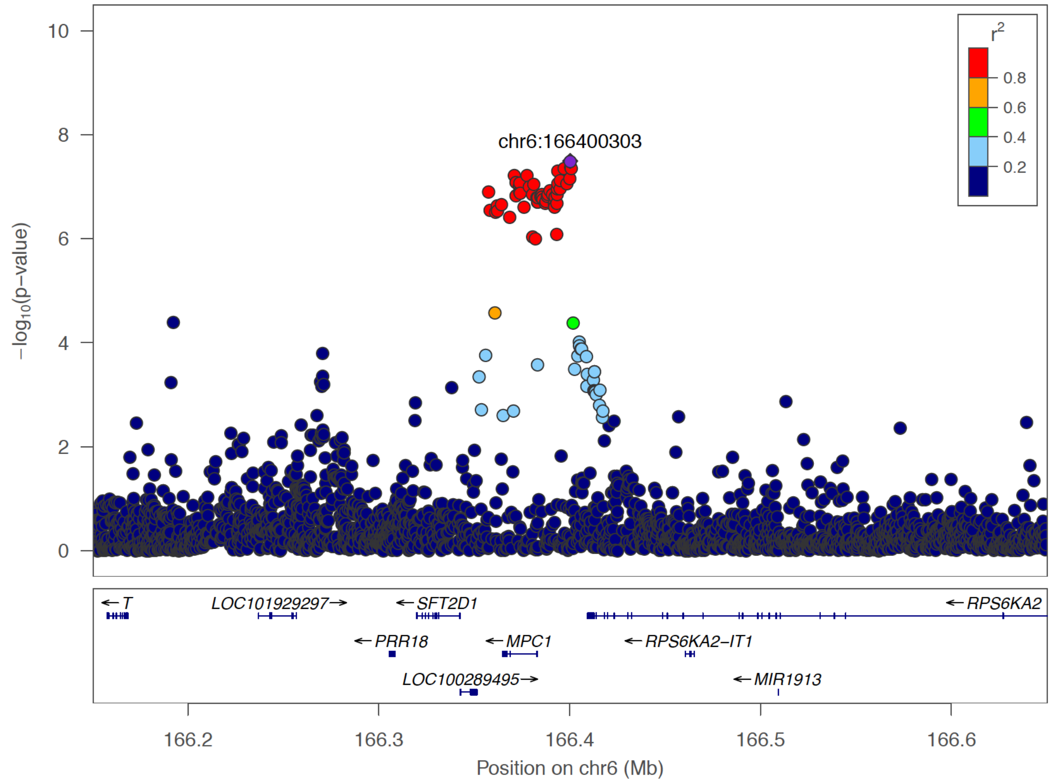

f) **FVC:** Population- and family-based, White; rs182915372 (chrX:46,687,945)

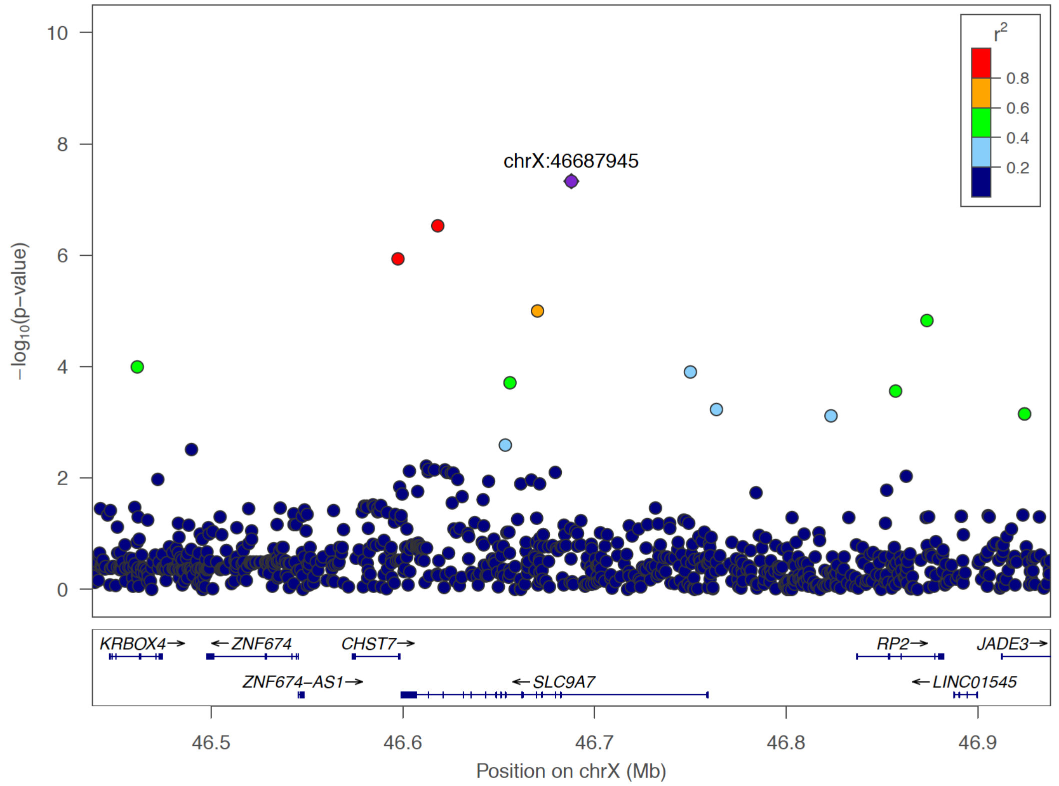

g) **FVC:** Population- and family-based, All; rs182915372 (chrX:46,687,945)

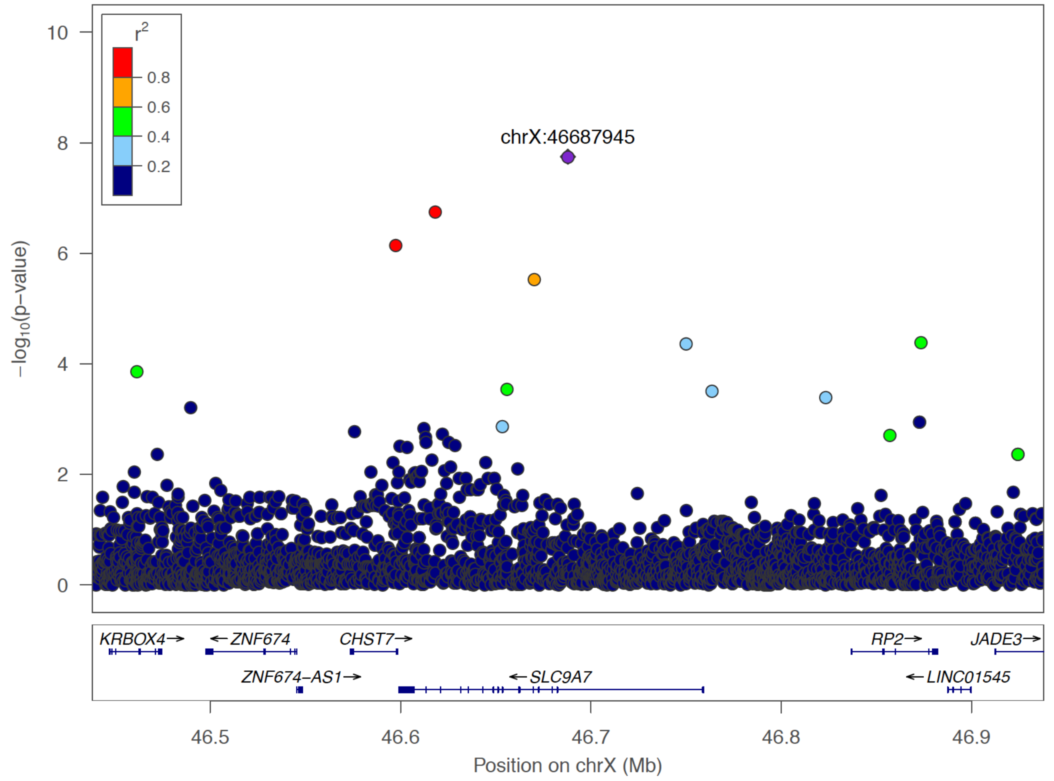

h) **FVC:** COPD-enriched, African American; rs74469188 (chr16:81,611,365)

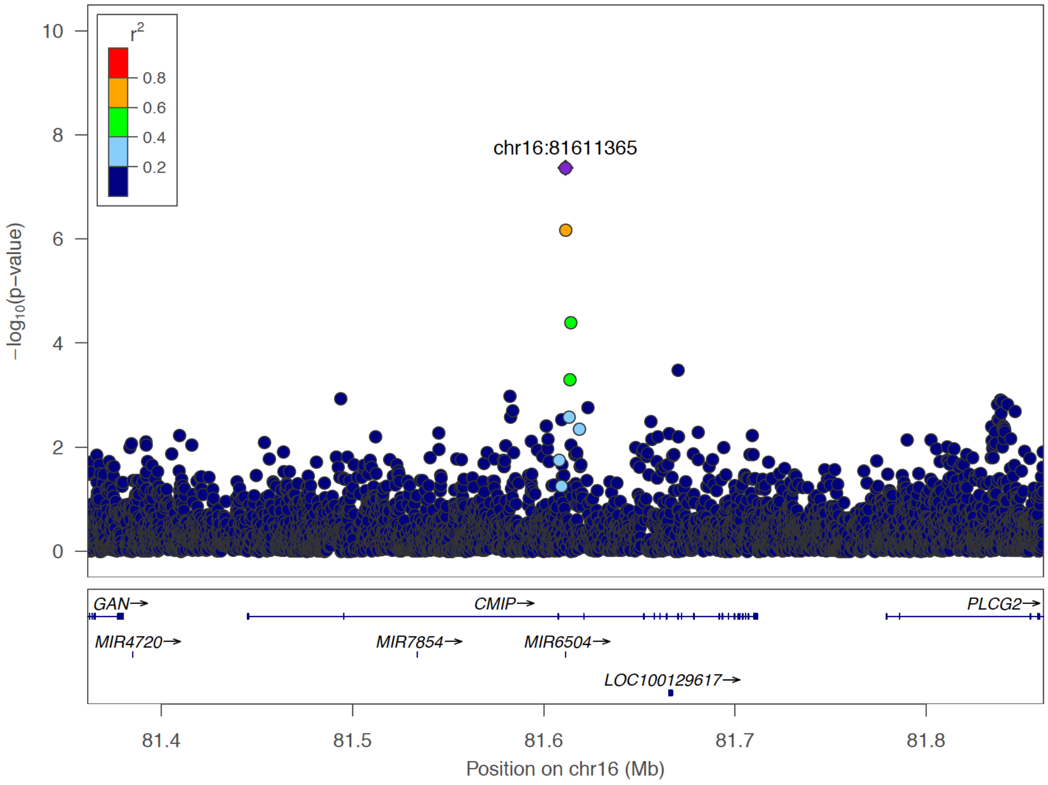

i) **FVC:** COPD-enriched, White; rs371740347 (chr1:196,989,333)

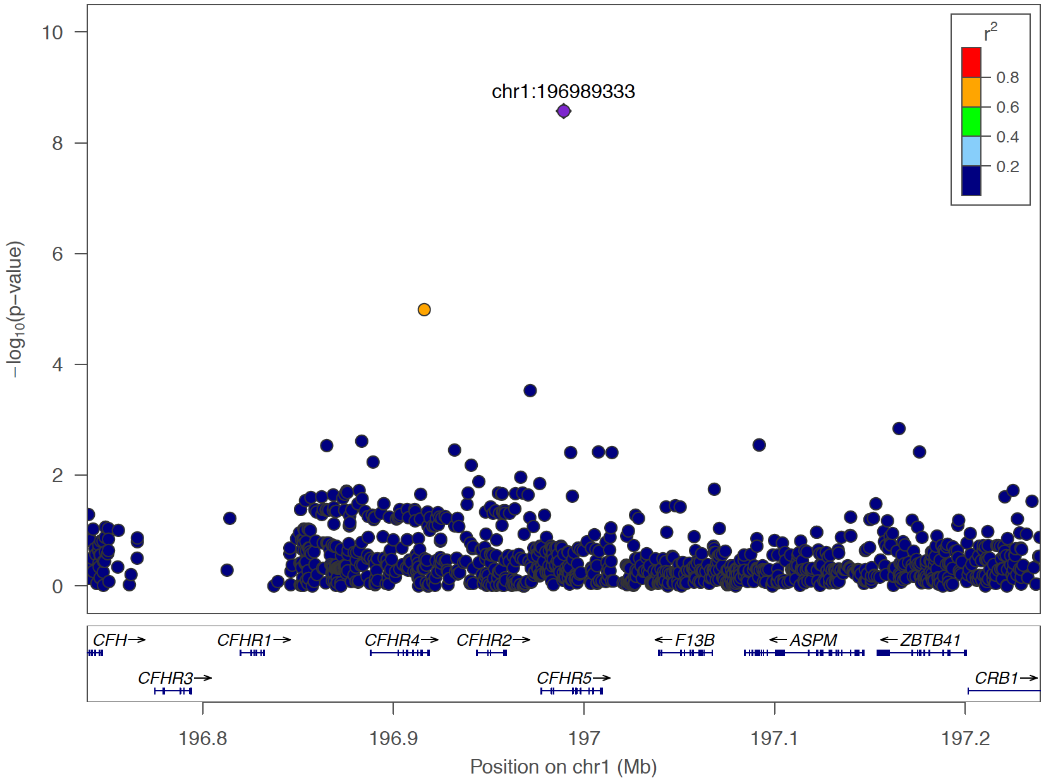

j) **FVC:** COPD-enriched, All; rs371740347 (chr1:196,989,333)

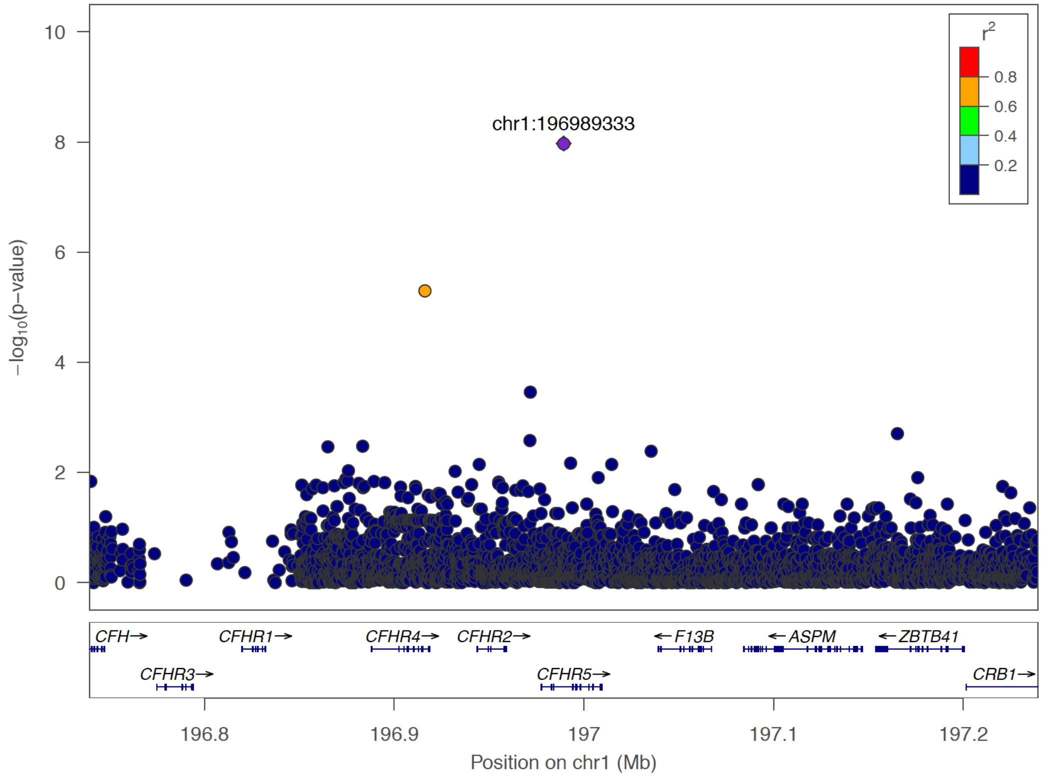

k) **FVC:** COPD-enriched, All; rs7046490 (chr9:673,533)

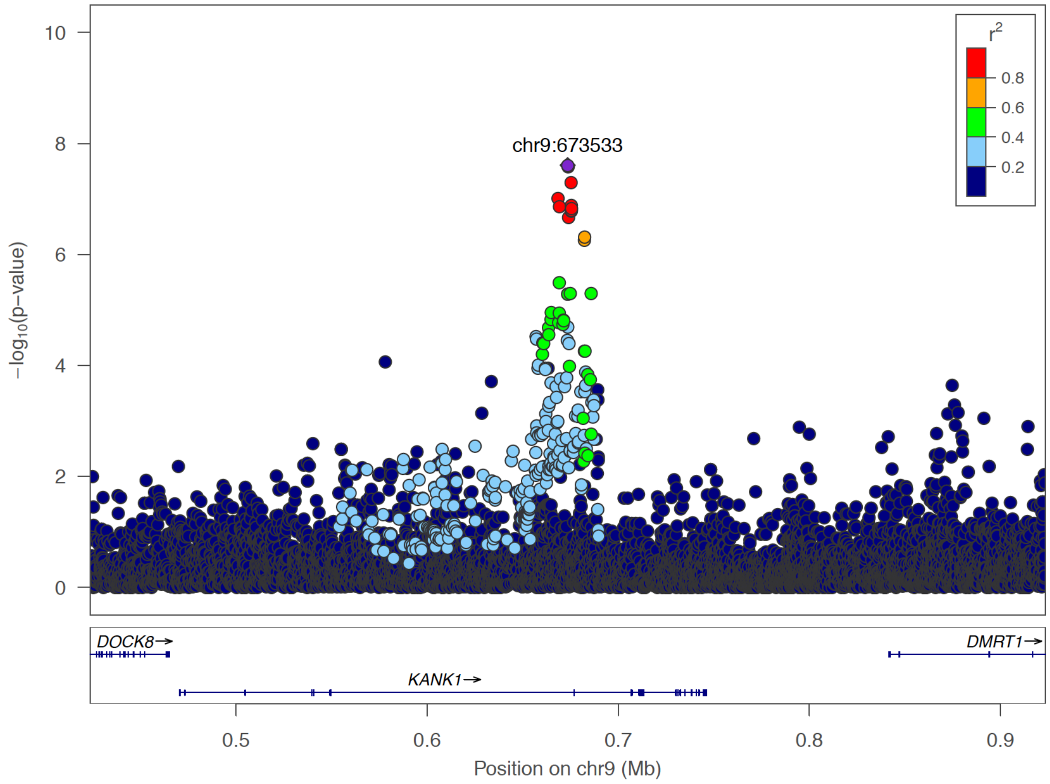

l) **FVC:** COPD-enriched, All; rs12556310 (chrX:47,087,005)

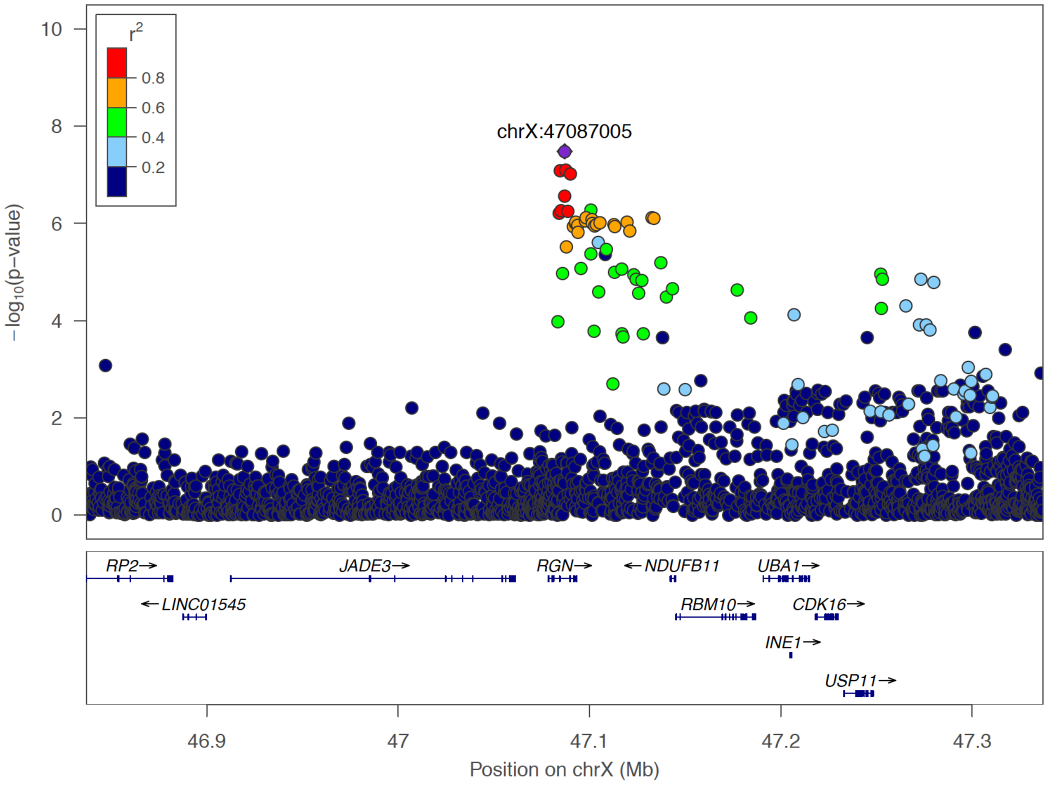

m) **FVC:** Combined, African American; rs56154976 (chr2:120,800,568)

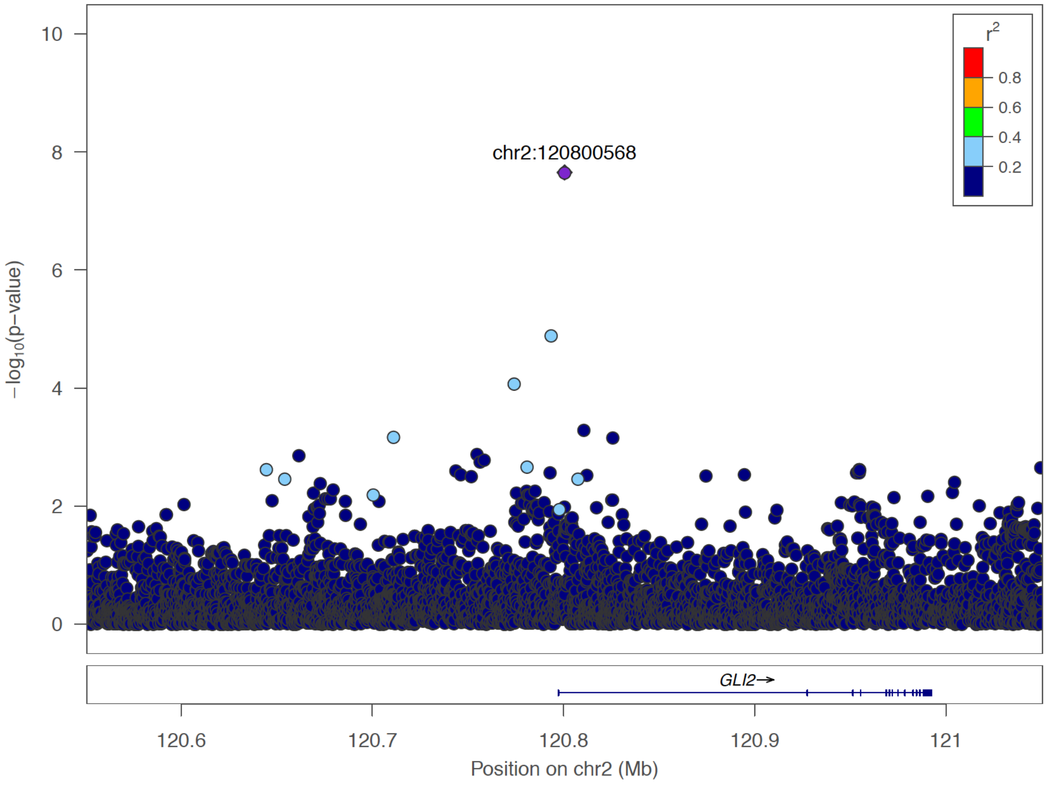

n) **FVC:** Combined, White; rs5953026 (chrX:47,317,317)

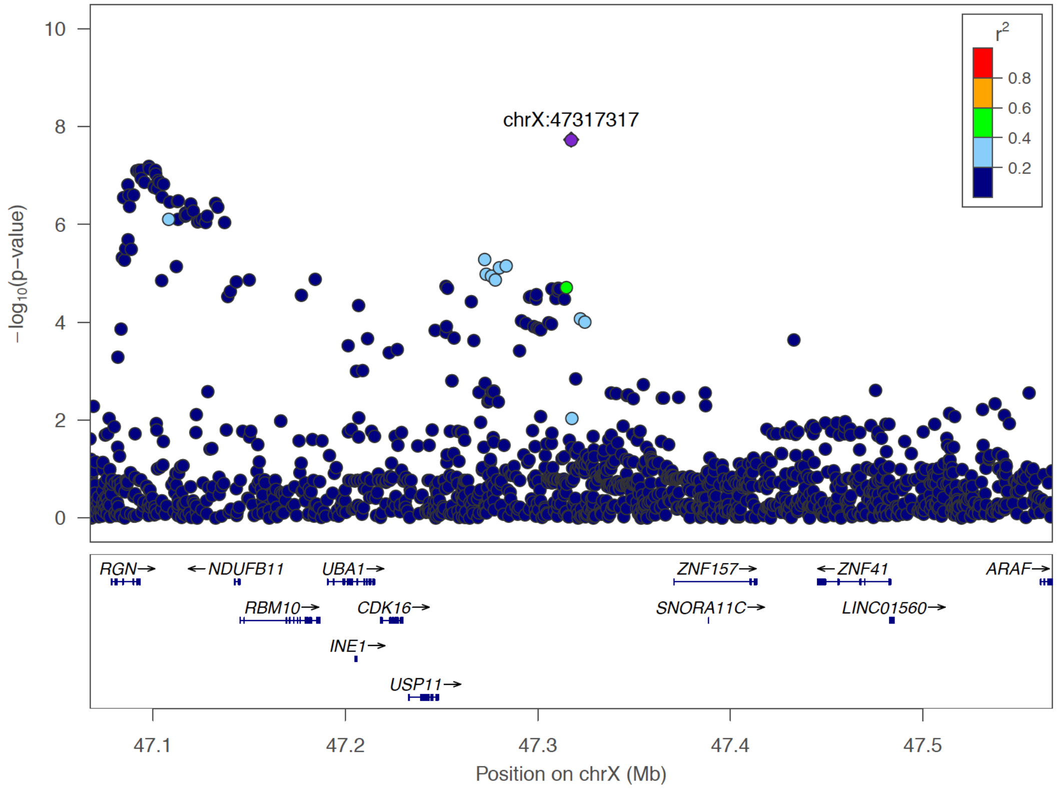

o) **FVC:** Combined, All; rs17308514 (chr15:68,020,833)

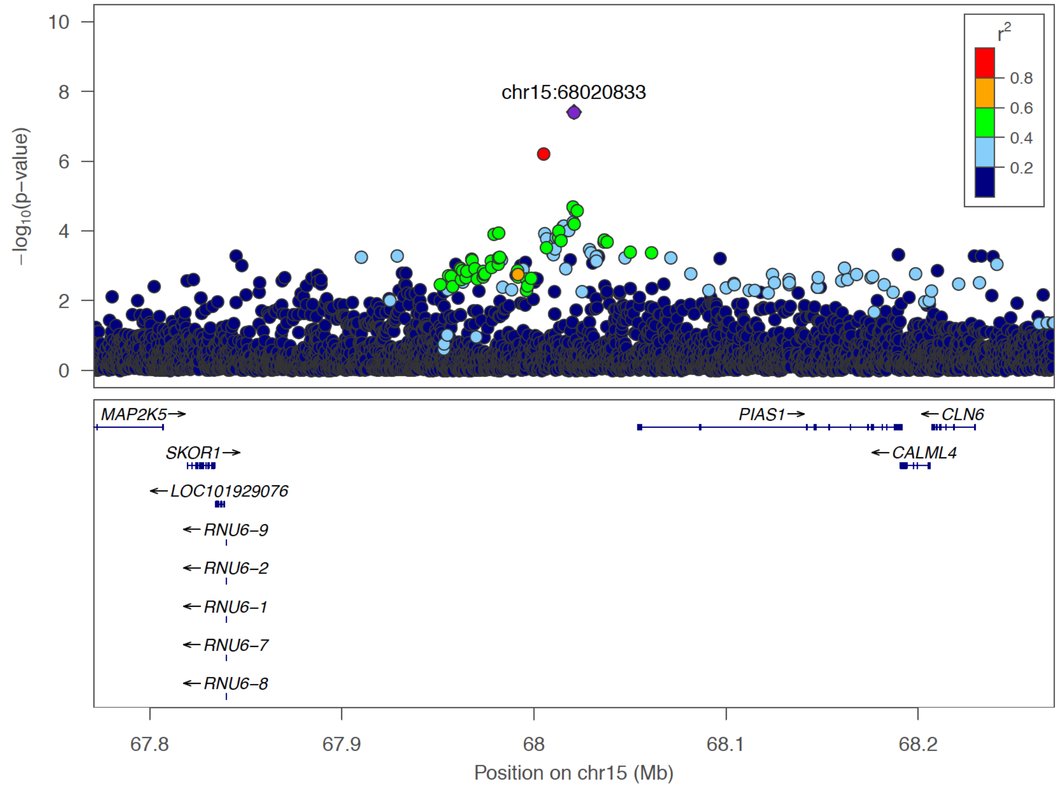

p) **FVC:** Combined, All; rs35917906 (chrX:47,100,766)

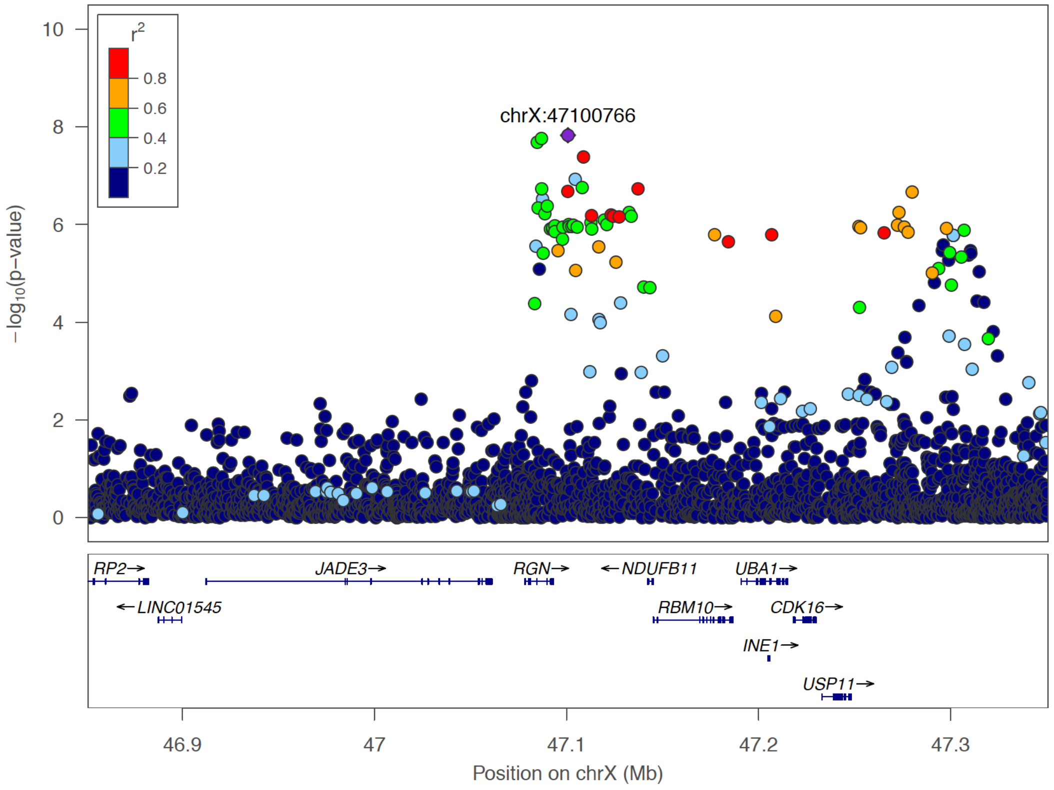

q) **FEV$_1$/FVC:** Population- and family-based, African American; rs145829100 (chr13:84795360)

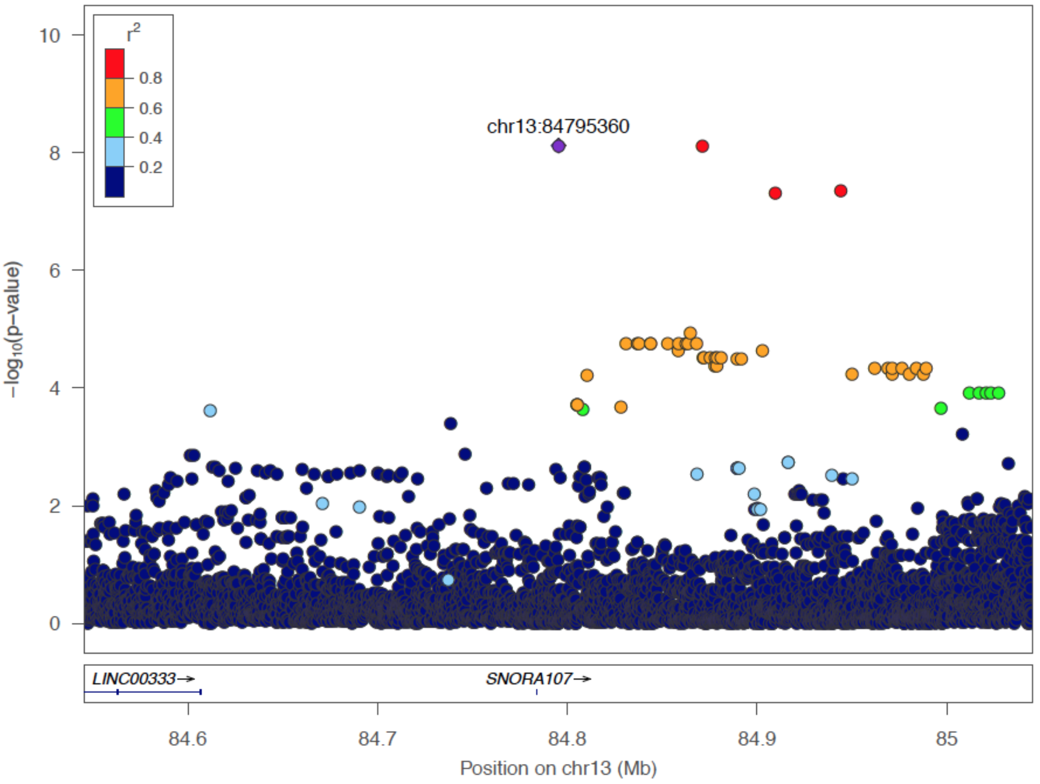

r) **FEV_1/FVC:** Population- and family-based, White; rs544345041 (chr7:30,969,275)

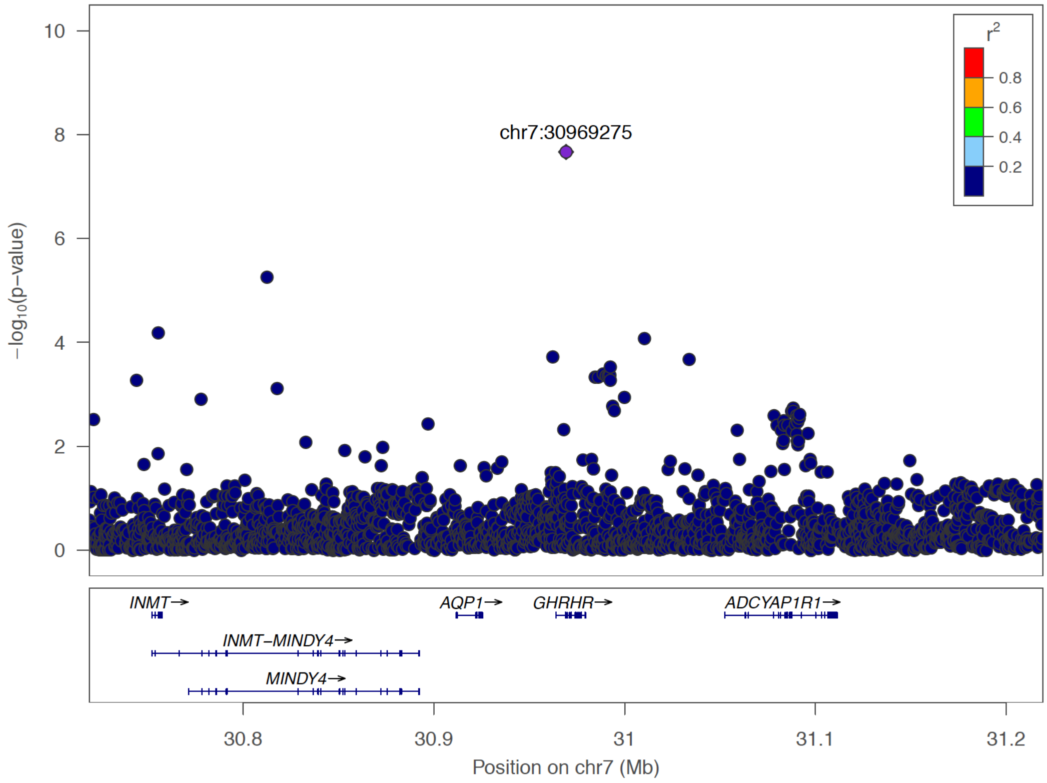

s) **FEV_1/FVC:** COPD-enriched, African American; rs144870669 (chr8:133,793,876)

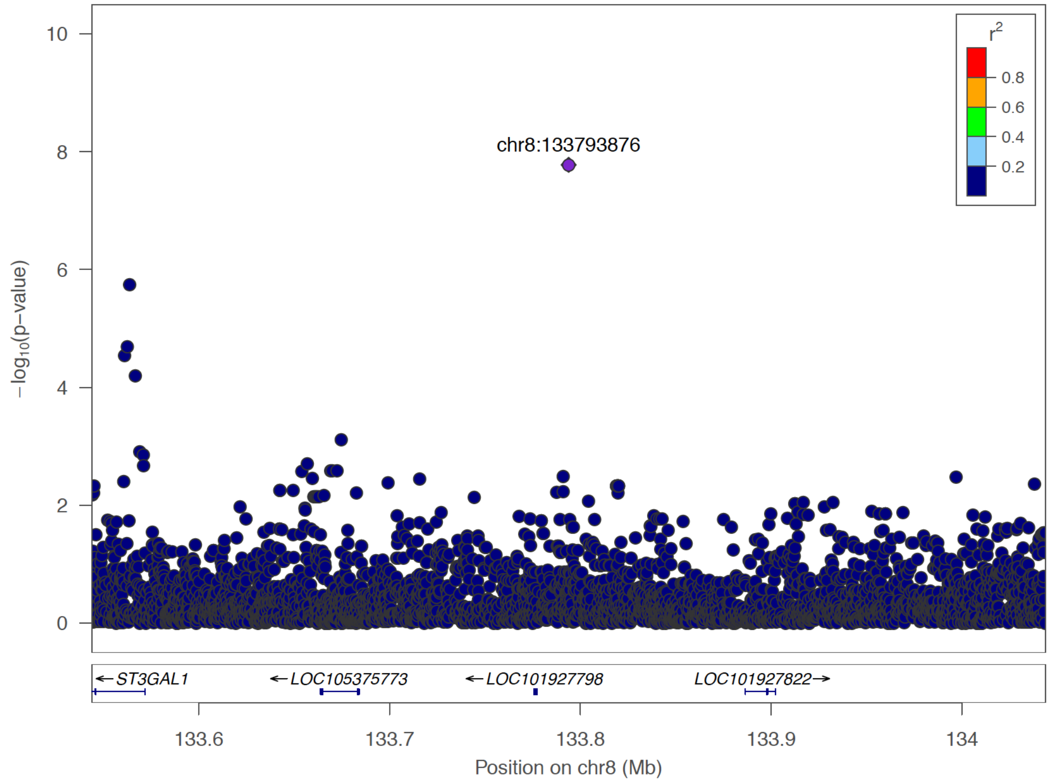

t) **FEV_1/FVC:** Combined, African American; rs1032155362 (chr6:107,949,366)

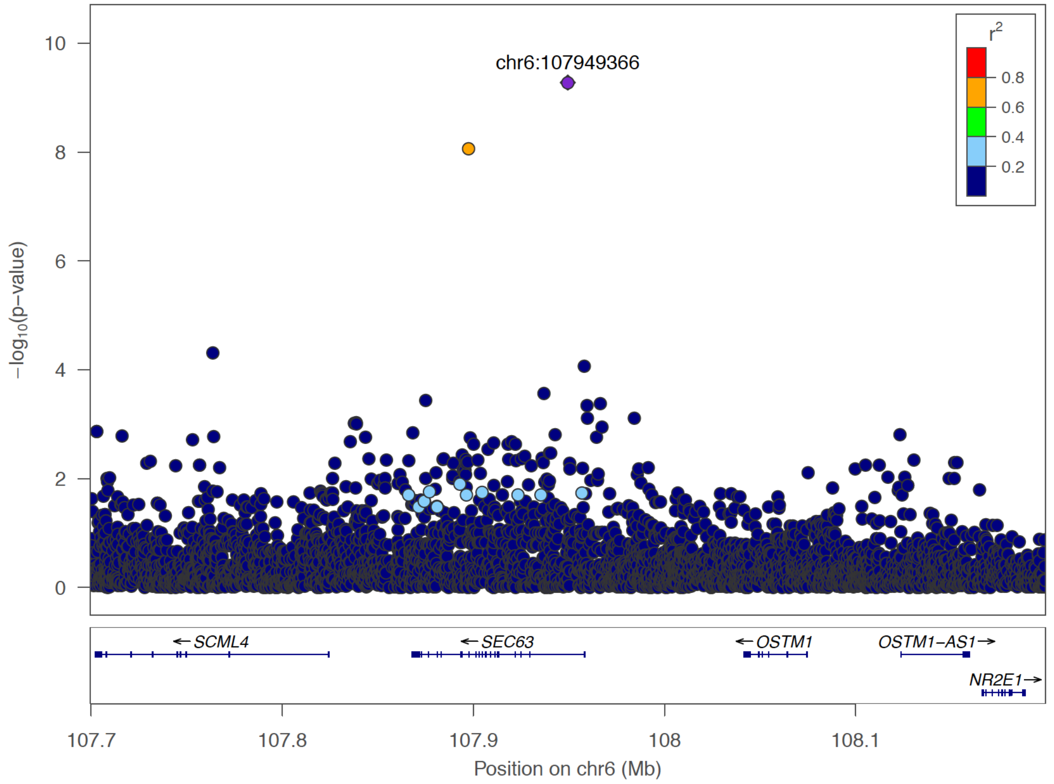

u) **FEV_1/FVC:** Combined, White; rs572153283 (chr6:102,490,266)

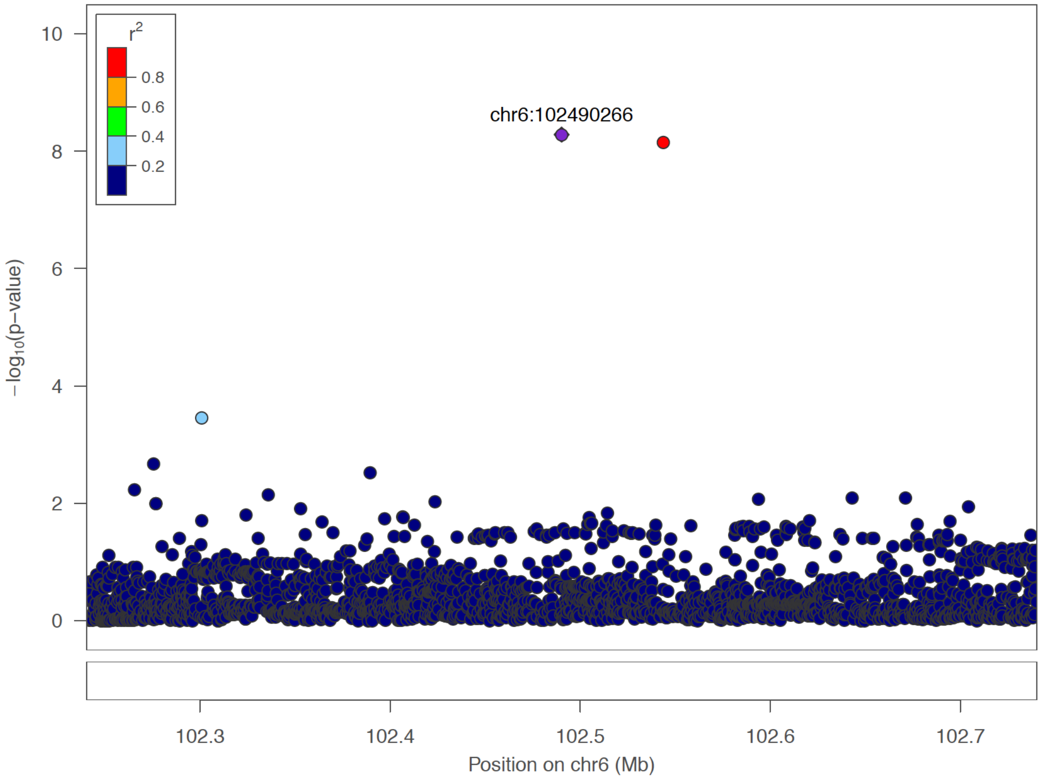

v) **FEV$_1$/FVC:** Combined, White; rs7188378 (chr16:53,872,940)

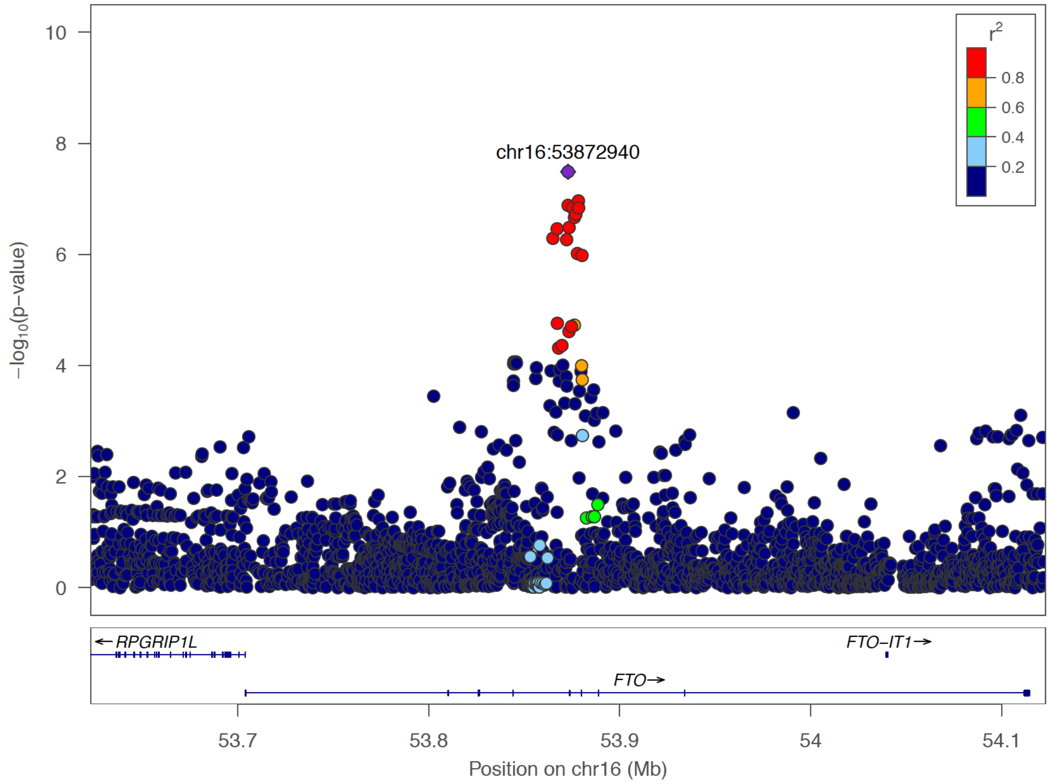

w) **FEV$_1$/FVC:** Combined, All; rs137938609 (chr6:102,543,833)

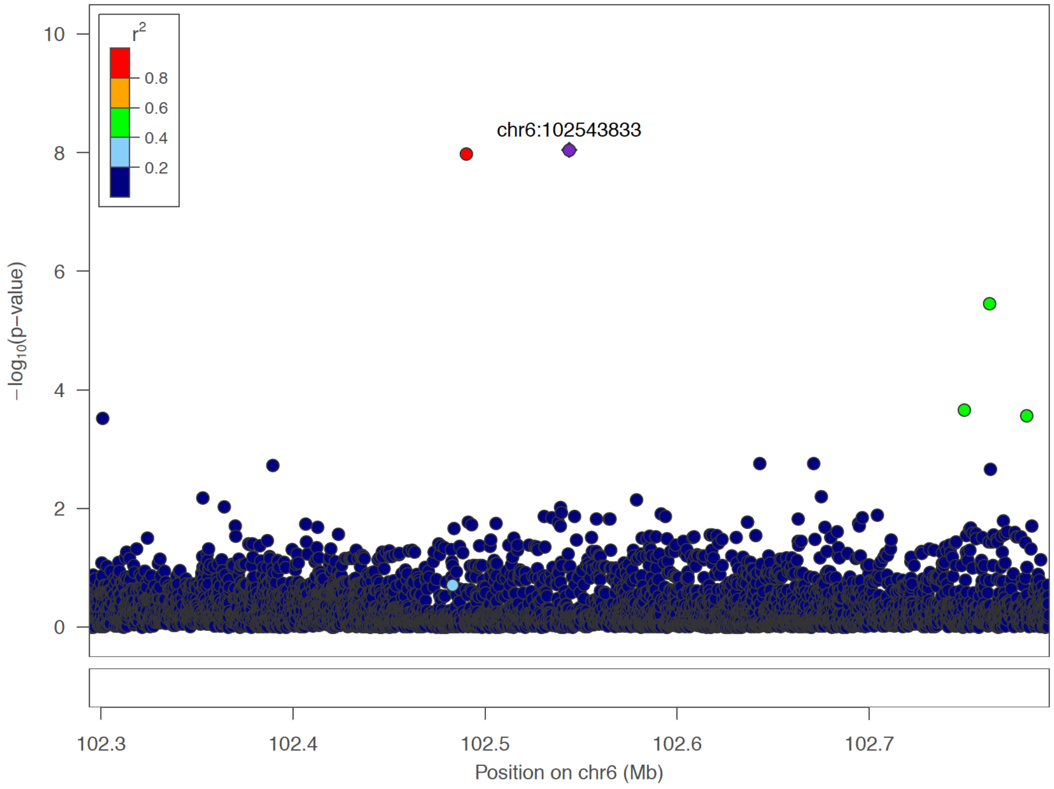

x) **FEV$_1$/FVC:** Combined, All; rs184101688 (chr7:7,140,556)

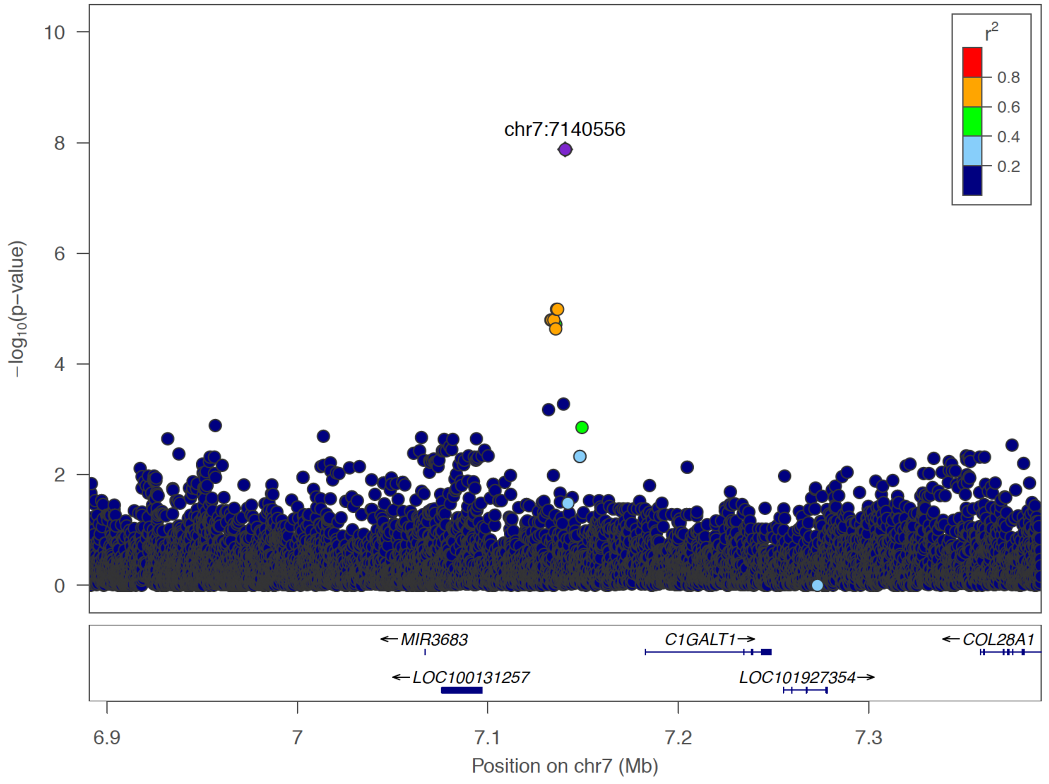

y) **FEV$_1$/FVC:** Combined, All; rs7188378 (chr16:53,872,940)

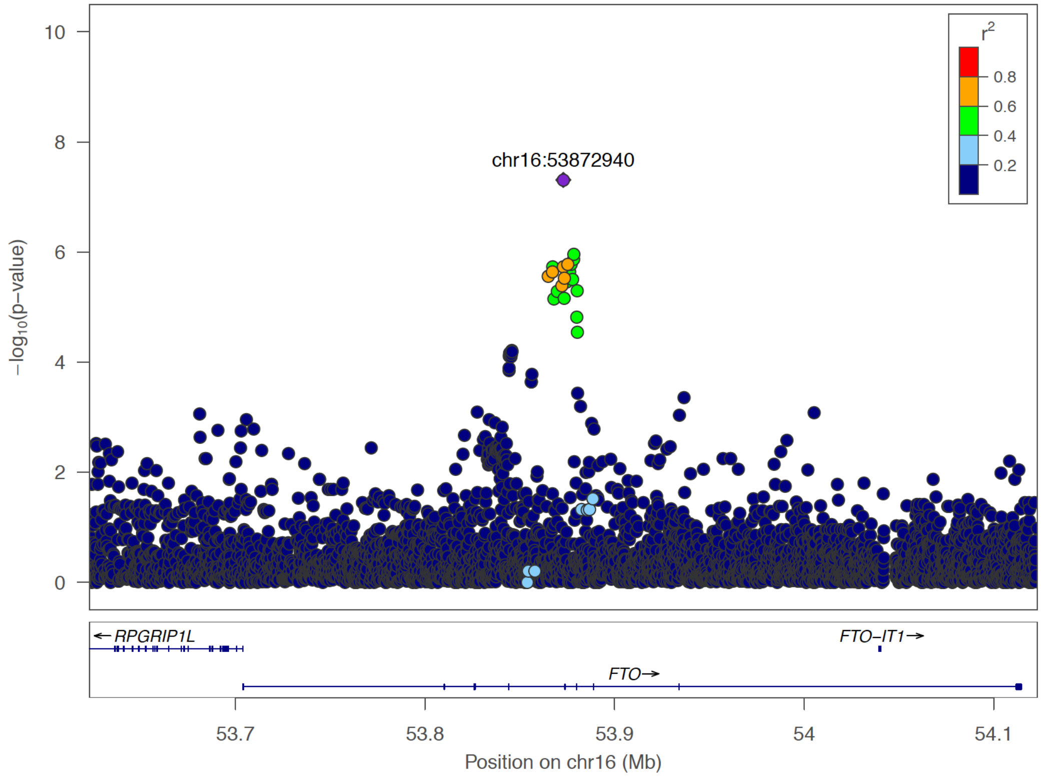

z) **Moderate-to-Severe COPD:** COPD-enriched, All; rs72740913 (chr15:53,390,153)

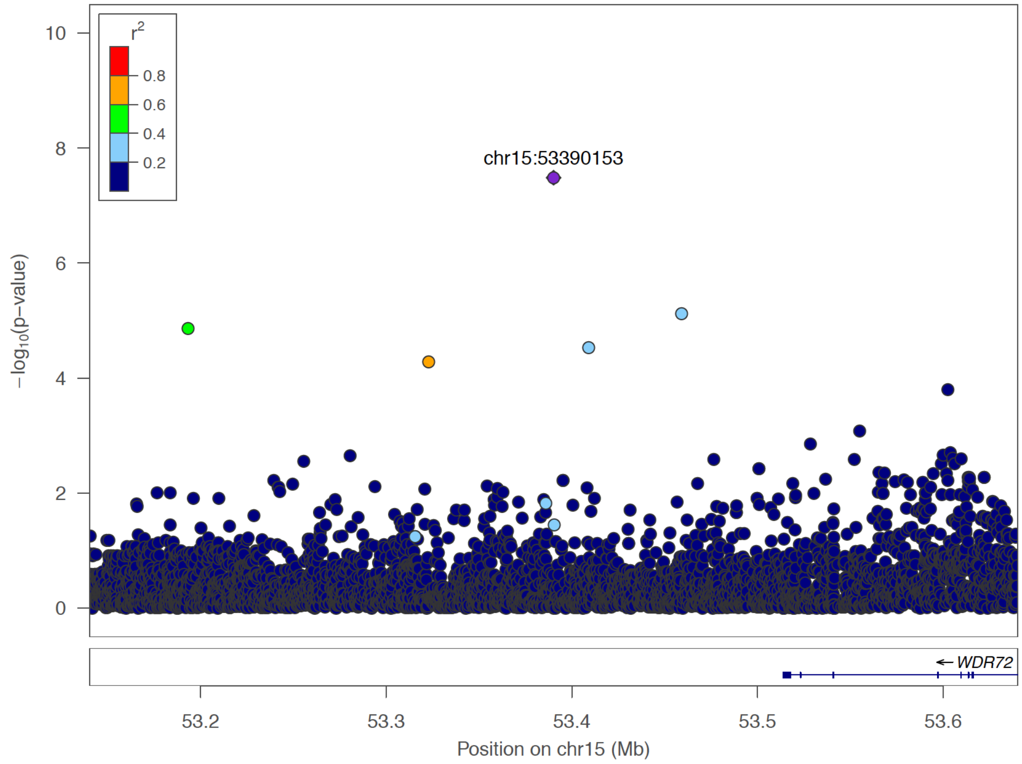

aa) **Severe COPD:** Combined, White; rs11687134 (chr2:141,400,617)

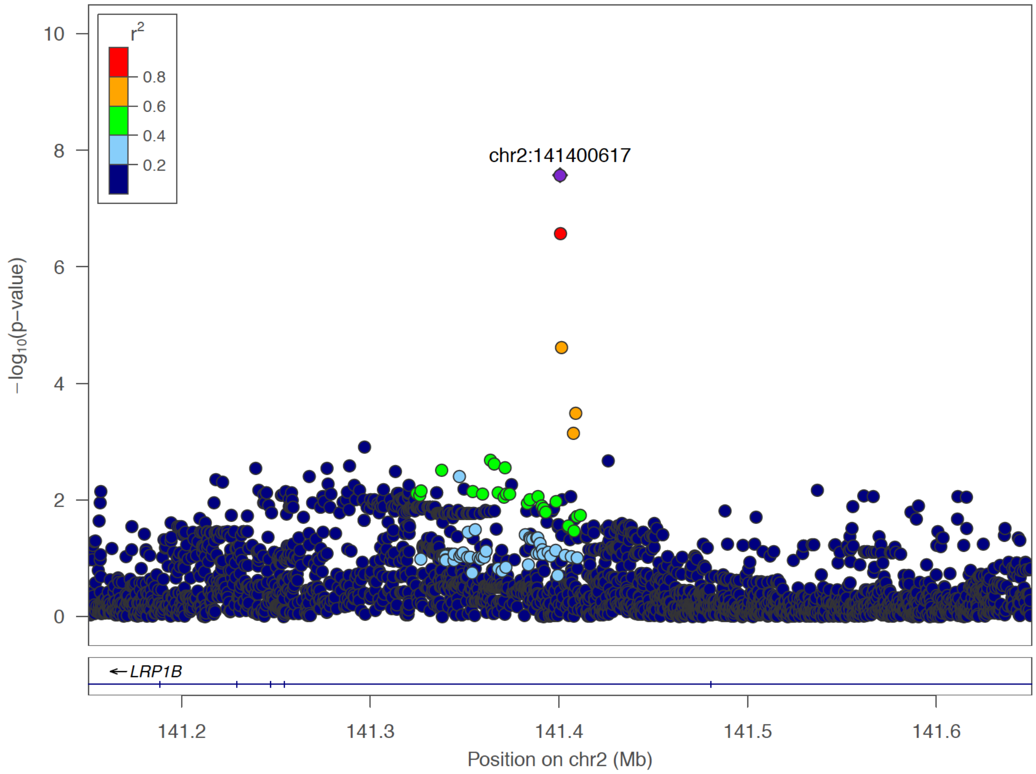

Supplementary Figure 3: Forest plots demonstrating cohort-specific and race/ethnic-specific effects for novel variants displayed in Supplementary Data 2. Supplementary Figures 3a-aa present the forest plots corresponding to each of the 27 genome-wide significant results at novel loci, in the same order as presented in Supplementary Data 2. Quantities displayed for each cohort or subgroup include the genetic additive effect (beta estimate from SAIGE score test[31]) +/- 95% confidence limits, derived as beta +/- 1.96 x standard error (of beta). The summary value is the effect estimate for the group used in the overall WGS discovery corresponding to the result in Supplementary Table 4. Studies with "*" are subgroups that contribute to the genome-wide significant summary results. Other cohort-specific results are shown for the purpose of comparison. Results are not shown in the plots when a subgroup does not contain the variant or the result does not converge.

a) **FEV_1:** Population- and family-based, African American; rs75195732 (chr17:69,937,351)

Study	n	Effect Size
ARIC_all	3256	0.876
ARIC_white	3075	
ARIC_black*	181	1.065
CFS_all	719	0.578
CFS_white	373	
CFS_black*	346	0.56
CHS_all	47	
CHS_white	39	
FHS_all	1835	
MESA_all	3252	0.294
MESA_white	1224	
MESA_black*	804	0.364
JHS_all*	2388	0.318
COPDGene_all	8444	-0.113
COPDGene_white	5713	
COPDGene_black	2731	-0.148
EOCOPD_all	55	
**Summary**	**19996**	**0.41**

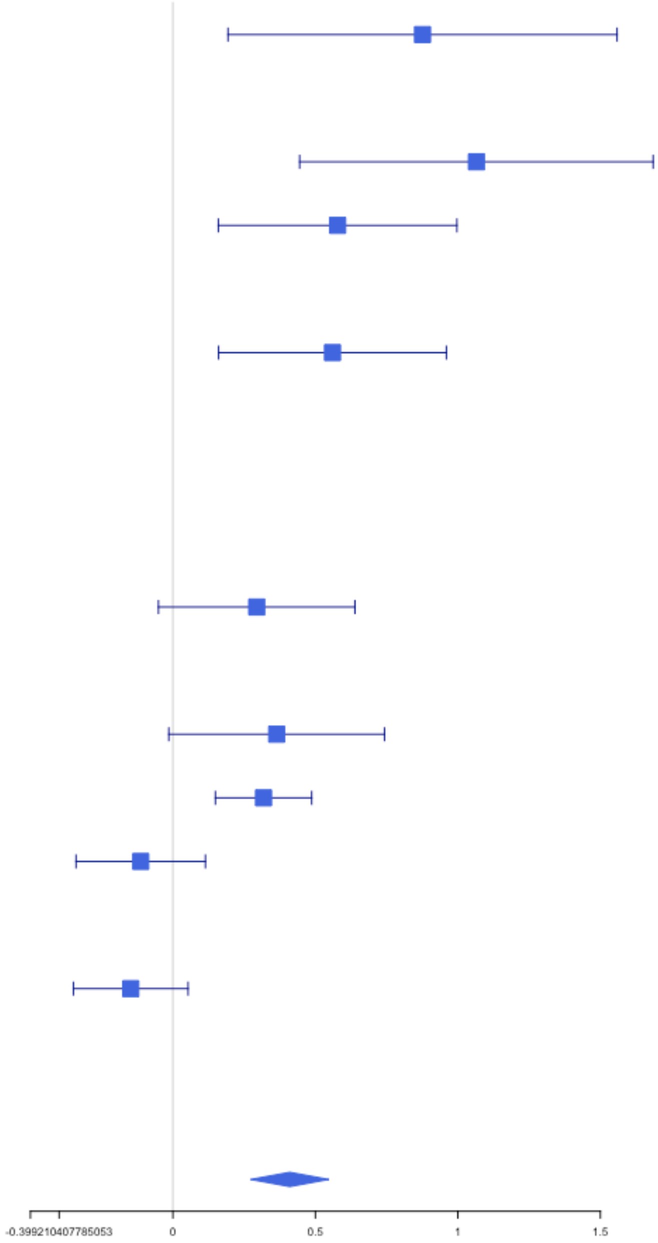

b) **FEV$_1$:** Population- and family-based, All; rs10984916 (chr9:120,443,427)

Study	n	Effect Size
ARIC_all*	3256	-0.107
ARIC_white	3075	-0.11
ARIC_black	181	0.198
CFS_all*	719	-0.083
CFS_white	373	-0.076
CFS_black	346	0.308
CHS_all*	47	0.063
CHS_white	39	0.263
FHS_all*	1835	-0.207
MESA_all*	3252	-0.161
MESA_white	1224	-0.189
MESA_black	804	-0.139
JHS_all*	2388	-0.142
COPDGene_all	8444	0.03
COPDGene_white	5713	0.032
COPDGene_black	2731	-0.032
EOCOPD_all	55	-0.188
**Summary**	**19996**	**-0.15**

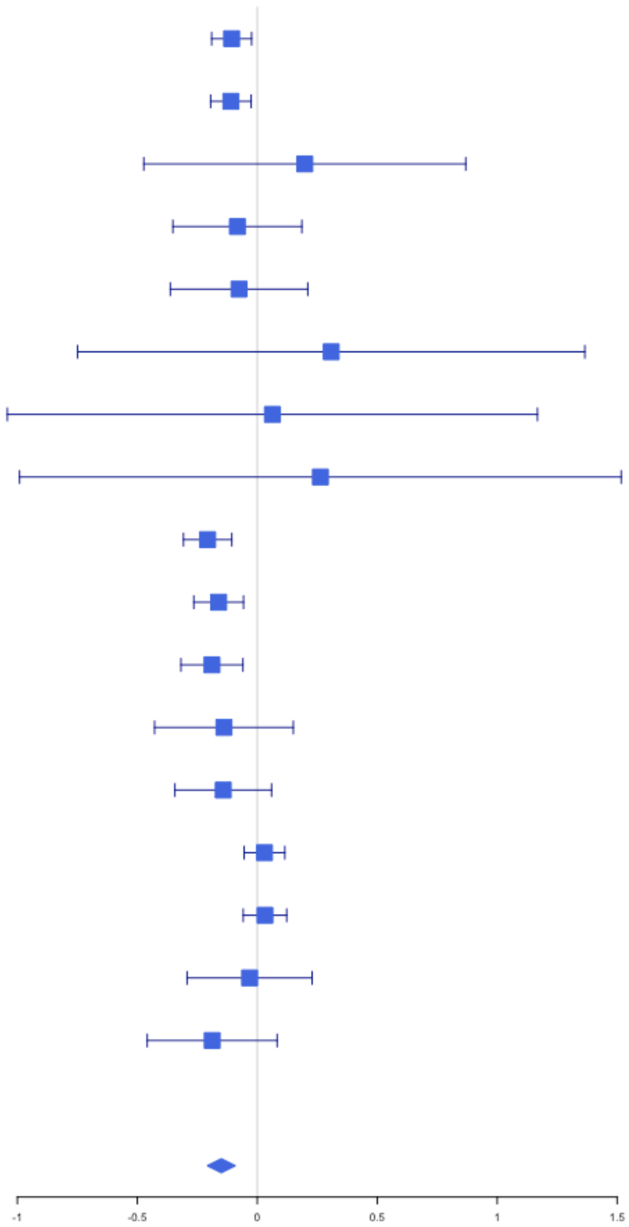

c) **FEV_1:** COPD-enriched, African American; rs4076943 (chr11:11,239,853)

Study	n	Effect Size
ARIC_all	3256	-0.016
ARIC_white	3075	-0.014
ARIC_black	181	-0.071
CFS_all	719	0.047
CFS_white	373	0.058
CFS_black	346	0.025
CHS_all	47	-0.045
CHS_white	39	0.059
FHS_all	1835	0.011
MESA_all	3252	0.003
MESA_white	1224	-0.001
MESA_black	804	0.011
JHS_all	2388	0.003
COPDGene_all	8444	0.03
COPDGene_white	5713	-0.004
COPDGene_black*	2731	0.124
EOCOPD_all	55	0.026
**Summary**	**19996**	**0.12**

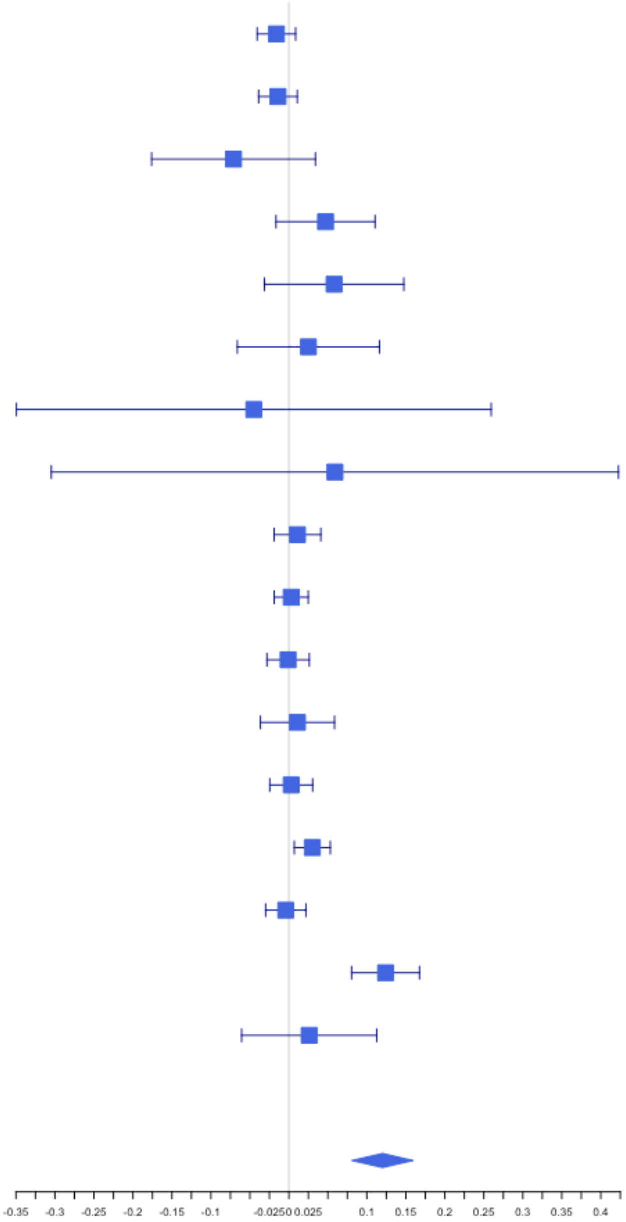

d) **FEV_1:** COPD-enriched, White; rs142755000 (chrX: 80,958,253)

Study	n	Effect Size
ARIC_all	3256	0.016
ARIC_white	3075	0.019
ARIC_black	181	-0.033
CFS_all	719	-0.147
CFS_white	373	-0.064
CFS_black	346	
CHS_all	47	-0.538
CHS_white	39	-0.503
FHS_all	1835	0.028
MESA_all	3252	-0.059
MESA_white	1224	0.003
MESA_black	804	-0.169
JHS_all	2388	0.087
COPDGene_all	8444	-0.161
COPDGene_white*	5713	-0.183
COPDGene_black	2731	0.109
EOCOPD_all*	55	-0.187
**Summary**	**19996**	**-0.18**

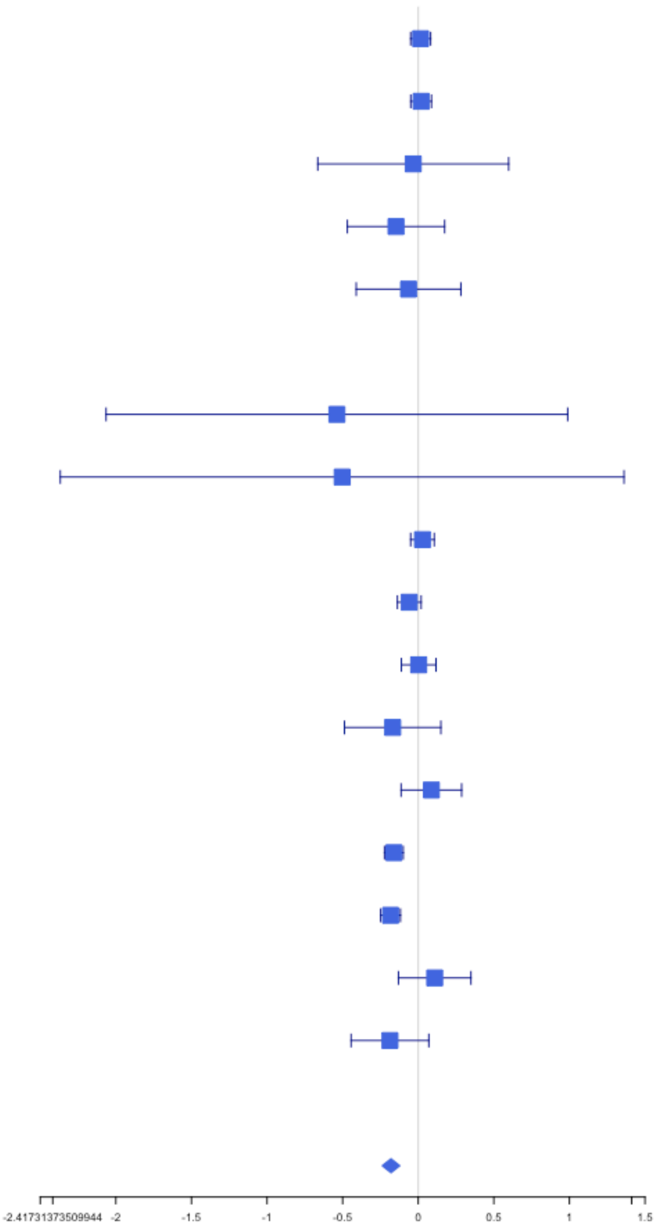

e) **FEV$_1$:** Combined, White; rs9295345 (chr6:166,400,303)

Study	n	Effect Size
ARIC_all	3256	-0.041
ARIC_white*	3075	-0.043
ARIC_black	181	-0.006
CFS_all	719	0.001
CFS_white*	373	0
CFS_black	346	0.001
CHS_all	47	0.184
CHS_white*	39	0.352
FHS_all*	1835	-0.03
MESA_all	3252	-0.018
MESA_white*	1224	-0.037
MESA_black	804	-0.011
JHS_all	2388	-0.014
COPDGene_all	8444	-0.035
COPDGene_white*	5713	-0.064
COPDGene_black	2731	0.015
EOCOPD_all*	55	0.07
**Summary**	**19996**	**-0.08**

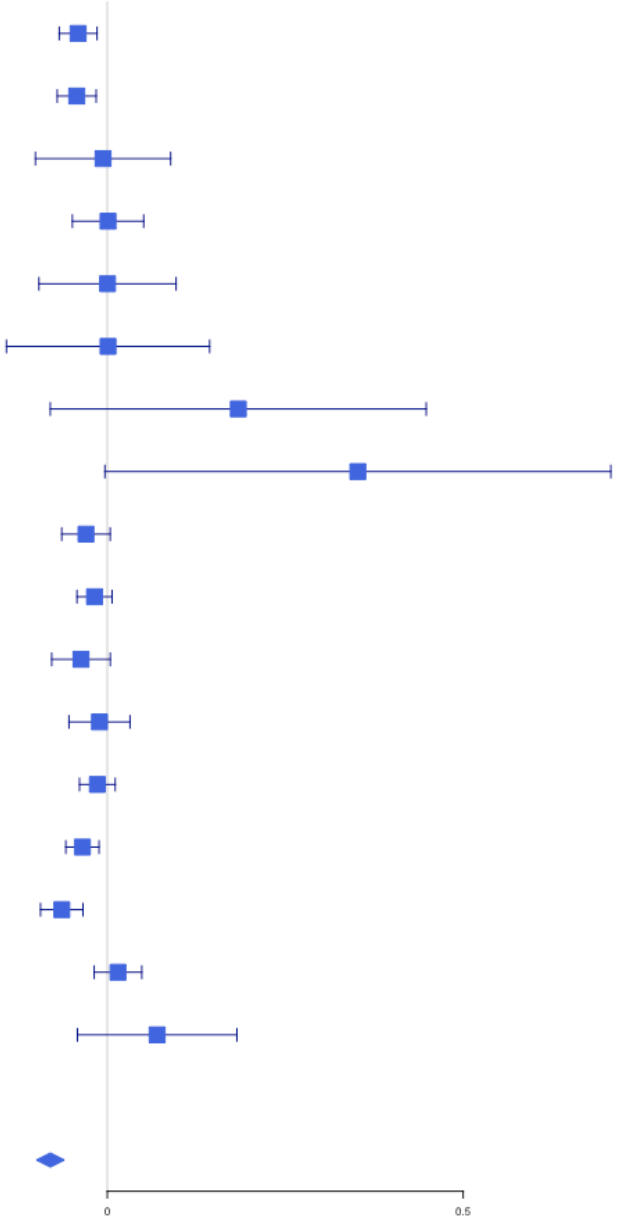

f) **FVC:** Population- and family-based, White; rs182915372 (chrX:46,687,945)

Study	n	Effect Size
ARIC_all	3256	-0.17
ARIC_white*	3075	-0.17
ARIC_black	181	
CFS_all	719	-0.129
CFS_white*	373	-0.203
CFS_black	346	-0.062
CHS_all	47	-1.419
CHS_white*	39	-1.415
FHS_all*	1835	-0.135
MESA_all	3252	-0.235
MESA_white*	1224	-0.274
MESA_black	804	-0.042
JHS_all	2388	-0.147
COPDGene_all	8444	-0.028
COPDGene_white	5713	-0.006
COPDGene_black	2731	-0.234
EOCOPD_all	55	-0.94
**Summary**	**19996**	**-0.19**

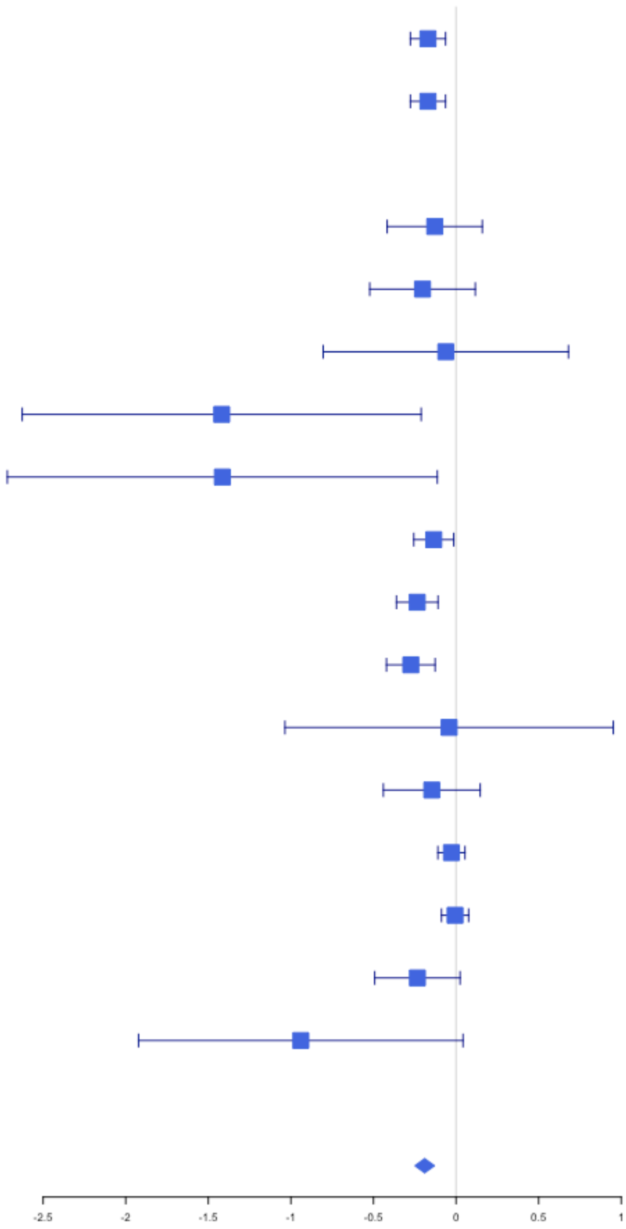

g) **FVC:** Population- and family-based, All; rs182915372 (chrX:46,687,945)

Study	n	Effect Size
ARIC_all*	3256	-0.17
ARIC_white	3075	-0.17
ARIC_black	181	
CFS_all*	719	-0.129
CFS_white	373	-0.203
CFS_black	346	-0.062
CHS_all*	47	-1.419
CHS_white	39	-1.415
FHS_all*	1835	-0.135
MESA_all*	3252	-0.235
MESA_white	1224	-0.274
MESA_black	804	-0.042
JHS_all*	2388	-0.147
COPDGene_all	8444	-0.028
COPDGene_white	5713	-0.006
COPDGene_black	2731	-0.234
EOCOPD_all	55	-0.94
**Summary**	**19996**	**-0.18**

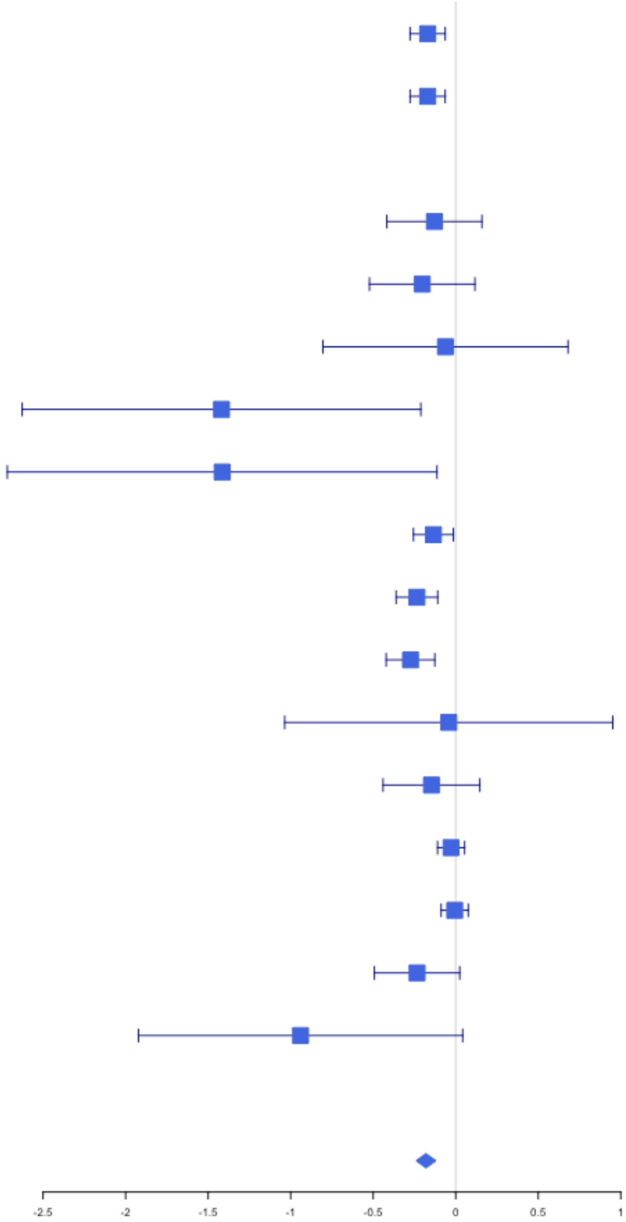

h) **FVC:** COPD-enriched, African American; rs74469188 (chr16:81,611,365)

Study	n	Effect Size
ARIC_all	3256	-0.016
ARIC_white	3075	-0.02
ARIC_black	181	0.123
CFS_all	719	-0.052
CFS_white	373	-0.07
CFS_black	346	-0.046
CHS_all	47	-0.452
CHS_white	39	-0.581
FHS_all	1835	-0.021
MESA_all	3252	-0.006
MESA_white	1224	-0.025
MESA_black	804	0.044
JHS_all	2388	-0.001
COPDGene_all	8444	0.046
COPDGene_white	5713	-0.006
COPDGene_black*	2731	0.143
EOCOPD_all	55	-0.217
**Summary**	**19996**	**0.14**

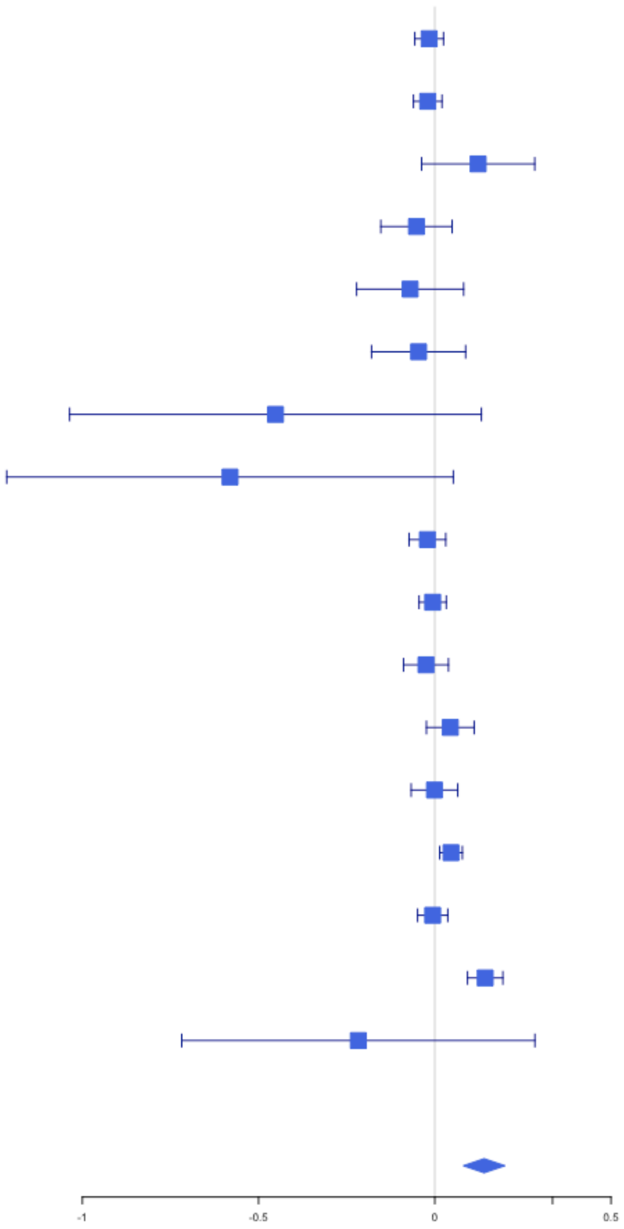

i) **FVC:** COPD-enriched, White; rs371740347 (chr1:196,989,333)

Study	n	Effect Size
ARIC_all	3256	0.037
ARIC_white	3075	0.033
ARIC_black	181	
CFS_all	719	0.292
CFS_white	373	0.225
CFS_black	346	0.336
CHS_all	47	
CHS_white	39	
FHS_all	1835	-0.012
MESA_all	3252	-0.071
MESA_white	1224	0.041
MESA_black	804	-0.634
JHS_all	2388	0.155
COPDGene_all	8444	0.388
COPDGene_white*	5713	0.433
COPDGene_black	2731	-0.054
EOCOPD_all*	55	0.065
**Summary**	**19996**	**0.43**

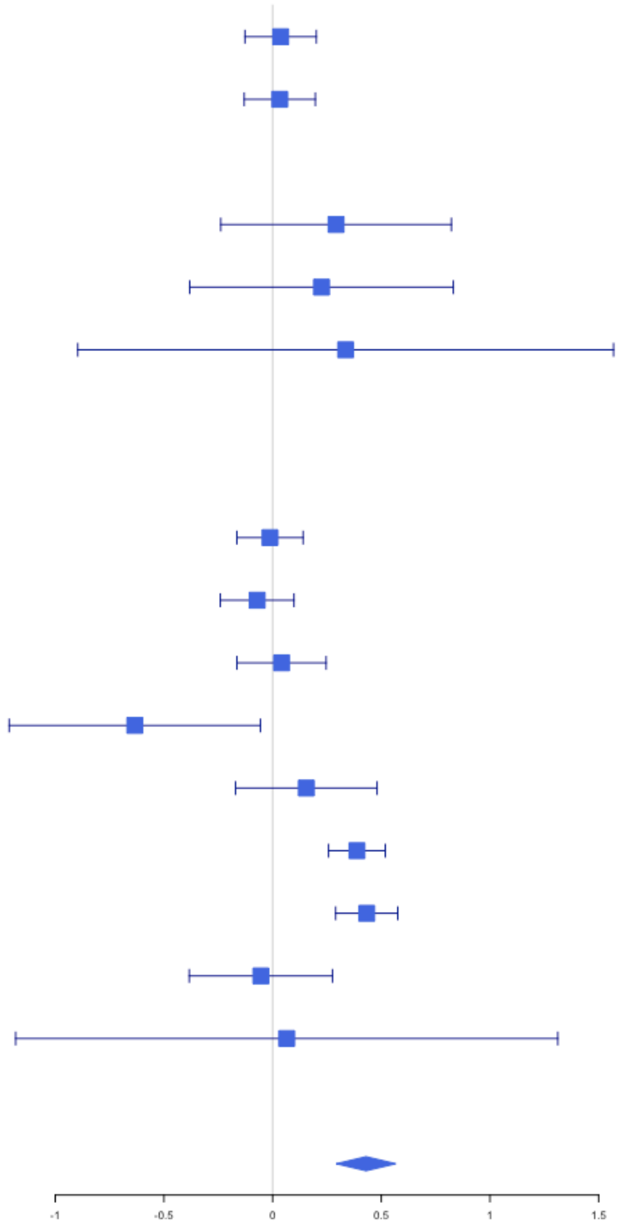

j) **FVC:** COPD-enriched, All; rs371740347 (chr1:196,989,333)

Study	n	Effect Size
ARIC_all	3256	0.037
ARIC_white	3075	0.033
ARIC_black	181	
CFS_all	719	0.292
CFS_white	373	0.225
CFS_black	346	0.336
CHS_all	47	
CHS_white	39	
FHS_all	1835	-0.012
MESA_all	3252	-0.071
MESA_white	1224	0.041
MESA_black	804	-0.634
JHS_all	2388	0.155
COPDGene_all*	8444	0.388
COPDGene_white	5713	0.433
COPDGene_black	2731	-0.054
EOCOPD_all*	55	0.065
**Summary**	**19996**	**0.38**

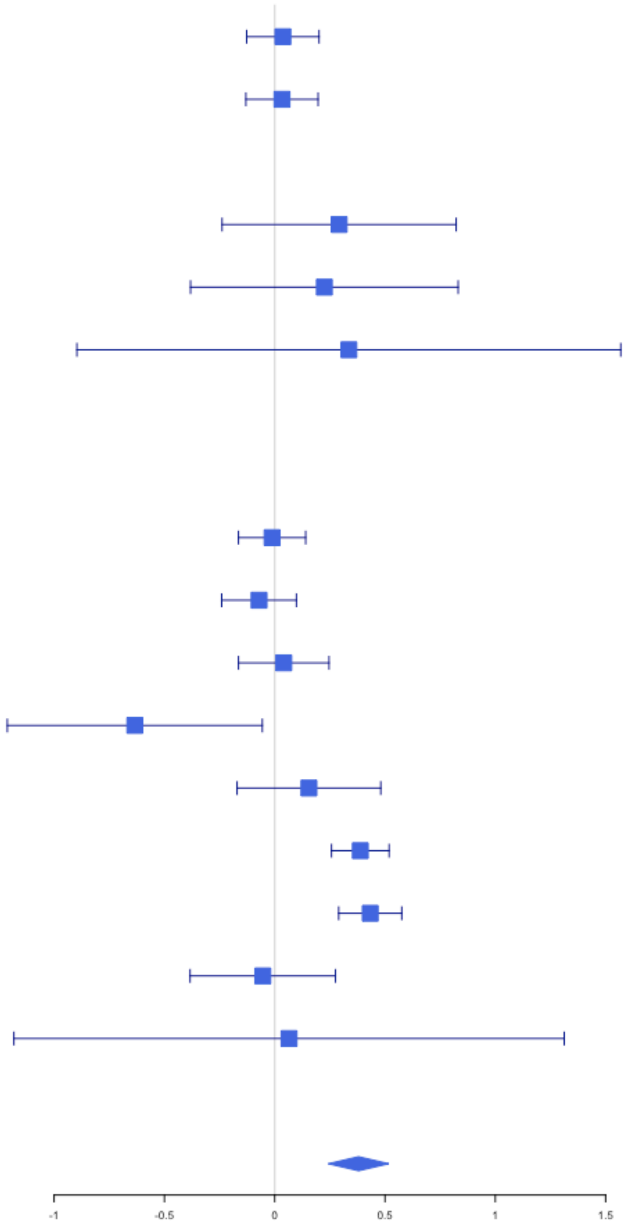

k) **FVC:** COPD-enriched, All; rs7046490 (chr9:673,533)

Study	n	Effect Size
ARIC_all	3256	0.027
ARIC_white	3075	0.025
ARIC_black	181	0.07
CFS_all	719	-0.004
CFS_white	373	0.071
CFS_black	346	-0.045
CHS_all	47	0.153
CHS_white	39	0.181
FHS_all	1835	-0.005
MESA_all	3252	0
MESA_white	1224	-0.018
MESA_black	804	0.029
JHS_all	2388	0.003
COPDGene_all*	8444	0.063
COPDGene_white	5713	0.066
COPDGene_black	2731	0.047
EOCOPD_all*	55	0.035
**Summary**	**19996**	**0.06**

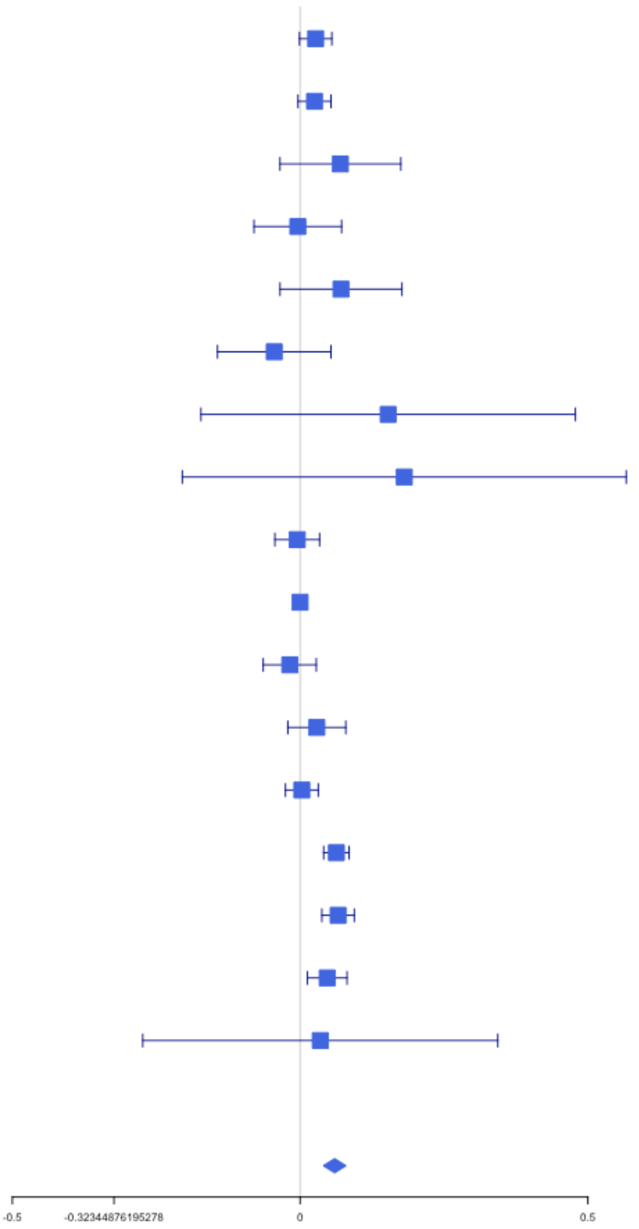

l) **FVC:** COPD-enriched, All; rs12556310 (chrX:47,087,005)

Study	n	Effect Size
ARIC_all	3256	-0.019
ARIC_white	3075	-0.019
ARIC_black	181	0.011
CFS_all	719	-0.018
CFS_white	373	-0.038
CFS_black	346	0.005
CHS_all	47	-0.152
CHS_white	39	-0.196
FHS_all	1835	0.003
MESA_all	3252	-0.018
MESA_white	1224	-0.025
MESA_black	804	-0.01
JHS_all	2388	0
COPDGene_all*	8444	0.05
COPDGene_white	5713	-0.056
COPDGene_black	2731	-0.03
EOCOPD_all*	55	0.075
**Summary**	**19996**	**0.05**

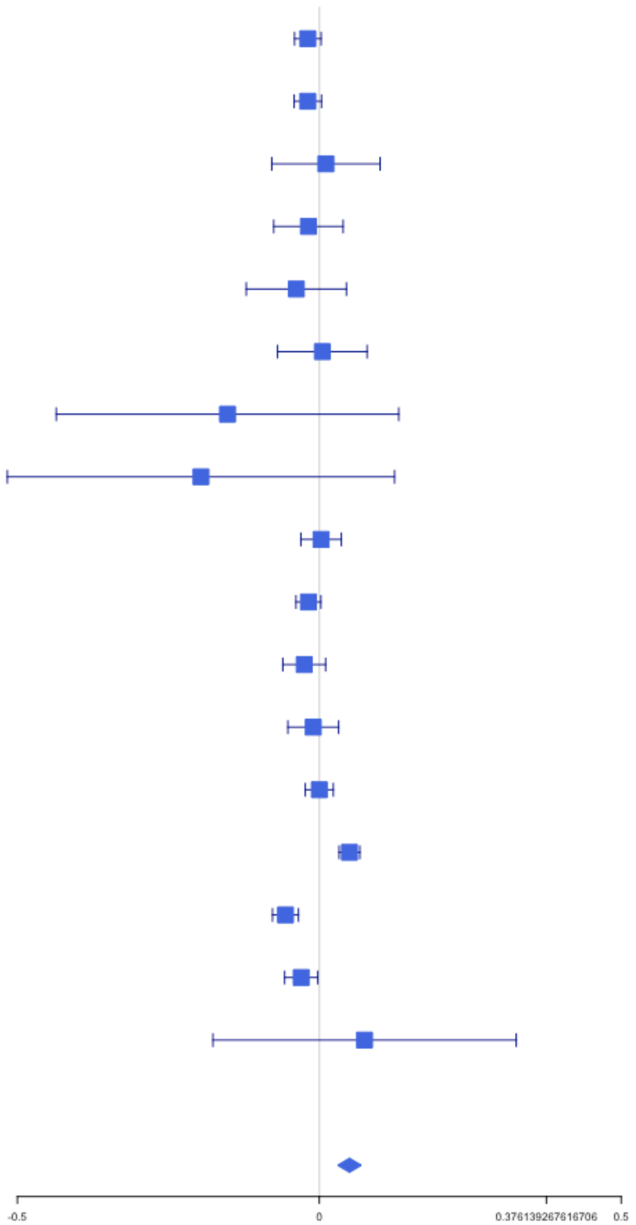

m) **FVC:** Combined, African American; rs56154976 (chr2:120,800,568)

Study	n	Effect Size
ARIC_all	3256	0.01
ARIC_white	3075	0.008
ARIC_black*	181	0.21
CFS_all	719	-0.032
CFS_white	373	-0.052
CFS_black*	346	-0.054
CHS_all	47	-0.046
CHS_white	39	-0.086
FHS_all	1835	-0.026
MESA_all	3252	0.014
MESA_white	1224	0.013
MESA_black*	804	0.134
JHS_all*	2388	0.126
COPDGene_all	8444	0.037
COPDGene_white	5713	0.001
COPDGene_black*	2731	0.2
EOCOPD_all	55	0.014
**Summary**	**19996**	**0.16**

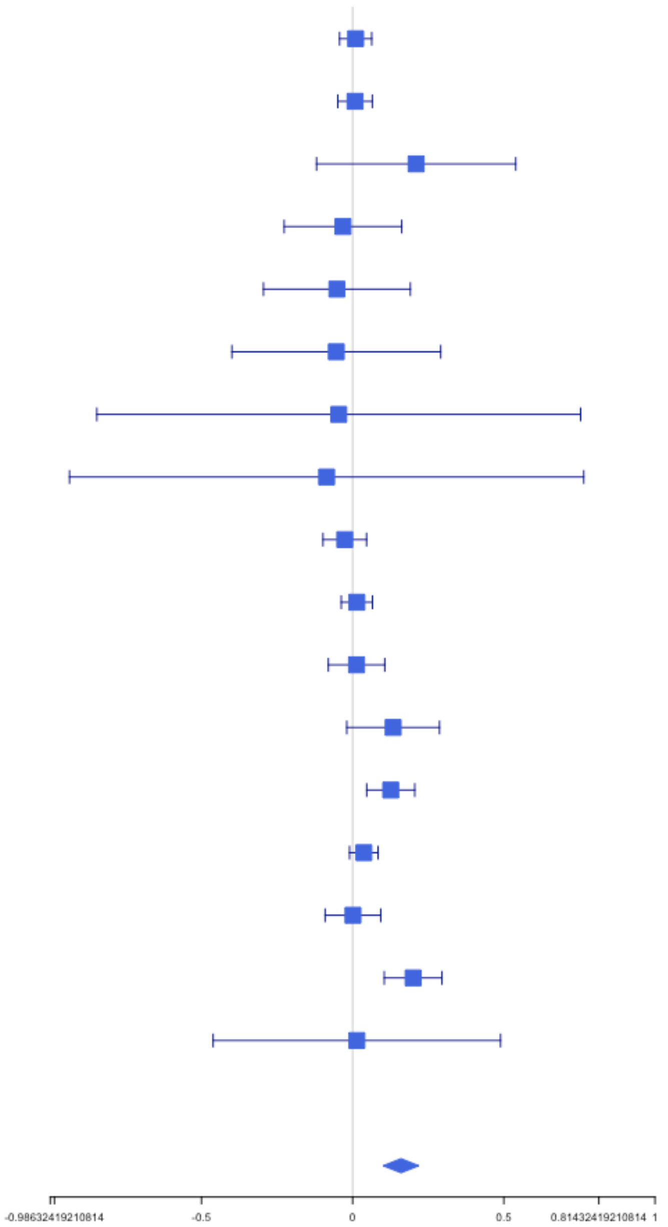

n) **FVC:** Combined, White; rs5953026 (chrX:47,317,317)

Study	n	Effect Size
ARIC_all	3256	0.027
ARIC_white*	3075	0.026
ARIC_black	181	-0.053
CFS_all	719	0.065
CFS_white*	373	0.094
CFS_black	346	-0.019
CHS_all	47	0.228
CHS_white*	39	0.214
FHS_all*	1835	0.005
MESA_all	3252	0.001
MESA_white*	1224	0.018
MESA_black	804	0.032
JHS_all	2388	-0.004
COPDGene_all	8444	0.032
COPDGene_white*	5713	0.053
COPDGene_black	2731	-0.018
EOCOPD_all*	55	0.082
**Summary**	**19996**	**0.04**

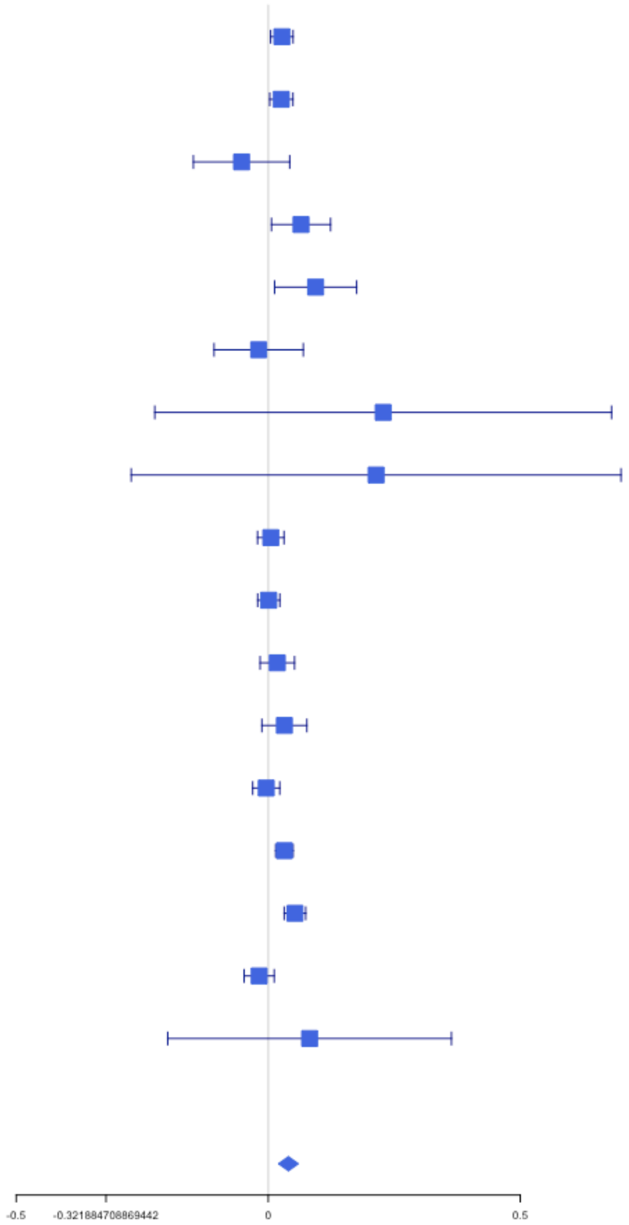

o) **FVC:** Combined, All; rs17308514 (chr15:68,020,833)

Study	n	Effect Size
ARIC_all*	3256	-0.033
ARIC_white	3075	-0.035
ARIC_black	181	0.112
CFS_all*	719	-0.035
CFS_white	373	-0.008
CFS_black	346	-0.072
CHS_all*	47	-0.034
CHS_white	39	0.012
FHS_all*	1835	-0.024
MESA_all*	3252	-0.017
MESA_white	1224	-0.025
MESA_black	804	-0.049
JHS_all*	2388	-0.029
COPDGene_all*	8444	-0.052
COPDGene_white	5713	-0.051
COPDGene_black	2731	-0.058
EOCOPD_all*	55	0.055
**Summary**	**19996**	**-0.04**

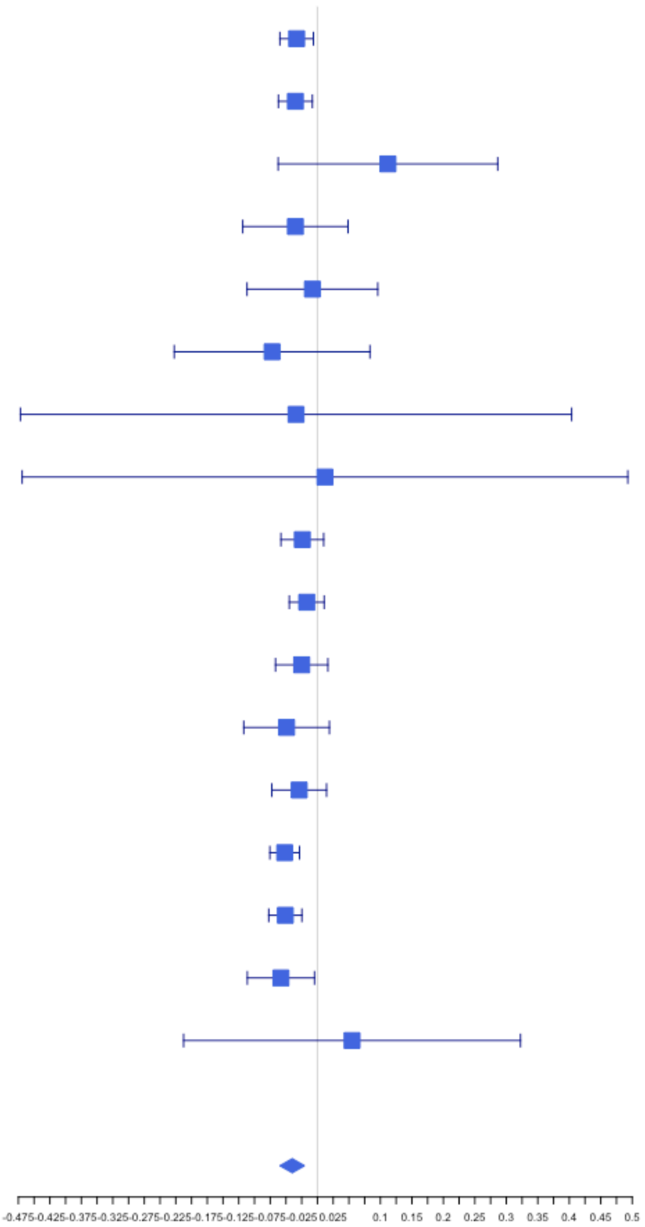

p) **FVC:** Combined, All; rs35917906 (chrX:47,100,766)

Study	n	Effect Size
ARIC_all*	3256	0.019
ARIC_white	3075	0.02
ARIC_black	181	0.052
CFS_all*	719	0.046
CFS_white	373	0.075
CFS_black	346	-0.01
CHS_all*	47	0.402
CHS_white	39	0.425
FHS_all*	1835	0.003
MESA_all*	3252	0.027
MESA_white	1224	0.031
MESA_black	804	0.03
JHS_all*	2388	-0.005
COPDGene_all*	8444	0.051
COPDGene_white	5713	0.051
COPDGene_black	2731	0.042
EOCOPD_all*	55	0.133
**Summary**	**19996**	**0.03**

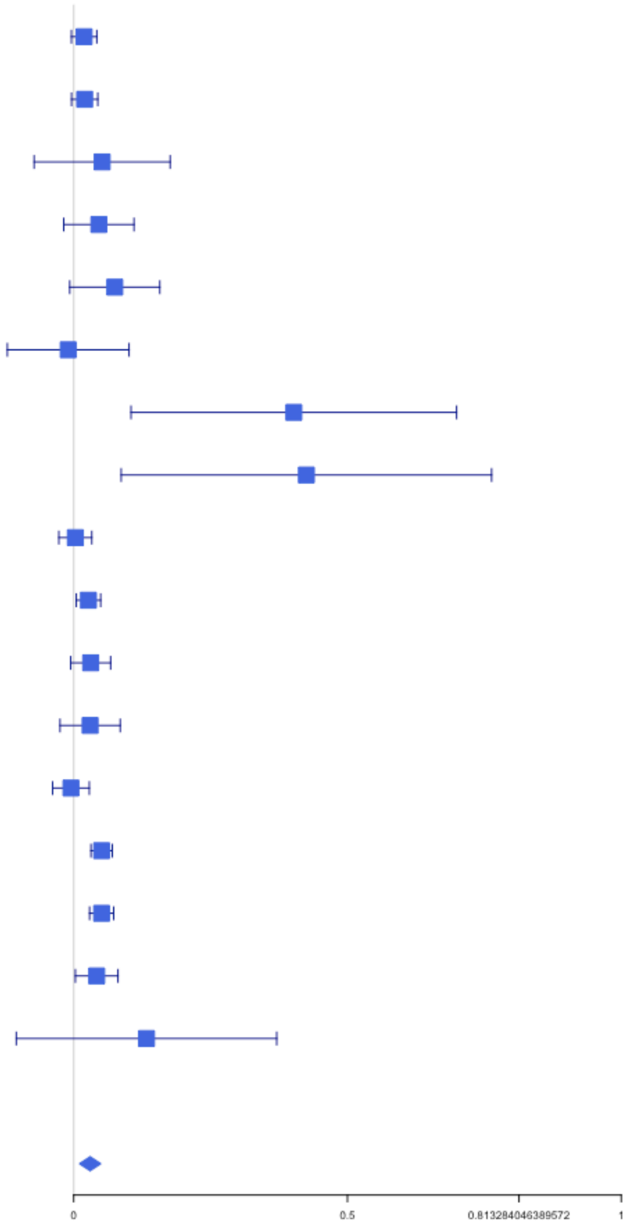

q) **FEV_1/FVC:** Population- and family-based, African American; rs145829100 (chr13:84,795,360)

Study	n	Effect Size
ARIC_all	3256	-6.512
ARIC_white	3075	5.514
ARIC_black*	181	-12.648
CFS_all	719	-1.945
CFS_white	373	
CFS_black*	346	-2.661
CHS_all	47	
CHS_white	39	
FHS_all	1835	
MESA_all	3252	-5.112
MESA_white	1224	
MESA_black*	804	-5.866
JHS_all*	2388	-5.091
COPDGene_all	8444	-2.042
COPDGene_white	5713	-3.947
COPDGene_black	2731	-1.499
EOCOPD_all	55	
**Summary**	**19996**	**-5.18**

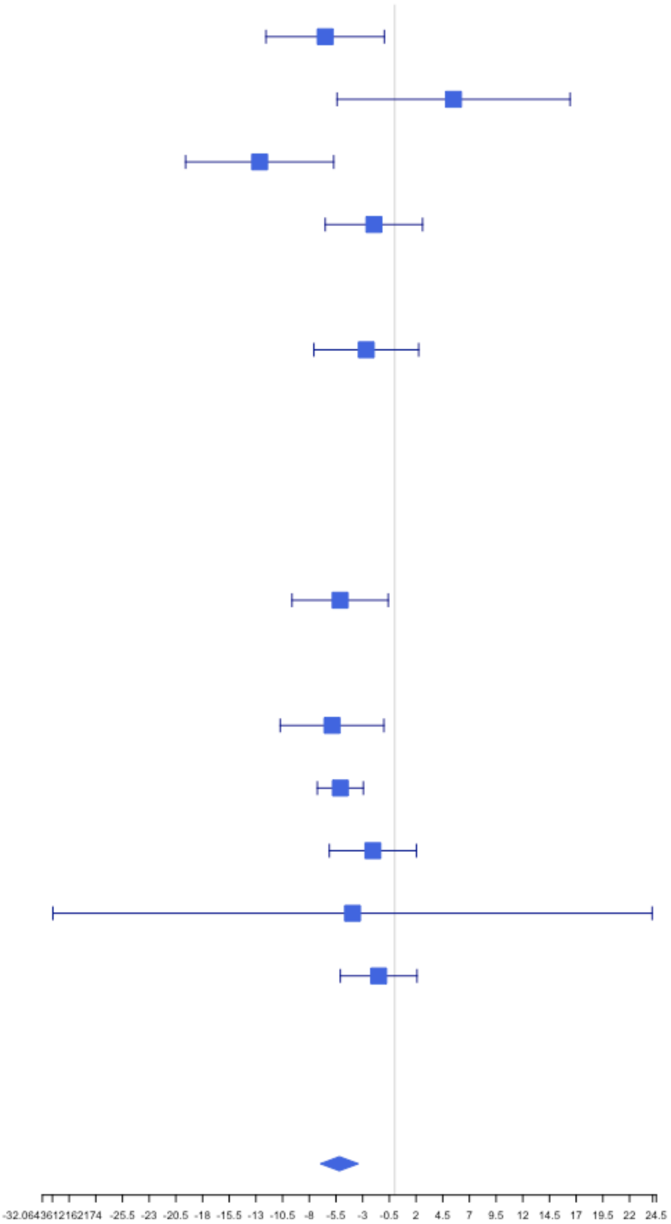

r) **FEV_1/FVC:** Population- and family-based, White; rs544345041 (chr7:30,969,275)

Study	n	Effect Size
ARIC_all	3256	8.518
ARIC_white*	3075	8.636
ARIC_black	181	
CFS_all	719	3.487
CFS_white*	373	5.008
CFS_black	346	
CHS_all	47	-18.174
CHS_white*	39	-19.428
FHS_all*	1835	6.216
MESA_all	3252	0.393
MESA_white*	1224	2.765
MESA_black	804	
JHS_all	2388	
COPDGene_all	8444	4.625
COPDGene_white	5713	4.403
COPDGene_black	2731	4.108
EOCOPD_all	55	-8.682
**Summary**	**19996**	**6.61**

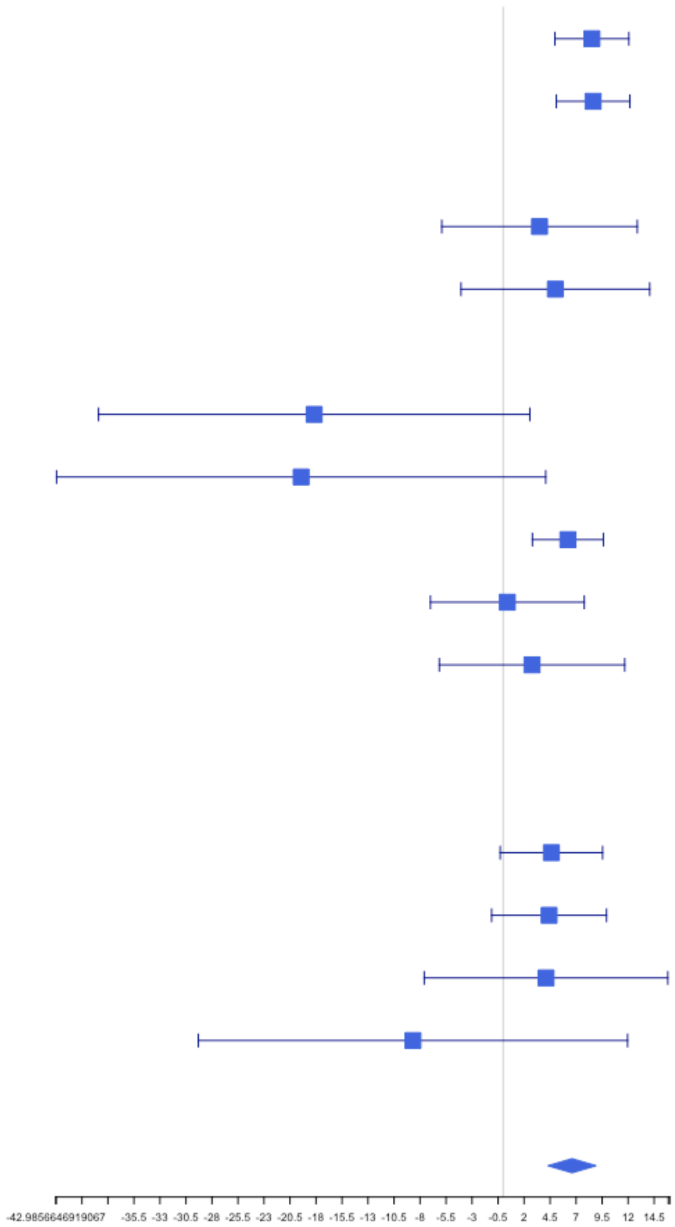

s) **FEV$_1$/FVC:** COPD-enriched, African American; rs144870669 (chr8:133,793,876)

Study	n	Effect Size
ARIC_all	3256	0.201
ARIC_white	3075	
ARIC_black	181	0.961
CFS_all	719	-0.677
CFS_white	373	
CFS_black	346	-1.193
CHS_all	47	
CHS_white	39	
FHS_all	1835	
MESA_all	3252	-2.725
MESA_white	1224	
MESA_black	804	-4.995
JHS_all	2388	1.096
COPDGene_all	8444	10.097
COPDGene_white	5713	
COPDGene_black*	2731	0.825
EOCOPD_all	55	
**Summary**	**19996**	**0.83**

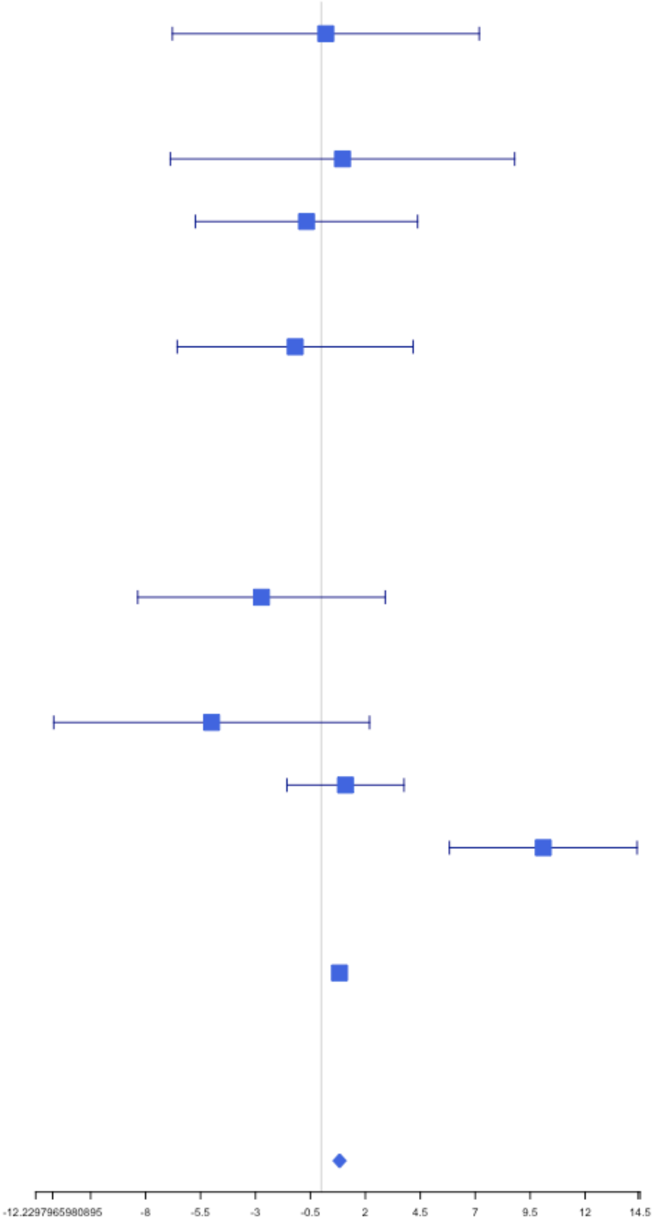

t) **FEV_1/FVC:** Combined, African American; rs1032155362 (chr6:107,949,366)

Study	n	Effect Size
ARIC_all	3256	-16.179
ARIC_white	3075	
ARIC_black*	181	-17.317
CFS_all	719	
CFS_white	373	
CFS_black*	346	
CHS_all	47	
CHS_white	39	
FHS_all	1835	
MESA_all	3252	-6.61
MESA_white	1224	
MESA_black*	804	-6.273
JHS_all*	2388	-6.614
COPDGene_all	8444	-11.757
COPDGene_white	5713	
COPDGene_black*	2731	-12.03
EOCOPD_all	55	
**Summary**	**19996**	**-10.44**

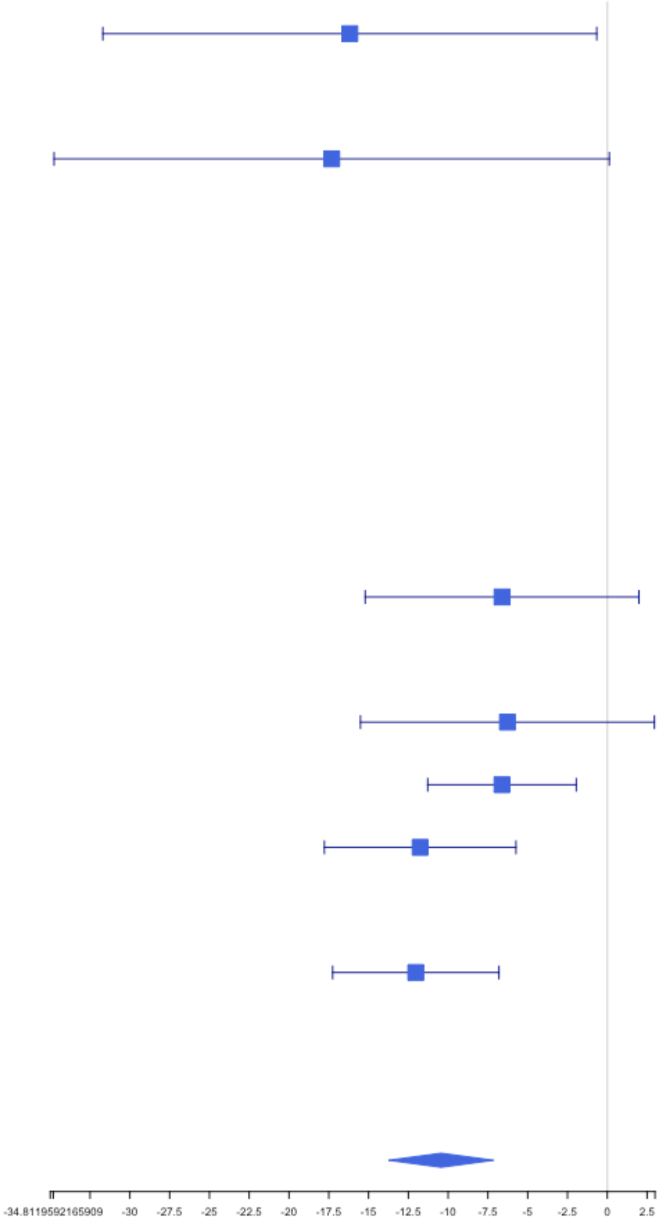

u) **FEV$_1$/FVC:** Combined, White; rs572153283 (chr6:102,490,266)

Study	n	Effect Size
ARIC_all	3256	-2.775
ARIC_white*	3075	-2.801
ARIC_black	181	
CFS_all	719	6.284
CFS_white*	373	6.626
CFS_black	346	
CHS_all	47	
CHS_white*	39	
FHS_all*	1835	-1.147
MESA_all	3252	-1.505
MESA_white*	1224	-1.031
MESA_black	804	-4.201
JHS_all	2388	-0.048
COPDGene_all	8444	-4.625
COPDGene_white*	5713	-5.384
COPDGene_black	2731	1.718
EOCOPD_all*	55	-0.628
**Summary**	**19996**	**-3.57**

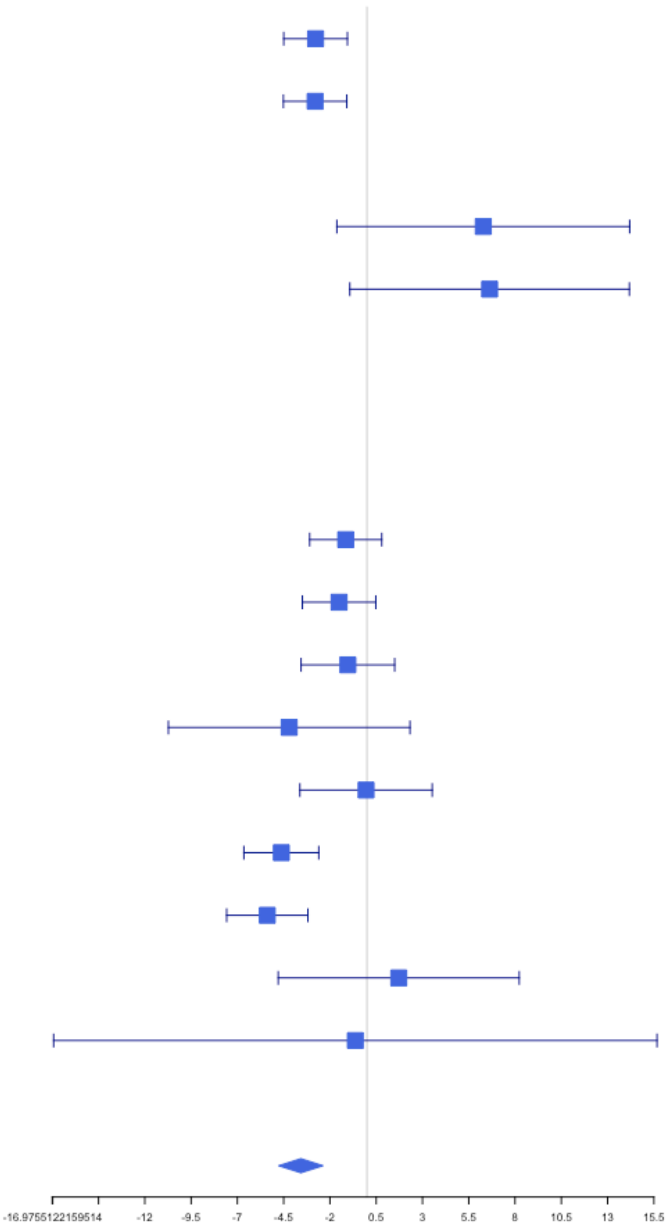

v) **FEV_1/FVC:** Combined, White; rs7188378 (chr16:53,872,940)

Study	n	Effect Size
ARIC_all	3256	-0.256
ARIC_white*	3075	-0.306
ARIC_black	181	0.578
CFS_all	719	0.333
CFS_white*	373	0.094
CFS_black	346	0.646
CHS_all	47	2.712
CHS_white*	39	1.226
FHS_all*	1835	-0.145
MESA_all	3252	-0.224
MESA_white*	1224	-0.558
MESA_black	804	-0.02
JHS_all	2388	-0.39
COPDGene_all	8444	-1.075
COPDGene_white*	5713	-1.356
COPDGene_black	2731	-0.443
EOCOPD_all*	55	-2.19
**Summary**	**19996**	**-0.79**

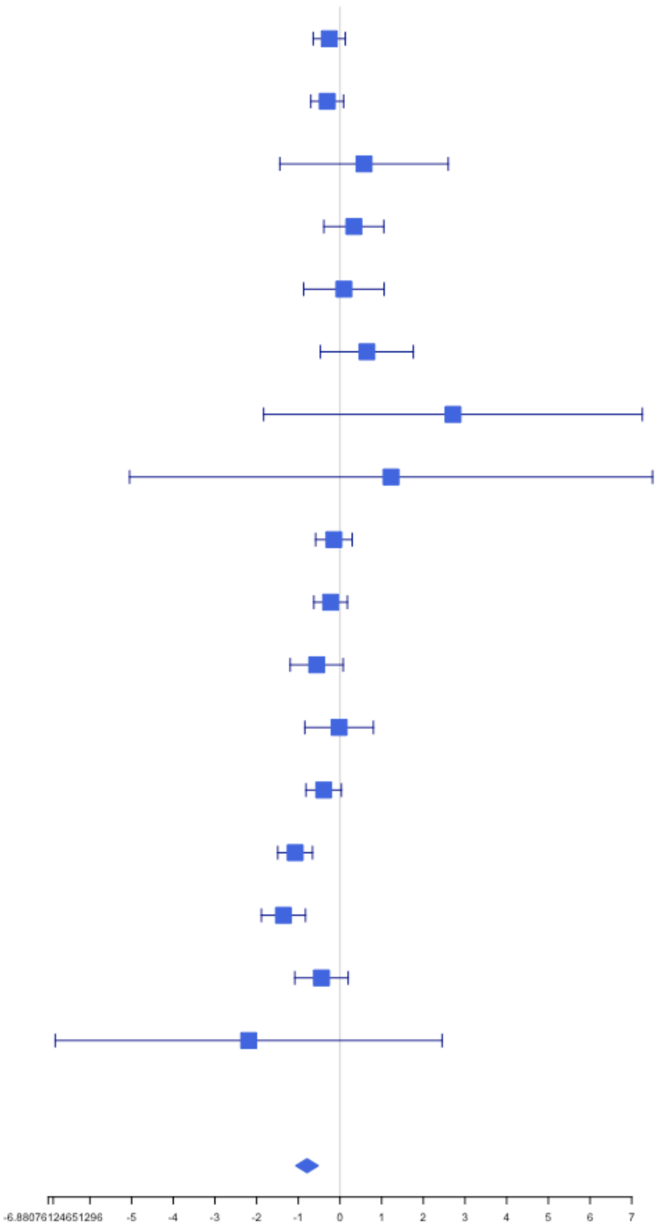

w) **FEV_1/FVC:** Combined, All; rs137938609 (chr6:102,543,833)

Study	n	Effect Size
ARIC_all*	3256	-2.904
ARIC_white	3075	-2.929
ARIC_black	181	
CFS_all*	719	4.635
CFS_white	373	4.449
CFS_black	346	
CHS_all*	47	
CHS_white	39	
FHS_all*	1835	-1.276
MESA_all*	3252	-1.344
MESA_white	1224	-0.59
MESA_black	804	-7.774
JHS_all*	2388	-1.387
COPDGene_all*	8444	-4.691
COPDGene_white	5713	-5.519
COPDGene_black	2731	2.737
EOCOPD_all*	55	-0.628
**Summary**	**19996**	**-3.31**

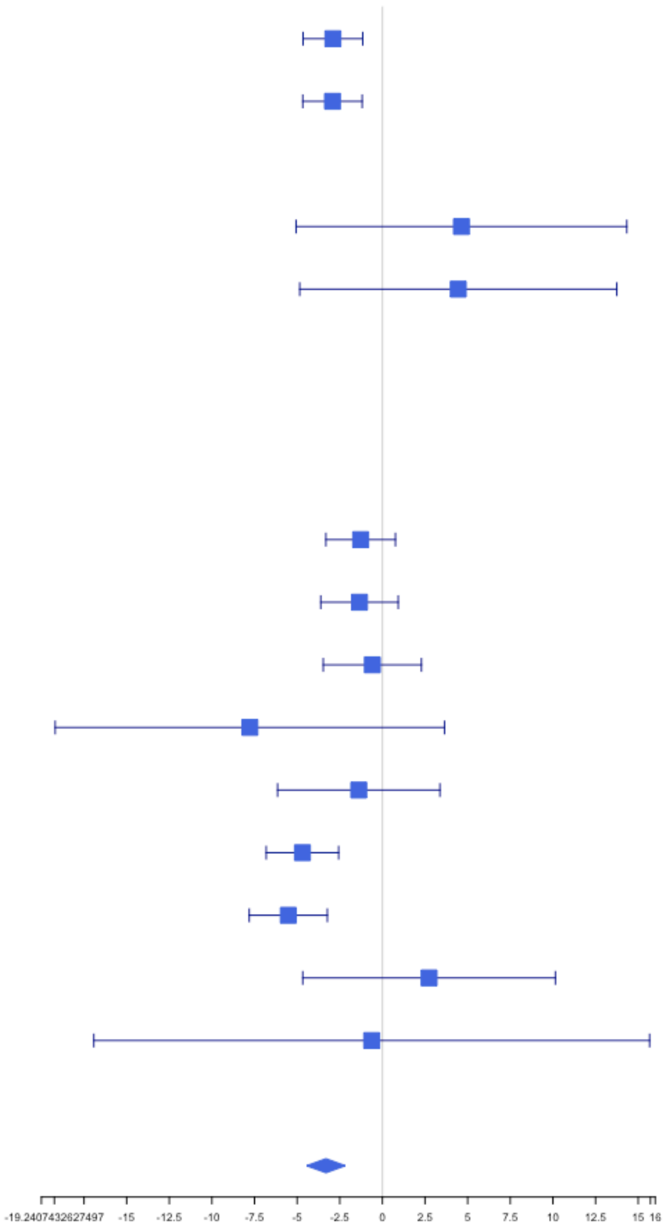

x) **FEV_1/FVC:** Combined, All; rs184101688 (chr7:7,140,556)

Study	n	Effect Size
ARIC_all*	3256	-5.211
ARIC_white	3075	-6.327
ARIC_black	181	1.183
CFS_all*	719	-5.08
CFS_white	373	-5.575
CFS_black	346	
CHS_all*	47	
CHS_white	39	
FHS_all*	1835	-0.311
MESA_all*	3252	-8.921
MESA_white	1224	-13.875
MESA_black	804	-7.403
JHS_all*	2388	
COPDGene_all*	8444	-16.156
COPDGene_white	5713	-18.221
COPDGene_black	2731	-11.5
EOCOPD_all*	55	
**Summary**	**19996**	**-9.42**

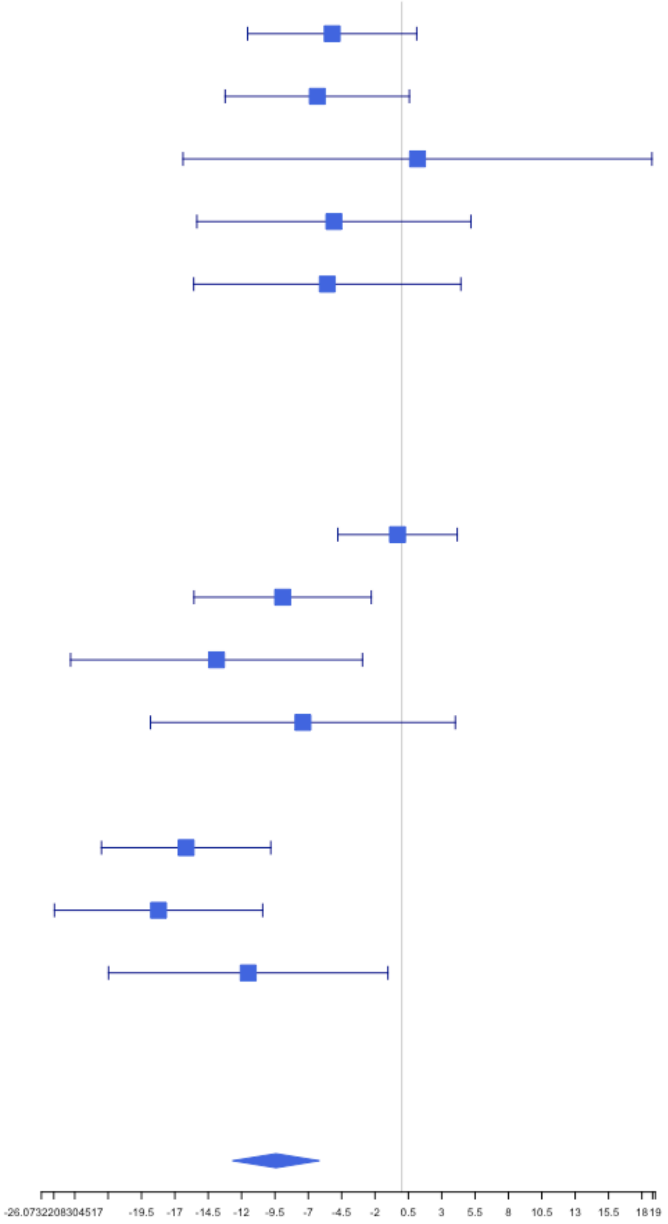

y) **FEV_1/FVC:** Combined, All; rs7188378 (chr16:53,872,940)

Study	n	Effect Size
ARIC_all*	3256	-0.256
ARIC_white	3075	-0.306
ARIC_black	181	0.578
CFS_all*	719	0.333
CFS_white	373	0.094
CFS_black	346	0.646
CHS_all*	47	2.712
CHS_white	39	1.226
FHS_all*	1835	-0.145
MESA_all*	3252	-0.224
MESA_white	1224	-0.558
MESA_black	804	-0.02
JHS_all*	2388	-0.39
COPDGene_all*	8444	-1.075
COPDGene_white	5713	-1.356
COPDGene_black	2731	-0.443
EOCOPD_all*	55	-2.19
**Summary**	**19996**	**-0.59**

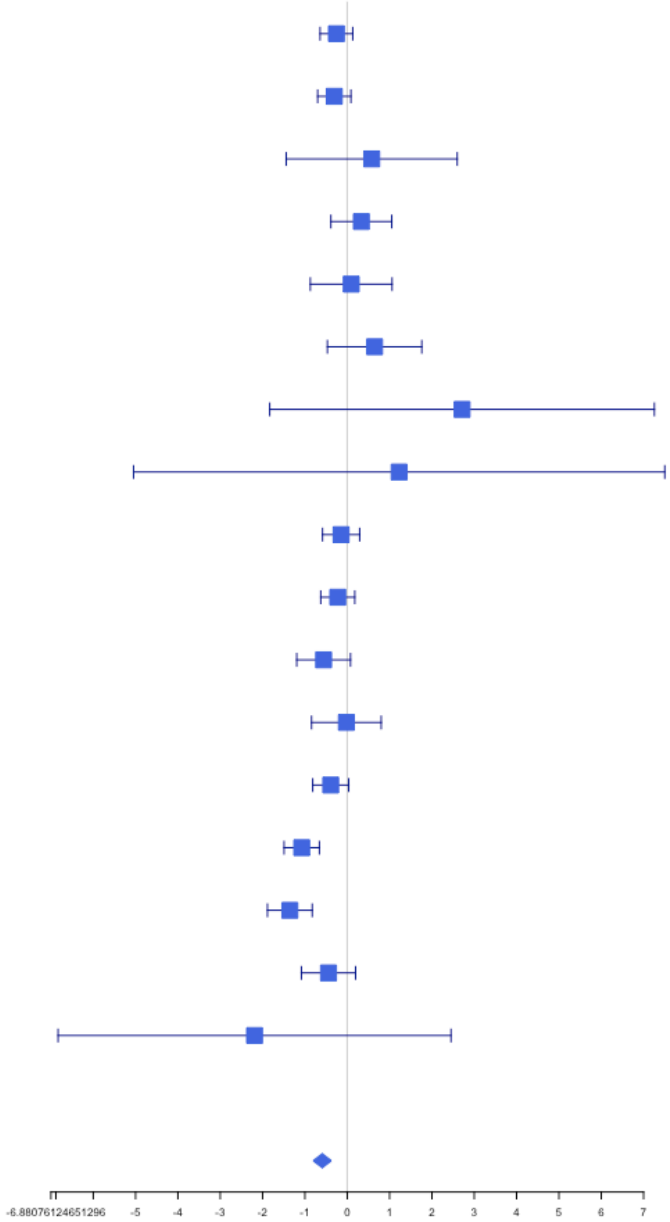

z) **Moderate-to-Severe COPD:** COPD-enriched, All; rs72740913 (chr15:53,390,153)

Study	n	OR
ARIC_all	3256	0.478
ARIC_white	3075	0.48
ARIC_black	181	
CFS_all	719	2.413
CFS_white	373	3.939
CFS_black	346	
CHS_all	47	
CHS_white	39	
FHS_all	1835	1.397
MESA_all	3252	0.885
MESA_white	1224	0.854
MESA_black	804	
JHS_all	2388	
COPDGene_all*	8444	2.965
COPDGene_white	5713	3.065
COPDGene_black	2731	2.01
EOCOPD_all*	55	
**Summary**	**19996**	**2.945**

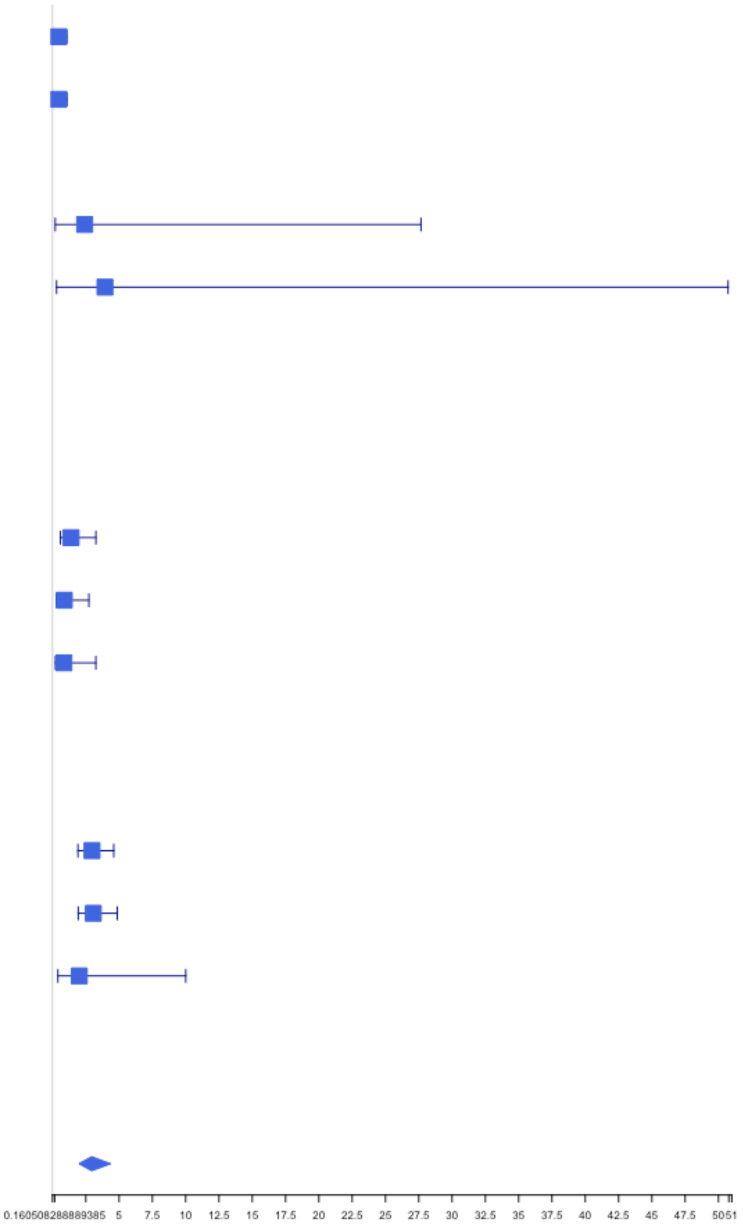

aa)**Severe COPD:** Combined, White; rs11687134 (chr2:141,400,617)

Study	n	OR
ARIC_all	3256	0.58
ARIC_white*	3075	0.607
ARIC_black	181	
CFS_all	719	1.19
CFS_white*	373	
CFS_black	346	
CHS_all	47	
CHS_white*	39	
FHS_all*	1835	0.52
MESA_all	3252	0.908
MESA_white*	1224	1.026
MESA_black	804	0.723
JHS_all	2388	1.11
COPDGene_all	8444	0.809
COPDGene_white*	5713	0.77
COPDGene_black	2731	0.974
EOCOPD_all*	55	
**Summary**	**19996**	**0.712**

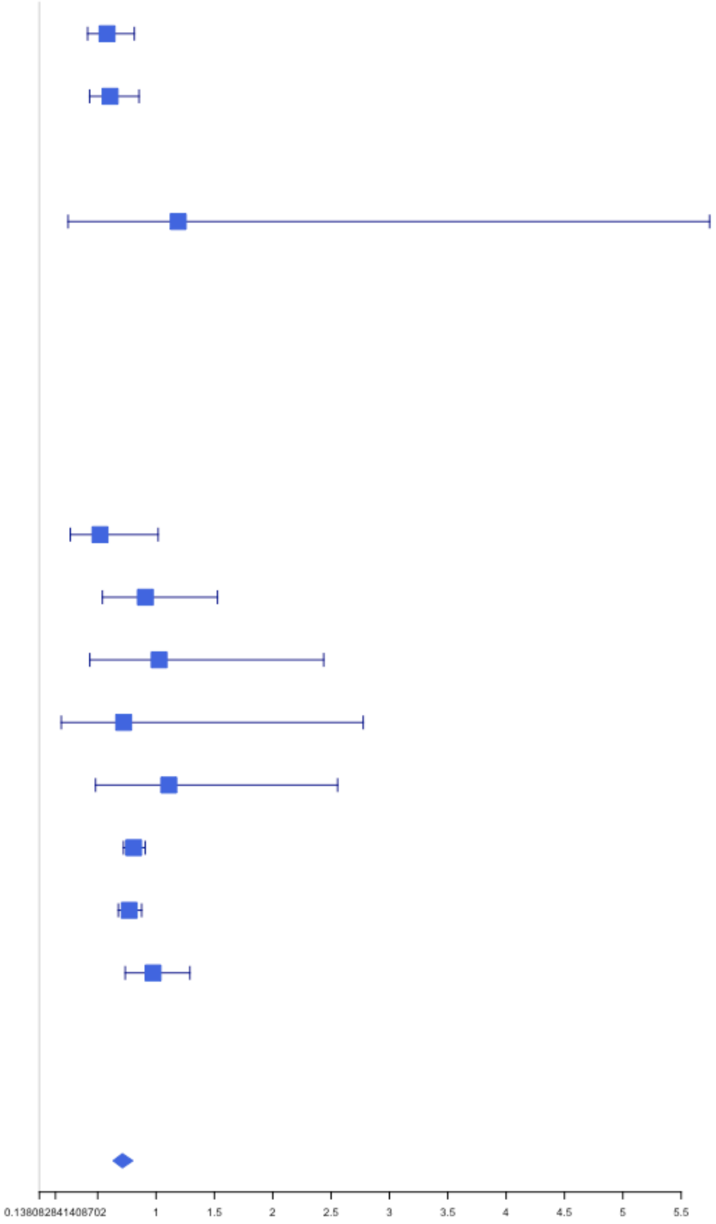

Supplementary Figure 4: Power of quantitative trait analysis in replication analysis. Power is shown across a range of values for the genetic additive effect in units of trait standard deviations, as indicated on the figure legends. The sample sizes shown correspond to replication analysis in the UK Biobank European ancestry (n=320,000), HCHS/SOL (n=11,800) and the UK Biobank African Ancestry(n=4350) samples.. On the x-axis, “MAF” denotes minor allele frequency. Power calculations were carried out using QUANTO.[30] Supplementary Figure 4a presents power assuming a nominal significance threshold of *P*=0.05, while Supplementary Figure 4b shows power assuming a significance threshold of *P*=0.005 (corresponding roughly to Bonferroni correction for the number of genetic variants examined for replication with each trait).

a) Power assuming a nominal significance threshold of *P*=0.05.

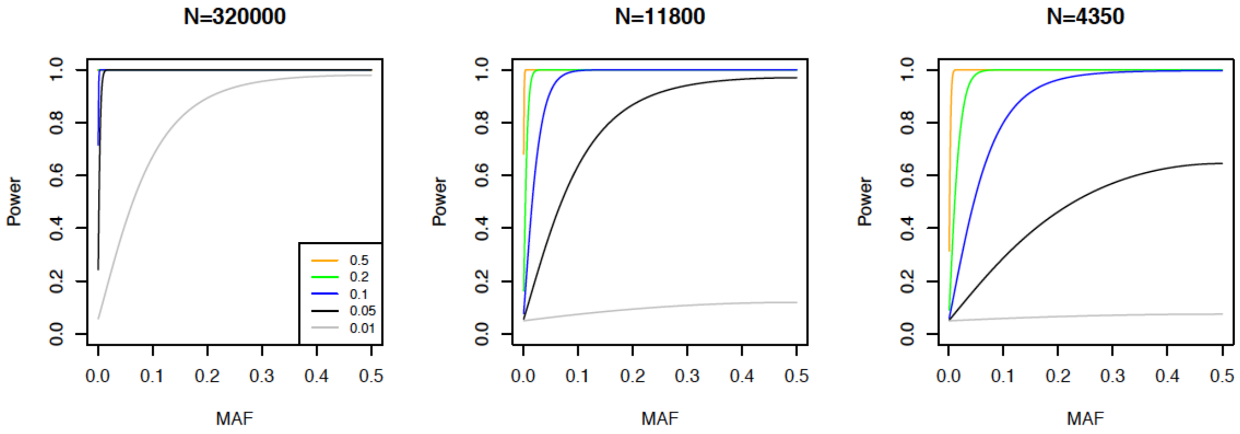

b) Power assuming a significance threshold of *P*=0.005.

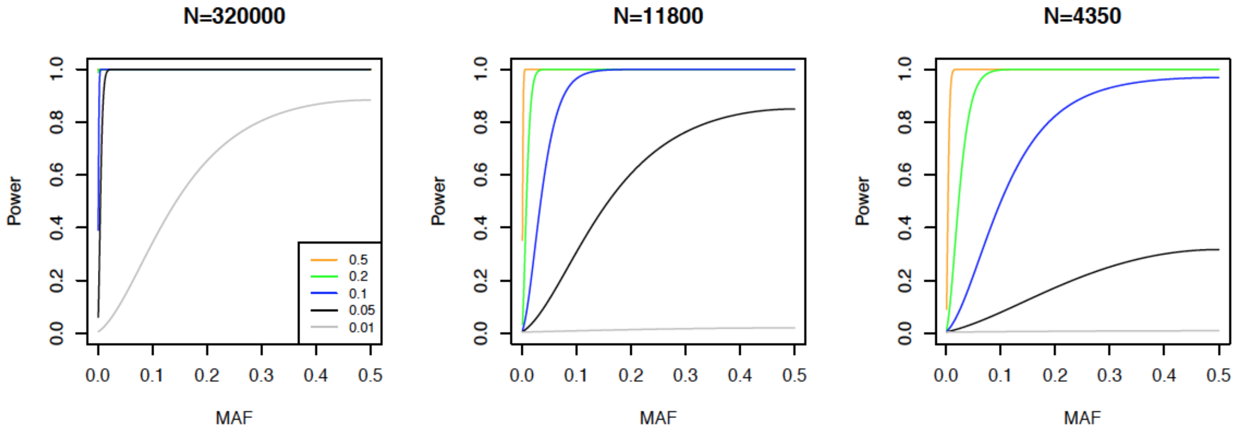

Supplementary Figure 5: Bayesian colocalization plot for the variant rs12556310 associated with FVC. The upper plot shows the TOPMed WGS results for FVC based on analysis of COPD-enriched samples in all race/ethnicities. The lower plot shows the corresponding local genetic association results for expression of *RGN* based on expression in GTEx v7 lung.

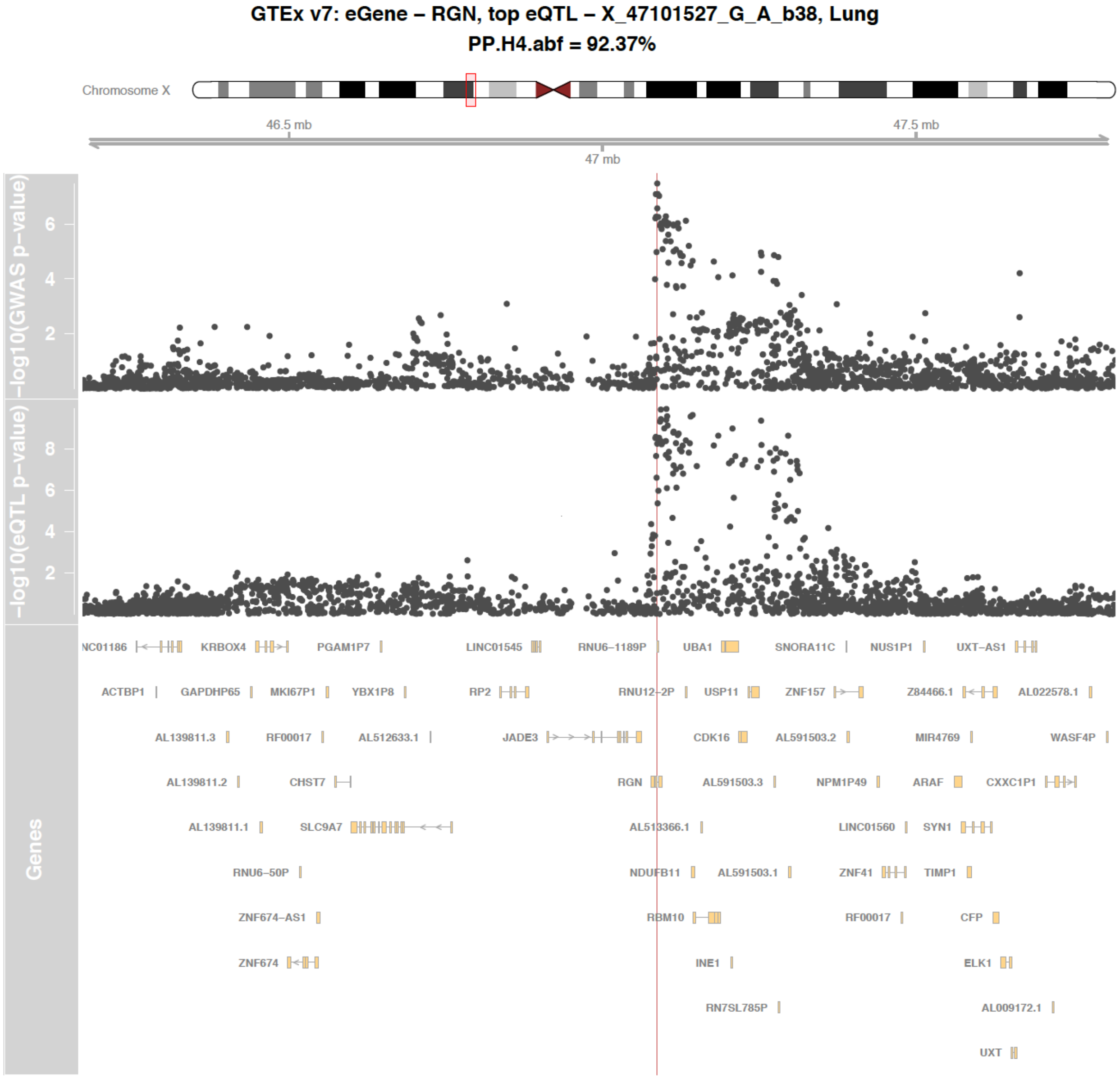

Supplementary Figure 6: Bayesian colocalization plot for the signal at rs9295345, identified in WGS of FEV_1. The upper plot shows the TOPMed WGS results for FEV_1 based on analysis of combined White samples. The lower plot shows the corresponding local genetic association results for methylation levels at cg06249499 from MESA Exam 1. Note that colocalization was observed in comparing WGS results from combined White samples with genetic association for methylation in whole blood for the same site cg06249499 at both MESA Exams 1 and 5. Here, we focus on the results from MESA Exam 1 for the purpose of display.

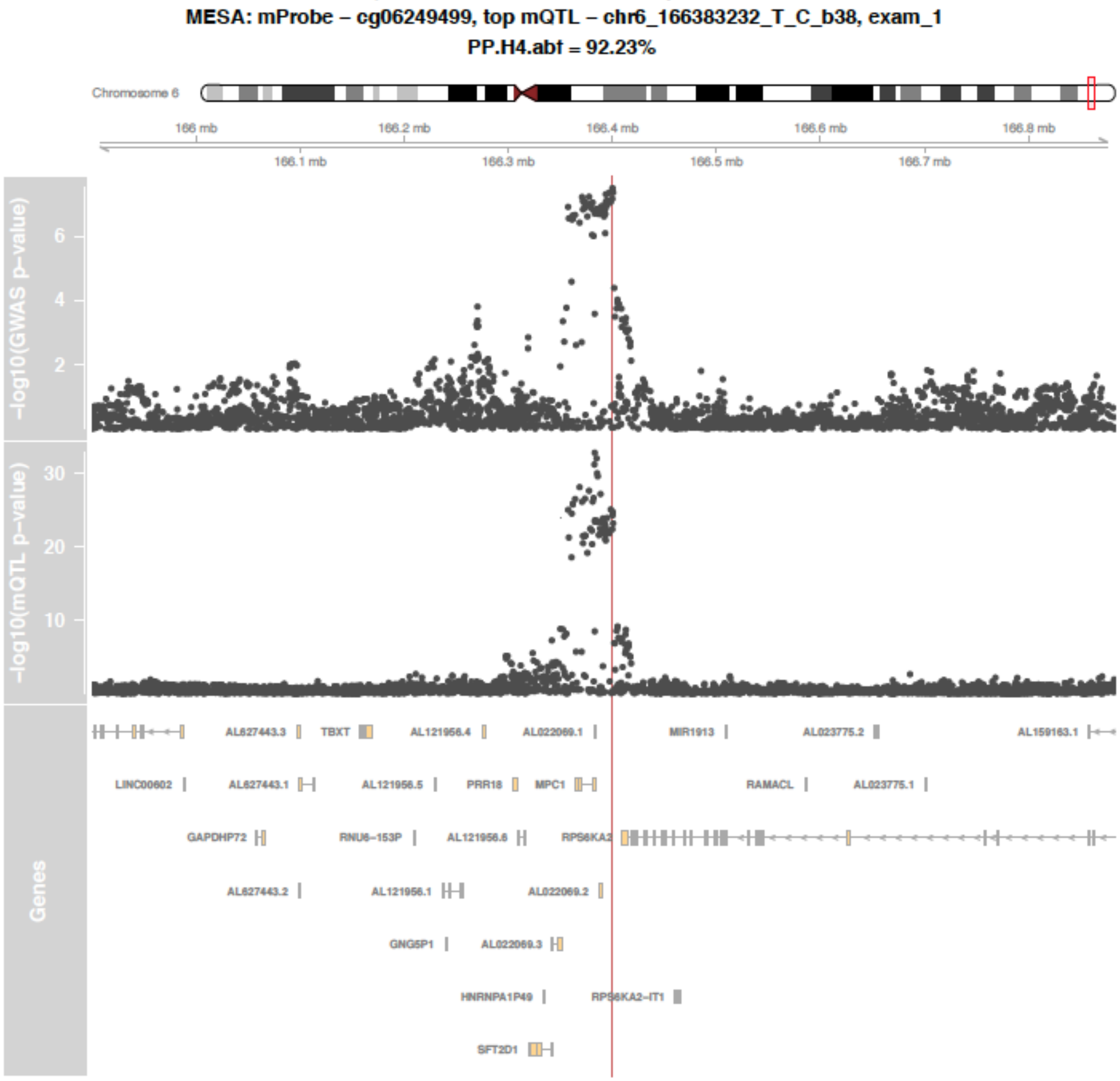

Supplementary References

1. Oelsner, E. C. *et al.* Harmonization of Respiratory Data From 9 US Population-Based Cohorts: The NHLBI Pooled Cohorts Study. *Am. J. Epidemiol.* **187**, 2265–2278 (2018).
2. The Atherosclerosis Risk in Communities (ARIC) Study: design and objectives. The ARIC investigators. *Am. J. Epidemiol.* **129**, 687–702 (1989).
3. Mirabelli, M. C. *et al.* Lung function decline over 25 years of follow-up among black and white adults in the ARIC study cohort. *Respir Med* **113**, 57–64 (2016).
4. Fried, L. P. *et al.* The Cardiovascular Health Study: design and rationale. *Ann Epidemiol* **1**, 263–276 (1991).
5. Enright, P. L., Kronmal, R. A., Higgins, M., Schenker, M. & Haponik, E. F. Spirometry reference values for women and men 65 to 85 years of age. Cardiovascular health study. *Am. Rev. Respir. Dis.* **147**, 125–133 (1993).
6. Enright, P. L., Kronmal, R. A., Higgins, M. W., Schenker, M. B. & Haponik, E. F. Prevalence and correlates of respiratory symptoms and disease in the elderly. Cardiovascular Health Study. *Chest* **106**, 827–834 (1994).
7. Larkin, E. K. *et al.* A candidate gene study of obstructive sleep apnea in European Americans and African Americans. *Am. J. Respir. Crit. Care Med.* **182**, 947–953 (2010).
8. Kannel, W. B., Feinleib, M., McNamara, P. M., Garrison, R. J. & Castelli, W. P. An Investigation of Coronary Heart Disease in Families: The Framingham Offspring Study. *Am. J. Epidemiol.* **185**, 1093–1102 (2017).
9. Taylor, H. A. The Jackson Heart Study: an overview. *Ethn Dis* **15**, S6-1–3 (2005).
10. Taylor, H. A. *et al.* Toward resolution of cardiovascular health disparities in African Americans: design and methods of the Jackson Heart Study. *Ethn Dis* **15**, S6-4–17 (2005).
11. Wilson, J. G. *et al.* Study design for genetic analysis in the Jackson Heart Study. *Ethn Dis* **15**, S6-30–37 (2005).
12. Bild, D. E. *et al.* Multi-Ethnic Study of Atherosclerosis: objectives and design. *Am. J.*

Epidemiol. **156**, 871–881 (2002).

13. Hankinson, J. L. *et al.* Performance of American Thoracic Society-recommended spirometry reference values in a multiethnic sample of adults: the multi-ethnic study of atherosclerosis (MESA) lung study. *Chest* **137**, 138–145 (2010).

14. Silverman, E. K. *et al.* Genomewide linkage analysis of quantitative spirometric phenotypes in severe early-onset chronic obstructive pulmonary disease. *Am. J. Hum. Genet.* **70**, 1229–1239 (2002).

15. Regan, E. A. *et al.* Genetic epidemiology of COPD (COPDGene) study design. *COPD* **7**, 32–43 (2010).

16. 1000 Genomes Project Consortium *et al.* An integrated map of genetic variation from 1,092 human genomes. *Nature* **491**, 56–65 (2012).

17. 1000 Genomes Project Consortium *et al.* A global reference for human genetic variation. *Nature* **526**, 68–74 (2015).

18. McCarthy, S. *et al.* A reference panel of 64,976 haplotypes for genotype imputation. *Nat. Genet.* **48**, 1279–1283 (2016).

19. Bycroft, C. *et al.* The UK Biobank resource with deep phenotyping and genomic data. *Nature* **562**, 203–209 (2018).

20. Shrine, N. *et al.* New genetic signals for lung function highlight pathways and chronic obstructive pulmonary disease associations across multiple ancestries. *Nat. Genet.* **51**, 481–493 (2019).

21. Loh, P.-R. *et al.* Efficient Bayesian mixed-model analysis increases association power in large cohorts. *Nat. Genet.* **47**, 284–290 (2015).

22. Barr, R. G. *et al.* Pulmonary Disease and Age at Immigration among Hispanics. Results from the Hispanic Community Health Study/Study of Latinos. *Am. J. Respir. Crit. Care Med.* **193**, 386–395 (2016).

23. Lavange, L. M. *et al.* Sample design and cohort selection in the Hispanic Community

Health Study/Study of Latinos. *Ann Epidemiol* **20**, 642–649 (2010).

24. Sorlie, P. D. *et al.* Design and implementation of the Hispanic Community Health Study/Study of Latinos. *Ann Epidemiol* **20**, 629–641 (2010).

25. Burkart, K. M. *et al.* A Genome-Wide Association Study in Hispanics/Latinos Identifies Novel Signals for Lung Function. The Hispanic Community Health Study/Study of Latinos. *Am. J. Respir. Crit. Care Med.* **198**, 208–219 (2018).

26. Sofer, T. *et al.* Meta-Analysis of Genome-Wide Association Studies with Correlated Individuals: Application to the Hispanic Community Health Study/Study of Latinos (HCHS/SOL). *Genet. Epidemiol.* **40**, 492–501 (2016).

27. Taylor-Weiner, A. *et al.* Scaling computational genomics to millions of individuals with GPUs. *Genome Biol.* **20**, 228 (2019).

28. Stegle, O., Parts, L., Durbin, R. & Winn, J. A Bayesian Framework to Account for Complex Non-Genetic Factors in Gene Expression Levels Greatly Increases Power in eQTL Studies. *PLOS Computational Biology* **6**, e1000770 (2010).

29. Giambartolomei, C. *et al.* Bayesian test for colocalisation between pairs of genetic association studies using summary statistics. *PLoS Genet.* **10**, e1004383 (2014).

30. Gauderman, W. J. Sample size requirements for matched case-control studies of gene-environment interaction. *Stat Med* **21**, 35–50 (2002).

31. Zhou, W. *et al.* Efficiently controlling for case-control imbalance and sample relatedness in large-scale genetic association studies. *Nat. Genet.* **50**, 1335–1341 (2018).
