# Supplementary material for: Whole genome sequence analysis of pulmonary function and COPD in 19,996 multi-ethnic participants
Source: Nat Commun. 2020 Oct 14;11:5182. doi: 10.1038/s41467-020-18334-7 (PMC7598941; doi:10.1038/s41467-020-18334-7)
Supplement: Supplementary file 3 — Descriptions of Additional Supplementary Files [file 41467_2020_18334_MOESM3_ESM.pdf]

Descriptions of Additional Supplementary Files

Supplementary Data 1

Description: Detailed cohort-specific descriptive characteristics.

Supplementary Data 2

Description: Detailed list of genome-wide significant variants, including those identified in previously published studies.

Supplementary Data 3

Description: Summary of association results in TOPMed for selected variants reported previously in association with lung function and COPD.

Supplementary Data 4

Description: Detailed results of genetic association with FEV1 in TOPMed for previously reported variants.

Supplementary Data 5

Description: Detailed results of genetic association with FVC in TOPMed for previously reported variants.

Supplementary Data 6

Description: Detailed results of genetic association with FEV1/FVC ratio in TOPMed for previously reported variants.

Supplementary Data 7

Description: Detailed results of genetic association with moderate-to-severe COPD in TOPMed for previously reported variants.

Supplementary Data 8

Description: Detailed results of genetic association with severe COPD in TOPMed for previously reported variants.

Supplementary Data 9

Description: Sex-stratified analysis of the novel variants on the X chromosome.

Supplementary Data 10

Description: Annotation of novel variants.

Supplementary Data 11

Description: Summary of significant results from conditional association analysis.

Supplementary Data 12

Description: Correspondence (R-squared) of whole genome sequence calls with imputed genotypes in MESA.

Supplementary Data 13

Description: Gene-based analysis of pLOF variants.

Supplementary Data 14

Description: Examination of association of novel variants with measures of pulmonary function in n=321,047 European ancestry samples from the UK Biobank.

Supplementary Data 15

Description: Examination of association of novel variants with measures of smoking behavior traits in up to n=447,062 European ancestry samples from the UK Biobank.

Supplementary Data 16

Description: Examination of association of novel variants with measures of pulmonary function in n=4,350 African ancestry samples from the UK Biobank.

Supplementary Data 17

Description: Examination of association of novel variants with measures of smoking behavior traits in up to n=7,702 African ancestry samples from the UK Biobank.

Supplementary Data 18

Description: Examination of association of novel variants with measures of pulmonary function in n=11,822 self-reported Hispanic individuals from HCHS/SOL.

Supplementary Data 19

Description: Phenome-wide association results of novel signals with 1,403 binary traits in White British participants from the UK Biobank.

Supplementary Data 20

Description: Summary of Bayesian colocalization analysis for eQTL based on 48 tissues in GTEx v7.

Supplementary Data 21

Description: Summary of Bayesian colocalization analysis results for mQTL based on genome-wide methylation in MESA whole blood.

Supplementary Data 22

Description: Overlap of selected genes with GO terms previously implicated by GWAS.

Supplementary Data 23

Description: GWAS candidate genes for pLOF analysis of FEV1.

Supplementary Data 24

Description: GWAS candidate genes for pLOF analysis of FVC.

Supplementary Data 25

Description: GWAS candidate genes for pLOF analysis of FEV1/FVC.

Supplementary Data 26

Description: GWAS candidate genes for pLOF analysis of COPD.
