# Supplementary material for: Whole genome sequence analysis of pulmonary function and COPD in 19,996 multi-ethnic participants
Source: Nat Commun. 2020 Oct 14;11:5182. doi: 10.1038/s41467-020-18334-7 (PMC7598941; doi:10.1038/s41467-020-18334-7)
Supplement: Supplementary file 30 — Reporting Summary [file 41467_2020_18334_MOESM30_ESM.pdf]

nature research

Corresponding author(s): Michael H. Cho
Ani Manichaikul

Last updated by author(s): Jul 21, 2020

Reporting Summary

Nature Research wishes to improve the reproducibility of the work that we publish. This form provides structure for consistency and transparency in reporting. For further information on Nature Research policies, see Authors & Referees and the Editorial Policy Checklist .

Statistics

For all statistical analyses, confirm that the following items are present in the figure legend, table legend, main text, or Methods section.

n/a	Confirmed	
☐	☒	The exact sample size (*n*) for each experimental group/condition, given as a discrete number and unit of measurement
☐	☒	A statement on whether measurements were taken from distinct samples or whether the same sample was measured repeatedly
☐	☒	The statistical test(s) used AND whether they are one- or two-sided<br>*Only common tests should be described solely by name; describe more complex techniques in the Methods section.*
☐	☒	A description of all covariates tested
☐	☒	A description of any assumptions or corrections, such as tests of normality and adjustment for multiple comparisons
☐	☒	A full description of the statistical parameters including central tendency (e.g. means) or other basic estimates (e.g. regression coefficient) AND variation (e.g. standard deviation) or associated estimates of uncertainty (e.g. confidence intervals)
☐	☒	For null hypothesis testing, the test statistic (e.g. *F*, *t*, *r*) with confidence intervals, effect sizes, degrees of freedom and *P* value noted<br>*Give P values as exact values whenever suitable.*
☐	☒	For Bayesian analysis, information on the choice of priors and Markov chain Monte Carlo settings
☒	☐	For hierarchical and complex designs, identification of the appropriate level for tests and full reporting of outcomes
☐	☒	Estimates of effect sizes (e.g. Cohen's *d*, Pearson's *r*), indicating how they were calculated

Our web collection on statistics for biologists contains articles on many of the points above.

Software and code

Policy information about availability of computer code

Data collection	Methods for preparation of the TOPMed Whole Genome Sequence data have been described previously in Taliun et al. Sequencing of 53,831 diverse genomes from the NHLBI TOPMed Program | bioRxiv. https://www.biorxiv.org/content/10.1101/563866v1.<br><br>Methods for preparation and harmonization of pulmonary traits were described previously in Oelsner et al. Harmonization of Respiratory Data From 9 US Population-Based Cohorts: The NHLBI Pooled Cohorts Study. Am. J. Epidemiol. 187, 2265–2278 (2018).<br><br>No software was used for data collection as part of the current study.
Data analysis	- We used R v3.5.2 and the R package GENESIS v2.12.2 to prepare phenotypes for genetic association analysis, including data cleaning and inverse normal transform.<br>- We used SAIGE-LMM v0.29.4.4 to perform single variant analysis.<br>- We used GCTA-COJO v1.93.2 to perform conditional analysis.<br>- We used SAIGE-GENE v0.36.3.3.to perform gene -based analysis.<br>- We used WGS Annotator (WGSA) v0.7 to annotate variants as part of the current study.<br>- We used the R/coloc v3.1 package to carry out colocalization of eQTL and mQTL.

For manuscripts utilizing custom algorithms or software that are central to the research but not yet described in published literature, software must be made available to editors/reviewers. We strongly encourage code deposition in a community repository (e.g. GitHub). See the Nature Research guidelines for submitting code & software for further information.

Data

Policy information about availability of data

All manuscripts must include a data availability statement. This statement should provide the following information, where applicable:

- Accession codes, unique identifiers, or web links for publicly available datasets
- A list of figures that have associated raw data
- A description of any restrictions on data availability

All of the TOPMed Whole Genome Sequence data are available on dbGaP. The dbGaP accession numbers for each study are provided in our Data Availability statement.

Field-specific reporting

Please select the one below that is the best fit for your research. If you are not sure, read the appropriate sections before making your selection.

☒ Life sciences ☐ Behavioural & social sciences ☐ Ecological, evolutionary & environmental sciences

For a reference copy of the document with all sections, see nature.com/documents/nr-reporting-summary-flat.pdf

Life sciences study design

All studies must disclose on these points even when the disclosure is negative.

Sample size	We did not perform a sample size calculation to design the study. We pooled together all available samples from TOPMed Freeze 5b and included in our study all of those samples with phenotypes available for lung function traits (FEV1, FVC, FEV1/FVC ratio) or COPD.
Data exclusions	For case-control genetic association analysis of COPD, those participants who did not meet the strict definition of COPD case or control were excluded from analyses.
Replication	For those variants demonstrating novel associations with one or more measures of pulmonary function or COPD, we examined evidence of replication in the UK Biobank and the Hispanic Community Health Study / Study of Latinos (HCHS/SOL). Only variants passing quality control and other filters for analyses in the respective replication cohorts were considered when we performed multiple comparisons corrections to determine which variants demonstrated evidence of replication.
Randomization	Randomization was not relevant to our study since the research was carried out as an observational study.
Blinding	Blinding was not relevant to our study since there was no random assignment of participants to particular groups.

Reporting for specific materials, systems and methods

We require information from authors about some types of materials, experimental systems and methods used in many studies. Here, indicate whether each material, system or method listed is relevant to your study. If you are not sure if a list item applies to your research, read the appropriate section before selecting a response.

Materials & experimental systems

n/a	Involved in the study	
☒	☐	Antibodies
☒	☐	Eukaryotic cell lines
☒	☐	Palaeontology
☒	☐	Animals and other organisms
☐	☒	Human research participants
☒	☐	Clinical data

Methods

n/a	Involved in the study	
☒	☐	ChIP-seq
☒	☐	Flow cytometry
☒	☐	MRI-based neuroimaging

Human research participants

Policy information about studies involving human research participants

Population characteristics	Our study sample comprised a total of 19,996 participants, including 11,497 participants from population- and family-based studies, as well as 8,499 participants from COPD-enriched studies. Using participant self-reported race/ethnicity, 12,316 and 6,450 participants were categorized as non-Hispanic White or African American, respectively, while the remaining 1,224 participants represented Hispanic, Asian and other races/ethnicities. The combined participants were 49% male, and all adults with a median age of 59.5 years (and standard deviation of 10.9 years). The study participants included 4,466 moderate-to-severe COPD cases and 1,739 severe COPD cases. Among these, 1,279 moderate-to-severe and 220 severe COPD cases were contributed by population- and family-based cohorts, and the remaining COPD cases were from the COPD-enriched studies.
Recruitment	Participants in the population- and family-based studies included in our analyses were healthy adults at the time of recruitment. They were primarily recruited as community-based samples residing within the region of the selected study sites that were predominantly associated with large-hospital centers. Accordingly, the resulting population/family-based study samples will be biased toward being healthy indviduals who are willing to participate research that is not heavily focused on a particular disease, and they are generally more representative of urban populations compared to the general US population.<br><br>The COPD-enriched studies include COPDGene and the Boston Early Onset COPD study. COPDGene is a cross sectional prospective cohort enrolled between January 2008 and June 2011 at 21 Clinical Centers, with the goal of carrying out genetic studies of COPD-related traits in smokers with and without COPD. Eligible subjects were of non-Hispanic white or African-American ancestry, aged 45-80 years old, with a minimum of 10 pack-years of smoking and no lung disease (other than COPD or asthma). Boston Early Onset COPD (EOCOPD) study was designed to study genetic factors for early-onset and severe COPD. Probands were selected to be physician-diagnosed COPD cases with FEV1≤ 40% predicted and age ≤ 53.
Ethics oversight	Our study was approved by the IRB at the University of Virginia and Brigham and Women's Hospital, as well as the IRBs at all participating research centers and recruitment sites.

Note that full information on the approval of the study protocol must also be provided in the manuscript.
